# A Novel Pulse-Chase SILAC Strategy Measures Changes in Protein Decay and Synthesis Rates Induced by Perturbation of Proteostasis with an Hsp90 Inhibitor

**DOI:** 10.1371/journal.pone.0080423

**Published:** 2013-11-27

**Authors:** Ivo Fierro-Monti, Julien Racle, Celine Hernandez, Patrice Waridel, Vassily Hatzimanikatis, Manfredo Quadroni

**Affiliations:** 1 Center for Integrative Genomics, University of Lausanne, Lausanne, Switzerland; 2 Laboratory of Computational Systems Biotechnology, École Polytechnique Fédérale de Lausanne (EPFL), Lausanne, Switzerland; 3 SIB Swiss Institute of Bioinformatics, Lausanne, Switzerland; 4 SIB Swiss Institute of Bioinformatics, Vital-IT group, Lausanne, Switzerland; UGent/VIB, Belgium

## Abstract

Standard proteomics methods allow the relative quantitation of levels of thousands of proteins in two or more samples. While such methods are invaluable for defining the variations in protein concentrations which follow the perturbation of a biological system, they do not offer information on the mechanisms underlying such changes. Expanding on previous work [1], we developed a pulse-chase (pc) variant of SILAC (stable isotope labeling by amino acids in cell culture). pcSILAC can quantitate in one experiment and for two conditions the relative levels of proteins newly synthesized in a given time as well as the relative levels of remaining preexisting proteins. We validated the method studying the drug-mediated inhibition of the Hsp90 molecular chaperone, which is known to lead to increased synthesis of stress response proteins as well as the increased decay of Hsp90 “clients”. We showed that pcSILAC can give information on changes in global cellular proteostasis induced by treatment with the inhibitor, which are normally not captured by standard relative quantitation techniques. Furthermore, we have developed a mathematical model and computational framework that uses pcSILAC data to determine degradation constants k_d_ and synthesis rates V_s_ for proteins in both control and drug-treated cells. The results show that Hsp90 inhibition induced a generalized slowdown of protein synthesis and an increase in protein decay. Treatment with the inhibitor also resulted in widespread protein-specific changes in relative synthesis rates, together with variations in protein decay rates. The latter were more restricted to individual proteins or protein families than the variations in synthesis. Our results establish pcSILAC as a viable workflow for the mechanistic dissection of changes in the proteome which follow perturbations. Data are available via ProteomeXchange with identifier PXD000538.

## Introduction

Monitoring protein abundances and their evolution throughout biological processes is critical for understanding the mechanisms by which cellular functions are achieved. The required protein abundances in the cell are maintained through a series of concerted and very tightly regulated processes, including DNA transcription [2], RNA processing and degradation [3] and translation [4] [5], through to the modification [6], localization and degradation (reviewed in [7]) of the protein products. Ultimately, a dynamic balance of protein abundances is accomplished as a result of the coordinated control of all these processes. Strategies for measuring how these processes contribute to attaining an overall dynamic balance of protein abundances have evolved from the quantitation of mRNAs to the direct estimation of protein levels on a high-throughput scale using quantitative proteomics. Until about a decade ago, it was assumed that mRNA levels largely determine protein levels. It is now accepted that post-transcriptional and translational control can lead to a poor correlation between mRNA and protein abundances (reviewed in [8], [9]).

The development of proteomics techniques during the last decade has given investigators the capacity to systematically quantitate net changes, or relative abundances of thousands of proteins (reviewed in [10]). Notably, the introduction of metabolic stable-isotope labeling techniques for quantitation, especially SILAC [11], [12], has offered us a powerful and accurate tool for the analysis of changes in the proteome. In its standard version (stSILAC), SILAC compares in a mixture a completely “heavy” labeled proteome from a cell culture (first condition), with an unlabeled “light” proteome derived from another cell culture (second condition).

Although standard quantitative proteomics techniques are essential to identify and measure variations in protein abundances, they are not adapted to determine if such variations are due to changes in synthesis rates, decay rates, or a combination of both. Nor are these techniques able to quantitate other properties such as turnover, which may give an indication of the intrinsic stability and half-life of proteins. Now, the understanding of the mechanism(s) underlying variations in protein levels can provide information that can be important to interpret the changes observed and formulate hypotheses on the pathways involved.

Kinetic parameters of proteins, such as rates of synthesis and degradation, turnover, and half-life, have been classically studied by metabolic pulse- and/or chase labeling experiments using ^35^S-methionine (i.e. [13]–[16]). Radioactive labeling offers remarkable sensitivity, but imposes to focus on one or a few proteins of interest to obtain accurate quantitation, and is thus hardly suitable for high-throughput studies.

In recent years, metabolic labeling with stable isotopes has been increasingly used for measuring protein turnover, most often by culturing cells for a limited time in media containing stable isotope-labeled amino acids, resulting in partial (“pulse”) labeling. After extraction, digestion, and identification of proteins by tandem MS, the heavy/light ratios determined for peptides provide the level of label incorporation during the time frame considered. In steady-state systems, where the concentration of all proteins is assumed to be constant, incorporation ratios allow to measure protein turnover and half-life. Protein turnover analysis has been pioneered by the group of R. Beynon, who established theoretical principles and carried out both *in vitro* and *in vivo* studies [17]–[19]. SILAC-like pulsed labeling was later used for targeted analyses to measure the flux of ribosomal proteins in nucleoli [20], [21] and to determine turnover for several hundred of blood and tissue proteins in the mouse [22], yielding an overview of *in vivo* protein turnover. Total cell labeling with ^15^N has also been used for turnover measurements in both mammalian and microbial systems [23]–[27]. Although most turnover studies relied on isotope labeling and mass spectrometry, GFP-tagging and other classical biochemical approaches have also been used to determine turnover [28]–[30]. SILAC based strategies are nevertheless preferred for the large scale analysis of protein levels and protein turnover [31] [32]. In turn, although protein turnover can be an informative parameter measurable in a steady-state system, a more comprehensive analysis of the dynamics of protein levels should aim at determining the mechanistic components involved, i.e. protein synthesis and decay rates, in the steady state and/or in a perturbed system. The “pulsed SILAC” (pSILAC) method was a first step in this direction, developed to compare levels of newly synthesized proteins in two conditions by implementing a 3-channel labeling strategy, where labeling was performed with three isotopomers of arginine [1], [33]. Interestingly, the technique was applied to study the influence of microRNAs (miRNAs) on global levels of translation [34]. With a different focus and using an innovative SILAC labeling scheme, rates of protein synthesis, decay, and turnover, were determined in HeLa cell extracts, as well as in separate cytoplasmic, nuclear, and nucleolar compartments [35], [36]. Moreover, Japayal et al [37] combined SILAC with iTRAQ (Isobaric Tags for Relative and Absolute Quantitation) labeling to measure turnover and degradation rates in *Streptomyces coelicolor*.

In spite of the significant progress made so far, most of the work focusing on protein kinetic parameters was aimed at analyzing a single, steady-state system. The goal of the present work was to develop a technique which could yield, within the same experiment, information on protein synthesis and decay rates in two conditions, a steady-state and a perturbed cellular system. To test and validate our method we needed a model cellular system which, upon perturbation, undergoes numerous known changes in both protein synthesis and degradation. We chose to study the inhibition of the Hsp90 molecular chaperone by the ansamycin antibiotic geldanamycin (GA). Hsp90, a highly conserved chaperone, is believed to be one of the master regulators of proteostasis in eukaryotic cells under physiological or stress conditions (reviewed in [38]). Hsp90 interacts with a large array of cellular proteins, which depend on it to reach their active conformation. A critical stabilizing interaction is assumed between Hsp90 and its “clients”, defined as a set of proteins strongly dependent on Hsp90, of which many take part in signaling pathways. Treatment of cells with specific inhibitors of Hsp90, such as GA, induces the depletion of many or most Hsp90 “clients”, mediated mostly by an increased degradation [39]. At the same time, Hsp90 inhibition induces a generalized protein folding cellular stress that triggers an increase in synthesis of molecular chaperones [32], [40]–[42], in a mode similar to the heat shock response [43].

Here, we describe a novel technique called “pulse-chase SILAC” (pcSILAC), which yields comparative data on both protein decay and novel protein synthesis in two conditions. When tested on Hsp90 inhibition of a cellular system, increased decay of Hsp90 clients and increased synthesis of stress response proteins were detected within the same pcSILAC experiment. Using this technique, we performed global, as well as individual protein assessment of the dynamics of protein decay and synthesis in both control and inhibitor-treated cells. Moreover, we developed a mathematical framework, tailored to pcSILAC, to calculate protein degradation constants (and hence half-lives) and synthesis rates for both cellular states.

## Experimental Procedures

### Materials

All isotope labeled amino acids were from Cambridge Isotope Laboratories (CIL), Andover, MA, USA. The specially formulated media for SILAC were from Cell Culture Technologies, Gravesano, Switzerland, while dialyzed fetal bovine serum (FBS) and antibiotics were from Invitrogen (Carlsbad, CA, USA). Sequencing grade modified Trypsin was from Promega. All other chemicals were from Sigma-Aldrich.

### Methods

**Experimental design.** Two independent pcSILAC experiments were performed, both of which included duplicates with inversion of labels and extraction of total mRNA at two time points (t = 5h, 19h) and protein at three time points (t = 6, 12, 20h). The two experiments, described in Figure S1, differed in that in experiment 1 GA was added immediately after medium exchange (t = 0), while in experiment 2 the inhibitor was added 2h later. For this reason, their results are presented separately. The main text mainly presents data from experiment 2 (full data are reported in Tables S1, S3 and S4), while similar plots for experiment 1 are given in the supplementary figures and the data in Tables S1, S2 and S4.

**Figure 1:**
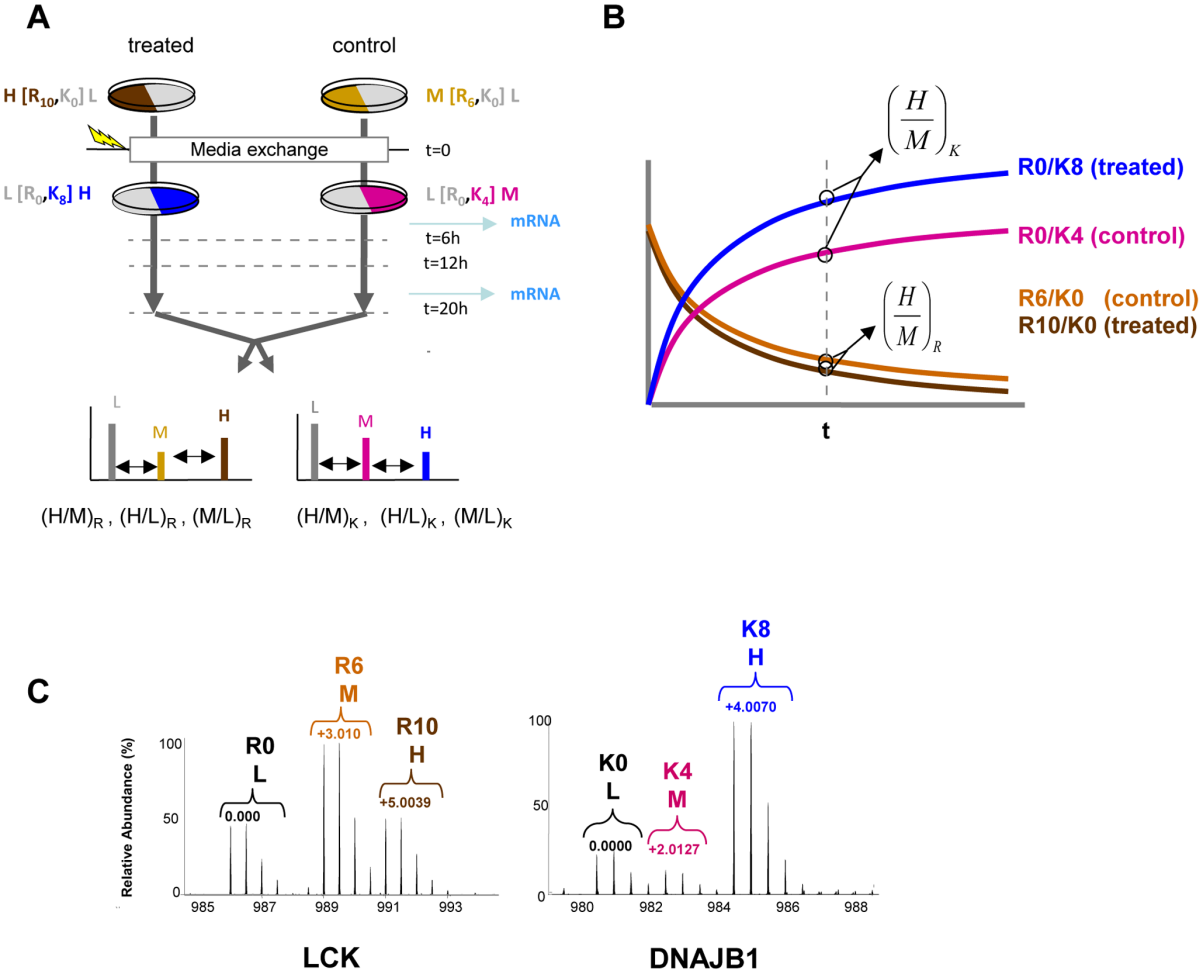
Labeling, measurable parameters and data obtained from pcSILAC experiments. **A**) Protein labeling scheme for pcSILAC experiments. Two cell cultures were fully labelled only on Arg residues before cell treatments. For example, cells to be treated later with HSP90 inhibitor were fully labelled with ^13^C_6_^15^N_4_-L-arginine (R10/K0) (“heavy” cells) while cells to be used as control were fully labelled with ^13^C_6_-L-arginine (R6/KO) (“medium” cells). At the start of the experiment, “heavy” cells were transferred into a medium containing light R (R0) and “heavy” K (^13^C_6_^15^N_2_-L-Lys, referred to as R0/K8 medium), while “medium” cells were transferred to a medium containing light R (R0) and “medium” K (^2^H_4_-L-lysine, referred to as R0/K4 medium). GA or DMSO as a control were added either simultaneously or later, depending on the experiment. Cells were harvested and lysed at various time points to produce extracts that were mixed before analysis. **B**) Conceptual view of the levels of a hypothetical protein in a mixture of two (control and treated) pcSILAC-labeled samples. Pre-existing protein is fully labeled R6/K0 and R10/K0 (light and dark brown), respectively for the control and treated sample. Newly synthesized protein is labeled R0/K4 (control, pink) and R0/K8 (treated, blue). The SILAC (H/M) ratios for R- and K-containing peptides therefore measure the ratios of preexisting and newly synthesized proteins at time t. **C)** examples of pcSILAC spectra for peptides from the tyrosine kinase LCK (left, peptide IPYPGMTNPEVIQNLER at t = 6h, z = 2) and the chaperone DNAJB1 ( = Hsp40) (right, peptide EGDQTSNNIPADIVFVLK at t = 20h, z = 2).

**Pulse-chase SILAC and standard SILAC experiments.** Jurkat T-lymphocytes clone J77.20 were a kind gift of Dr. Oreste Acuto, University of Oxford and have been previously described [44], [45]. Cells were cultured in Roswell Park Memorial Institute (RPMI) 1640 medium with 10% (v/v) dialyzed FBS for all experiments. In the pcSILAC labeling scheme (Fig.1 and Fig. S1), pre-exchange medium (PEM) was used to culture and fully label the cells. After medium exchange labeling proceeded by culturing cells in post-exchange medium (POM). Stable Isotope labeled amino acids were included in the ‘heavy-pcSILAC PEM’ (containing ^13^C_6_^15^N_4_-L-arginine) and in the ‘medium-pcSILAC PEM’ (^13^C_6_-L-arginine) at 100 mg/l, whereas proline was supplied at 180 mg/l, that is a 9-fold excess over its standard concentration in RPMI medium. Labeling under pre-exchange conditions was achieved by culturing the cells for a minimum of 2 weeks to allow for at least 5 cell divisions. Before start of the experiments, tests were carried out to verify that heavy labeling was >98% and Arg to Pro conversion was lower than 5%. Cells were centrifuged and rinsed with PBS (phosphate buffered saline) before transfer from heavy-pcSILAC PEM to heavy-pcSILAC POM (same as heavy-pcSILAC PEM, but containing^13^C_6_^15^N_2_-L-lysine and light arginine), or from medium-pcSILAC PEM to medium-pcSILAC POM (same as medium-pcSILAC PEM, but containing ^2^H_4_-L-lysine and light arginine). After the exchange of media, heavy-pcSILAC labeled cells were treated with 1 μM geldanamycin, while medium labeled cells were treated with the same volume of DMSO. Further culturing of the cells in POM (after drug treatment) was conducted for 20h, and aliquots of cells were taken at 6 and 12h after drug treatment. The experiment consisted of two independent biological replicates and an additional stSILAC control, respectively. In the second replicate, the treatment was inverted, i.e. GA was added to medium labeled cells (Fig. S1 for experimental design). For determination of growth, cells were counted using a Neubauer cell in triplicate at t = 0 and t = 20h for each biological replicate (cell numbers are reported in Table S1). Viability was assessed by trypan blue exclusion.

**Cell lysis, protein extraction, digestion and fractionation.** Cells harvested from any of the previously described media were rinsed 3 times with PBS before lysis. Cells were lysed in 4% SDS, 100 mM Tris/HCl pH 7.5, 100 mM DTT followed by heating at 95°C. After centrifugation, protein concentrations were measured by gel electrophoresis, coomassie blue staining and densitometry with comparison against a pre-quantitated standard cell extract. Equimolar extracts from light/heavy or heavy/medium labeled cells were combined, alkylated with iodoacetamide and digested using the filter assisted sample preparation (FASP) method as described [46]. The obtained peptide mixtures (200 µg total material) were desalted on SepPak C18 cartridges (Waters Corp., Milford, MA), dried, dissolved in 4M Urea with 0.1% Ampholytes pH 3–10 (GE Healthcare) and fractionated by off-gel focusing as described [47]. The 24 fractions obtained were desalted on a micro tSepPak C18 96-well plate (Waters Corp., Milford, MA), dried and resuspended in 0.1% formic acid, 3% (v/v) acetonitrile for LC-MS/MS analysis.

**Mass spectrometry analysis.** Digests were analyzed on a hybrid linear trap LTQ-Orbitrap Velos mass spectrometer (Thermo Fisher, Bremen, Germany) interfaced via a nanospray source to a Dionex RSLC 3000 nanoHPLC system (Dionex, Sunnyvale, CA, USA). Peptides were separated on a reversed-phase Acclaim PepMap nano-column (75 µm ID×15 cm, 2.0 µm, 100 A, Dionex) with a gradient from 5 to 85% acetonitrile in 0.1% formic acid (total time: 120 min). Full MS survey scans were performed at 60’000 resolution. In data-dependent acquisition controlled by Xcalibur 2.1 software (Thermo Fisher), the twenty most intense multiply charged precursor ions detected in the full MS survey scan were selected for CID fragmentation in the LTQ linear trap and then dynamically excluded from further selection during 120s. The window for precursor isolation was of 3.0 m/z units around the precursor. The mass spectrometry proteomics data have been deposited to the ProteomeXchange Consortium (http://proteomecentral.proteomexchange.org) via the PRIDE partner repository [48] with the dataset identifier PXD000538.

**MS data analysis: identification and quantitation.** MS Data were analyzed and quantitated using MaxQuant version 1.0.13.13 [49], combined with Mascot (Matrix Science, London, UK) version 2.3. Peak lists were generated with MaxQuant with standard parameters. Database searches were performed on the IPI (International Protein Index) Human database (version 3.68), filtered to keep only the entries mapping a Swiss-Prot identifier (34743 entries actually searched) in order to maximize sequence and annotation quality in further analysis steps. Cleavage specificity was trypsin (cleavage after K, R, no cleavage at KP, RP) with two missed cleavages. Mass tolerances were of 7 ppm for the precursor and 0.5 Da for CID tandem mass spectra. The iodoacetamide derivative of cysteine was specified as a fixed modification, and oxidation of methionine and protein N-terminal acetylation were specified as a variable modifications. Protein identifications were filtered at 1% false discovery rate (FDR) established by MaxQuant against a database of reversed sequences. A minimum of one unique peptide was necessary to discriminate sequences which shared peptides. Sets of protein sequences which could not be discriminated based on identified peptides were listed together as protein groups and are fully reported in the tables S1, S2 and S3. Details of peak quantitation and protein ratio computation by MaxQuant are described elsewhere [50]. All proteins with quantitated values (minimum evidence count = 1) were initially retained to be subjected to filtering in subsequent steps (see below).

**pcSILAC-specific data analysis.** All the subsequent filtering steps were carried out automatically with a pipeline of custom-made Perl scripts (Perl v5.10.1, scripts available in File S1). Protein group tables from MaxQuant were further processed to remove contaminants annotated in the database and matches to reverse sequences. Further 63 proteins from an internal laboratory list of 65 environmental contaminants were also removed. Next, the proteinGroup.txt table and the evidence.txt table were then used as input for the *discriminate_KR_peptides.pl* (file S1) script. The script extracted all quantitation values (normalized and non-normalized) from the evidence file, separated quantitation values of K- and R-containing peptides and calculated log_2_ of medians for K and R “channels” for each protein group in each experiment. Mixed-label peptides (containing both K and R) were discarded. When needed, normalization for each experiment was checked and adjusted by translation based on the median. Further on, data values with less than 3 evidence counts were removed from the tables.

**Removal of subsets, update of protein annotation and alignment of datasets.** To simplify post-analysis comparisons of pcSILAC and standard SILAC datasets, subset proteins (identified with a subset of peptides) were removed from the protein groups. To ensure that the UniProt, GO (Gene Ontology), KEGG (Kyoto Encyclopedia of Genes and Genomes) and PFAM (Protein FAMilies) annotations were completely up-to-date, a Perl script (File S1) automated REST (REpresentational State Transfer) requests to the UniProt (http://www.uniprot.org/uniprot/) and EBI websites (http://www.ebi.ac.uk/QuickGO/GAnnotation) and SOAP (Simple Object Access Protocol) requests to the KEGG website (http://soap.genome.jp/KEGG.wsdl), as well as re-import of these data into the result table on the basis of the simplified protein groups. All sequences identified with the same number of peptides were retained in the protein groups. When necessary, protein groups in analyzed datasets from pcSILAC and standard SILAC were matched by IPI identifiers. Only when all IPI identifiers in a protein group were identical in two datasets, the protein groups and quantitative values were matched and aligned in a joint table.

**Modeling and computational steps.** Construction of the mathematical model and computational steps are described in the results section and in detail in supplementary information S1.

**Other data analysis steps.** Enrichment analysis of kinetic parameters was performed with the 1D annotation enrichment algorithm of the software package Perseus as described [40] on Gene Ontology (GO), KEGG and PFAM terms. The Benjamini-Hochberg correction with threshold at 0.02 was applied to filter GO enrichment results. Plots and further analysis were performed with the R software package.

## Results

### Cellular system and experimental setup

Jurkat cells (derived from a T-cell leukemia) were grown in RPMI medium modified for SILAC labeling and then treated with 1 µM GA. Under these conditions, drug treatment reduced cell proliferation (table S1) and induced a cell cycle arrest but did not cause detectable levels of apoptosis within the time frame of the experiment (Fierro-Monti et al., accompanying manuscript).

### Description and conceptual view of the pulse-chase SILAC strategy

In classical pulse-chase experiments, cells are grown for a short time in a medium containing ^35^S-methionine to radioactively label proteins produced in the selected time frame (pulse). After this, the cells are transferred into a medium containing an excess of unlabeled amino acid and analyzed after variable times (chase). The labeling allows tracing the subsequent evolution of the abundance of a temporally defined pool of radioactive protein(s) without interference by the polypeptides synthesized later. A general concept in pulse and chase experiments is thus the possibility to differentiate, through the incorporation of labeled amino acids, pre-existing proteins from proteins synthesized after medium exchange. The application of this concept using stable isotope labeled amino acids and MS detection has resulted in the pulsed SILAC (pSILAC) strategy, which can measure the amount of newly synthesized protein accumulating after a medium exchange or, more precisely, the ratio new/old protein at a given time. Such a label replacement ratio can be used, if the system can be assumed to be at steady-state i.e. has constant protein concentrations, to calculate protein half-lives. pcSILAC takes the concept one step further, by measuring at the same time the levels of new proteins and the evolution of the levels of pre-existing proteins. Furthermore, through SILAC multiplexing and amino acid encoding as it is done in pcSILAC, levels of new/old proteins in two cellular conditions can be compared in the same experiment. The main difference with the classical radioactive pulse-chase is that in pcSILAC the pulse and chase measurements are performed on distinct pools of proteins, while in the classical scheme they concern one and the same population.

The labeling and treatment scheme for pcSILAC is described in detail in Figure 1 and Figures S1-S2. Briefly, two cell cultures to be compared are fully labeled with, respectively, “medium” (^13^C_6_-L-arginine, R6) and “heavy” (^13^C_6_^15^N_4_-L-arginine, R10) Arg, while Lys is left unlabeled (“light”, K0) for both. At the start of the experiment, both cultures are transferred to new media containing light Arg (R0) to perform the chase of R-labeled pre-existing proteins. In turn, the new media contain, respectively, “medium” (“M”, ^2^H_4_-Lysine, K4) and “heavy” (“H”, ^13^C_6_^15^N_2_-L-lysine, K8) Lys, which will label all newly synthesized proteins for the pulse measurements. At the same time or after medium exchange, one of the cultures is subjected to a perturbation (here, drug treatment). Extracts from control and drug-treated cells are then harvested at defined time points, mixed equimolarly, digested with trypsin and analyzed.

One peculiarity of the pcSILAC labeling scheme is that, unlike in standard SILAC, Arg (R) and Lys (K) encode different measurements, in this case the chase and the pulse, respectively. Therefore, the quantitative information provided by peptides containing labeled K or R is different and has to be kept separate at the protein level. From the triplex isotope patterns observed in pcSILAC six ratios (Figure 1) are derived for every protein at each time point. The most directly interpretable ratios are the “heavy” (H) over “medium” (M) ratios, (H/M)_R_ and (H/M)_K_. The (H/M)_R_ (R10/R6) ratio describes the evolution in time of the relative levels of pre-existing proteins, because very little or no protein labeled with heavy or medium R should be produced after medium exchange. Therefore, in the final mixture the (H/M)_R_ ratio at t>0 should mainly reflect the relative decay rates of proteins in the treated vs. control sample (Figure 1B). Simultaneously, as for “regular” pSILAC measurements [31], the (H/M)_K_ ratio should express the relative levels of proteins newly synthesized and accumulating in the two samples after medium exchange at t = 0 (Figure 1B). Since newly synthesized proteins can also be subject to decay, the (H/M)_K_ (i.e. K8/K4) ratio is expected to reflect the combination of changes in protein synthesis and in decay rates. While the (H/M) ratios which are related to the comparison of the two conditions are the easiest to interpret, the other measured ratios (H/L) and (M/L) also carry information (e.g. related to protein turnover) and can be exploited to derive other parameters.

### Experimental design and quality control of protein labeling

Two independent pcSILAC experiments were performed (Fig.S1), both of which included extraction and analysis of protein at three time points (t = 6, 12, 20h). Experiment 1 also included a standard SILAC control experiment using the same cells and with quantitation only on Arg (H/M). As a quality control of the labeling process, we inspected in detail the isotope envelopes of K- and R-containing tryptic peptides and the replacement kinetics of both labeled amino acids (Figures S1-S5). It appeared that label replacement in time was complete, resulting in minimal mixing of old and new label. We could also determine that i) mixed-labeled peptides containing both K and R were unsuitable for quantitation and that ii) a slight difference was present in the (H/L), resp. (M/L) ratios measured on K- vs. R-containing peptides, which had to be accounted for in further steps (see supplementary information S1).

### Coverage and reproducibility of pcSILAC data

Due to the triplex labeling (each peptide is present as 3 peaks), the rejection of mixed-labeled peptides and the separation of K- and R-based quantitation, pcSILAC experiments typically resulted in lower proteome coverage than comparable (duplex) stSILAC measurements. While more than 3400 proteins were identified in the samples in pcSILAC experiments 1 and 2, after stringent filtering respectively only 539 and 911 proteins quantified with all 6 ratios at all three time points were retained for further calculations. As an indicative comparison, a similar (but duplex) stSILAC experiment resulted in 2695 proteins quantitated at two time points in three replicates (Fierro-Monti et al., accompanying manuscript). Reproducibility between pcSILAC experiments was assessed at 20h on various ratios. Values of Pearson’s correlation coefficient *r* between medians of replicates of experiments 1 and 2 were generally above 0.85, indicating good reproducibility (figures S1, S6).

### Reference stress proteins and Hsp90 clients show expected trends in pcSILAC data

**Figure 2:**
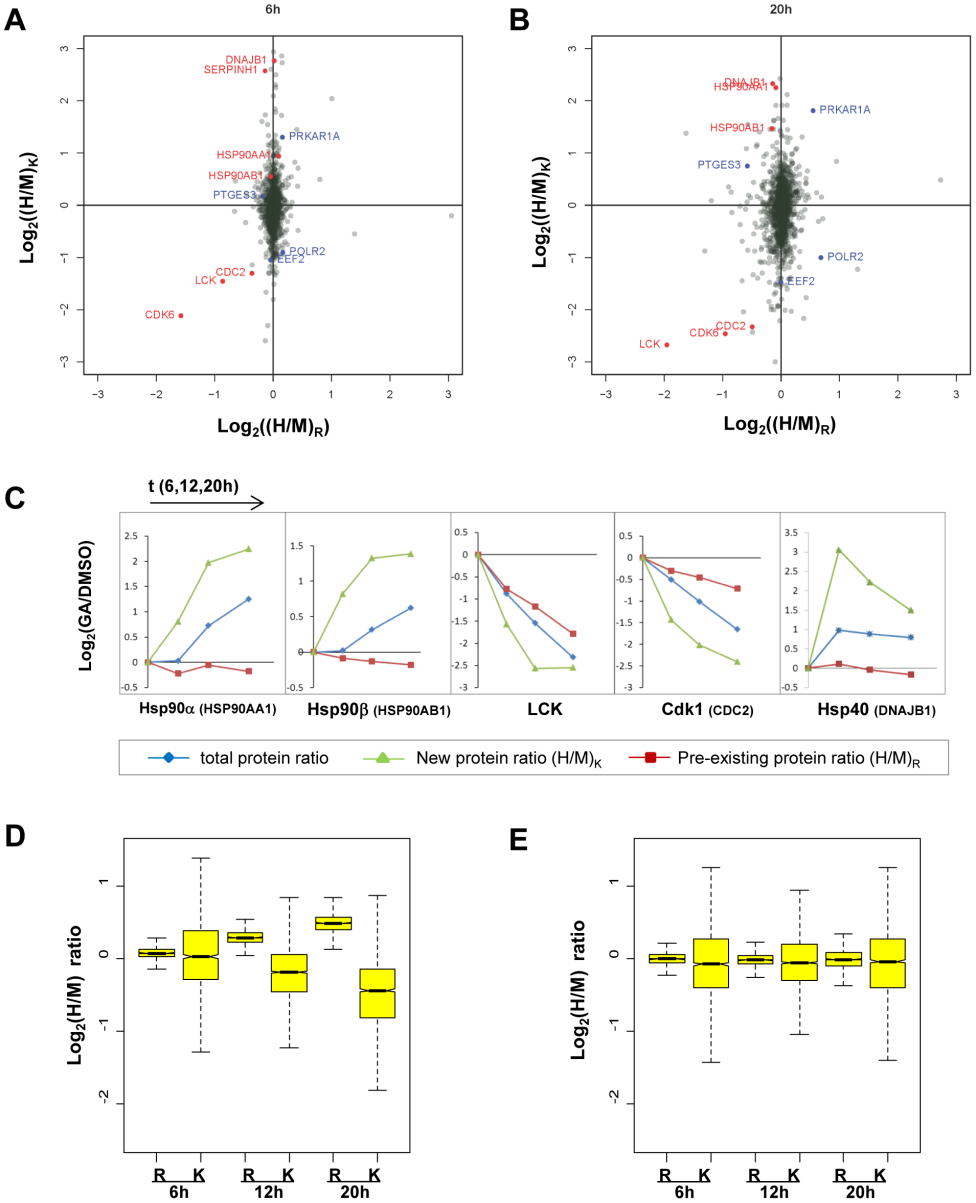
Raw and normalized (H/M)_K_, (H/M)_R_ ratios measured in pcSILAC experiments. **A**),**B**) Scatter plots of global ratios of pre-existing protein ((H/M)**_R_**) and newly synthesized protein ((H/M)**_K_**) after correction for mixing ratio and normalization around the median. Values shown are for pcSILAC experiment 1 (average of two replicates) at t = 6h (A) and t = 20h (B). Reference (red) and other proteins discussed in the text (blue) are indicated. **C**) Evolution in time after start of GA treatment of normalized ratios of total protein (stSILAC, blue), newly synthesized proteins ((H/M)_K_, green) and pre-existing protein ((H/M)_R_, red). Data points refer to values at t = 6,12,20h after addition of GA (t = 0). pcSILAC ratios were normalized (i.e. centered around population median) to facilitate comparison with changes in total protein levels. Fitting of the values for DNAJB1 to the model was very poor due to its complex behavior, therefore decay and synthesis rates could not be calculated for this early induced protein. **D**) Box plots (experiment 1) of global log_2_(H/M)**_K_** and log_2_(H/M)**_R_** ratios after mixing ratio correction but before normalization **E**) same as D), but values are shown after correction for mixing ratio and normalization.

As described above, the ratios (H/M)_R_ and (H/M)_K_ are the most directly interpretable pcSILAC data. These ratios reflect a direct comparison of the relative protein abundances in the treated (heavy labeled) to the untreated (medium labeled) cell cultures. Again, the (H/M)_R_ (i.e. R10/R6) ratio measures the relative levels of pre-existing protein remaining at time t (Figure 1B). By contrast, the (H/M)_K_ (i.e. K8/K4) ratio reflects the relative levels of proteins newly synthesized and accumulating from *t* = 0 until time t. We selected some reference proteins with known behavior following Hsp90 inhibition in order to validate qualitatively pcSILAC data. Cytosolic Hsp90 proteins (Hsp90α; HSP90AA1 and Hsp90β; HSP90AB1) as well as Hsp40 (DNAJB1) and Hsp47 (SERPINH1) are among the stress response proteins whose levels increase by transcriptional activation upon GA treatment as well as heat shock [41] [51]. Quantitation of transcripts confirmed that the mRNAs for these and other chaperones were increased in GA-treated cells (Fierro-Monti et al., accompanying manuscript). Consistent with their known mechanism of change, these and several other chaperones displayed (H/M)_K_ ratios greater than 1 (log_2_ values positive) together with (H/M)_R_ ratios close to 1 (log_2_ values near 0), indicating increased net protein synthesis in GA-treated cells, with only small (but sometimes significant, see below) changes in protein decay (Fig.2A-C). For these increasing chaperones, the increase in newly synthesized protein seems to drive the rise in total level observed in GA-treated cells (Fig.2C). As reference proteins for the detection of decay we choose the protein kinases LCK, CDC2 ( = Cdk1) and CDK6, known Hsp90 “clients” that are increasingly degraded upon Hsp90 inhibition [52]–[56]. Consistent with these previous reports, all three proteins displayed negative log_2_(H/M)_R_ values, suggesting an increased decay of the pre-existing protein pool in GA-treated cells (Fig. 2A-C). Not unexpectedly, the amount of newly synthesized protein was even more strongly affected by GA and showed a sharp decrease. For LCK, both findings are consistent with the results of Yorgin et al [57] who showed an impact of Hsp90 inhibition on both mature and newly synthesized forms of the kinase, with a stronger reduction in levels of the latter. From the values for these reference proteins we concluded that, at least qualitatively, pcSILAC measurements can reveal both changes in protein decay and protein synthesis rates.

### Distributions of pcSILAC ratios hint at global changes in proteome turnover

In pcSILAC, as in general in SILAC, approximately equal amounts of total proteins extracted from control and treated cells were used for the experiments. Each pcSILAC cell extract sample was constituted of pre-existing proteins (with "old" label) and newly synthesized proteins (with "new" label) in unknown proportions. The (H/M) pcSILAC ratios directly compare between conditions the amounts of pre-existing ((H/M)_R_) and newly synthesized ((H/M)_K_) proteins. Therefore, the distribution of raw (not normalized) values of these ratios can potentially give information on changes in global protein turnover caused by the treatment. A comparison of the distributions of (H/M)_R_ and (H/M)_K_ raw ratios before normalization revealed that the population of (H/M)_K_ ratios – measuring newly synthesized proteins, with H being the treated (GA) and M the control (DMSO) sample - consistently showed lower values than the one of pre-existing protein ratios ((H/M)_R_) (Fig. 2D-E, Figure S8). This implies that the global ratio of new over pre-existing protein was different in the two samples. More specifically, with the same amount of total protein the treated sample contained less newly synthesized protein and more pre-existing protein than the control sample, and the differences increased with time, suggesting global changes in protein synthesis and/or decay induced by the inhibitor. The second global observation was that (H/M)_R_ ratios showed in all replicates and at all time-points a much narrower distribution than (H/M)_K_ ratios (Fig. 2D-E, Fig. S7 and S8). This suggests that, at least globally, Hsp90 inhibition had a bigger impact on the levels of newly synthesized proteins than on those of pre-existing proteins. These two global observations and their functional, biological implications are discussed more in detail below and elsewhere (Fierro-Monti et al., accompanying manuscript).

### The relationship between pcSILAC ratios and net protein levels (standard SILAC ratios) is complex

pcSILAC can measure the ratios of levels of pre-existing ((H/M)_R_) and newly synthesized proteins ((H/M)_K_), respectively in the treated (H) vs. control (M) cells. The relationship between the values of these ratios and the change in total protein levels can be expected to be complex, due to the combination of effects on synthesis and decay. For strong changes in (H/M)_R_ and/or (H/M)_K_, such as those observed for the reference proteins discussed above, a net protein increase (HSP90AA1( = Hsp90α), DNAJB1), and respectively a decrease (LCK, CDK6, Cdk1) can be confidently predicted. Correlating pcSILAC data with net protein changes appears less straightforward for proteins whose values of (H/M)_R_ and (H/M)_K_ indicate contrasting trends, such as a decrease in stability coupled with a higher synthesis (PTGES3, the Hsp90 cofactor p23) or a stabilization with lower synthesis (POLR2). A global representation of the stSILAC ratios versus (H/M)_R_ and (H/M)_K_ ratios, from a stSILAC control prepared within the same experiment as the pcSILAC, suggested indeed that for a similar net protein change (stSILAC), different proteins can display different combinations of (H/M)_R_ and (H/M)_K_ (Fig. S9). We addressed the complex relationship between (H/M)_R_ and (H/M)_K_ and stSILAC (H/L) ratios through a mathematical treatment, whose steps are described below and in the supplementary information.

### pcSILAC data can be used to calculate net protein changes

Given the pcSILAC labeling scheme, each protein is expected to be present as 4 differently labeled species, represented by the four variables *U, V, X,Y*:


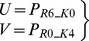
in culture A (DMSO and 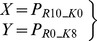
in culture B (GA) (1)

Thus U and X represent the amounts of proteins synthesized before label exchange, while V and Y those produced after label exchange. In turn, *U, V, X,Y* are related to the measured pcSILAC ratios by simple algebraic relationships such as (H/M)_R_  = *X/U* and (H/L)_R_  = *X/(Y+V).* From these, it is possible to obtain the incorporation ratio, i.e. the ratio “new label”/“old label”, at a given time point. More importantly, we could show that it is possible to calculate from pcSILAC data the net ratio 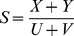 (equivalent of a stSILAC ratio) for any protein at a given time (supplementary information S1). The derived values of *S* were found to be in very good correlation (Fig. S10) with experimental values obtained from an “internal” stSILAC replicate for pcSILAC experiment 1 (Pearson’s *r*  = 0.94), as well as with ratios from an external stSILAC experiment (Pearson’s *r*  = 0.91, Fierro-Monti et al., accompanying manuscript). The possibility to obtain accurate net protein changes from pcSILAC ratios was an important internal control and showed that the pcSILAC data correctly reflect the changes occurring in the system.

### Model development and calculation of kinetic parameters

Having verified the reproducibility and representativeness of the data, we developed a mathematical model to calculate kinetic parameters for large numbers of proteins from pcSILAC data. The model used to describe the evolution of protein concentrations in control and treated cells was based on the following assumptions: i) before the beginning of the experiment (t = 0) the two cultures are at steady state and thus have identical concentrations of all proteins which remain constant in time; during the experiment (t>0) only the control culture keeps the same steady state ii) the system can be described by first-order rate decay and synthesis terms; iii) kinetic parameters of proteins change much faster than the protein concentrations when drug is added to cultures at t = t_d_, and remain constant thereafter (hence the change of these kinetic parameters is assumed instantaneous on the scale of the experiment) iv) the total global protein concentration in the cell is the same in both cultures and remains constant during the experiment (only the relative abundances of individual proteins vary between the cultures); v) each protein is present in the cell as a homogeneous pool, whereby the synthesis and decay rate constants are only dependent on the protein species and not the protein localization or folding state.

In order to describe the evolution of the protein concentrations in the cell, we adopted a standard ordinary differential equation (ODE) which is often been used for such purposes [58], [59]. This equation is directly derived (see supplementary information S1) from the mass balance equation, and describes for example the evolution of the concentration of a protein p in sample A as: 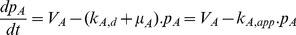
(2)


where *V_A_* and *k_A,d_* are, respectively, the synthesis rate and the decay rate constant for the protein *p_A_*; *μ_A_* corresponds to the cell growth rate and is accounting for the dilution of proteins, and *k_A,app_* is an apparent decay rate, defined as
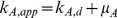
(3)


The same equation (2) can be used to describe the evolution of concentrations in sample B (treated cells). It is important to note that, while the control sample A is assumed to be at steady-state with constant protein concentrations, the treated sample B is a non-equilibrium system which evolves toward a new steady-state during the experiment, and as such possesses more degrees of freedom.

**Figure 3:**
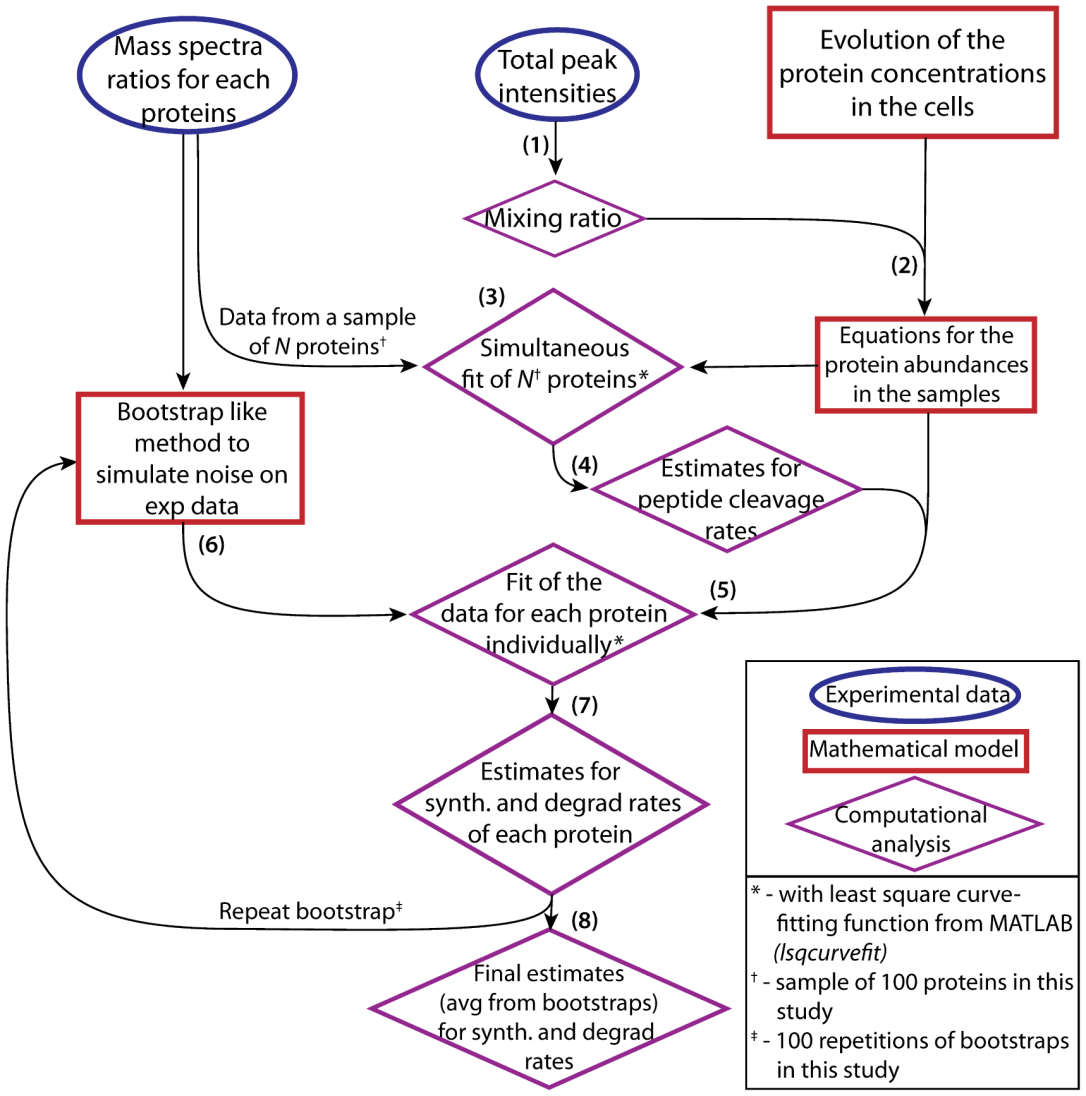
Computational workflow for the determination of kinetic parameters k_d_ and V_B_/V_A_ from pcSILAC data. The individual steps of the computational workflow are described in detail in the supplementary information S1. Briefly, (1) using the total peak intensities (similar to iBAQ scores), the global mixing ratio between both samples is computed. (2) The equations describing the evolution of the protein concentrations in the cells are converted into equations describing the evolution of the protein abundances using the mixing ratio values. (3) These equations can then be used for a global fit of the experimental data for multiple proteins at the same time. (4) The global fit results in estimates for the coefficients describing the peptide cleavage rates (or efficiencies) - these coefficients are then set to be identical for all protein species. (5) The estimates of the cleavage efficiencies are inserted into the model describing the evolution of protein abundances in the samples; the experimental data from each protein species can then be fitted, for each protein species independently. (6) Instead of directly doing these fits to the experimental data, some random artificial noise is added through a bootstrap-like method - this increases the confidence in the results (as noise cannot be avoided in the experimental measurements, and we repeat the fit with various simulated noise values to compare the outcomes). (7) The fit of the model to the bootstrapped data results in one set of estimates of synthesis and degradation rate constants for the normal and drug-treated conditions for each protein. (8) The final estimates are the average values from the different estimates that were obtained by repeating the fitting step multiple times with different bootstrapped data values for each protein species.

Based on the fundamental equations (1) – (3), it was possible to derive and solve ODE’s for the four variables *u, v*, *x* and *y (*obtained from *U,V,X,Y* after normalization for unequal mixing of protein extracts*)*, thus obtaining analytical expressions describing the evolution of the system as a function of time (u(t), v(t), x(t), y(t)). Then these equations were used to determine the kinetic parameters by fitting the equations to the data. For the fitting, additional corrections were performed to account for sample mixing ratios and trypsin cleavage efficiencies differences (supplementary information S1). Several steps of fitting were then used to first determine some global coefficients for the correction factors and then the kinetic parameters for each protein (see Figure 3 for a flow chart of the computational method and supplementary information S1). An implementation of this procedure into MATLAB is available upon request to J. Racle and V. Hatzimanikatis. Importantly, the quality of the fitting and convergence of the data were monitored and only proteins with data fitting the model well were retained, which was the case for most of proteins (Figure S12). However, a small set of proteins, among which DNAJB1 (Fig. 2C), consistently showed a complex multi-phasic evolution in time which could not be appropriately accounted for by the model (Fig. S16). Kinetic parameters could therefore not be calculated for these proteins.

The first output of the calculations (Table S4) is the apparent degradation constants for proteins in the control and drug treated cells, *k_A_app_* and *k_B_app_*. The real “specific” degradation constants *k_A,d_* and *k_B,d_* can be deduced from these through a correction considering the growth rate (equation (3) and supplementary information S1). *k_A,d_* and *k_B,d_* can be used to calculate protein half-lives in both the control and the treated cells. The second main output of the calculations is the ratio of synthesis rates V_B_/V_A_. From the obtained values of *k_A,d_* and *k_B,d_* and V_B_/V_A_, it is possible to calculate directly the expected steady-state (with t→∞) ratio of net protein concentrations *p_B_/p_A_*. Furthermore, we integrated in our workflow information on pseudo-absolute protein amounts calculated with intensity based absolute quantification (iBAQ) scores and used it to infer values for V_A_, V_B_, p_A_, p_B_ in arbitrary units. Finally, we calculated changes in fluxes of synthesis and decay induced by the GA treatment. Fluxes combine k_d_, V and the dilution term μ with protein concentrations and give the possibility to compare the quantitative relevance of each kinetic term, especially at early time points after the start of the perturbation (t = t_d_) (supplementary information S1).

### Reference proteins display the expected changes in decay constants and synthesis rates

**Figure 4:**
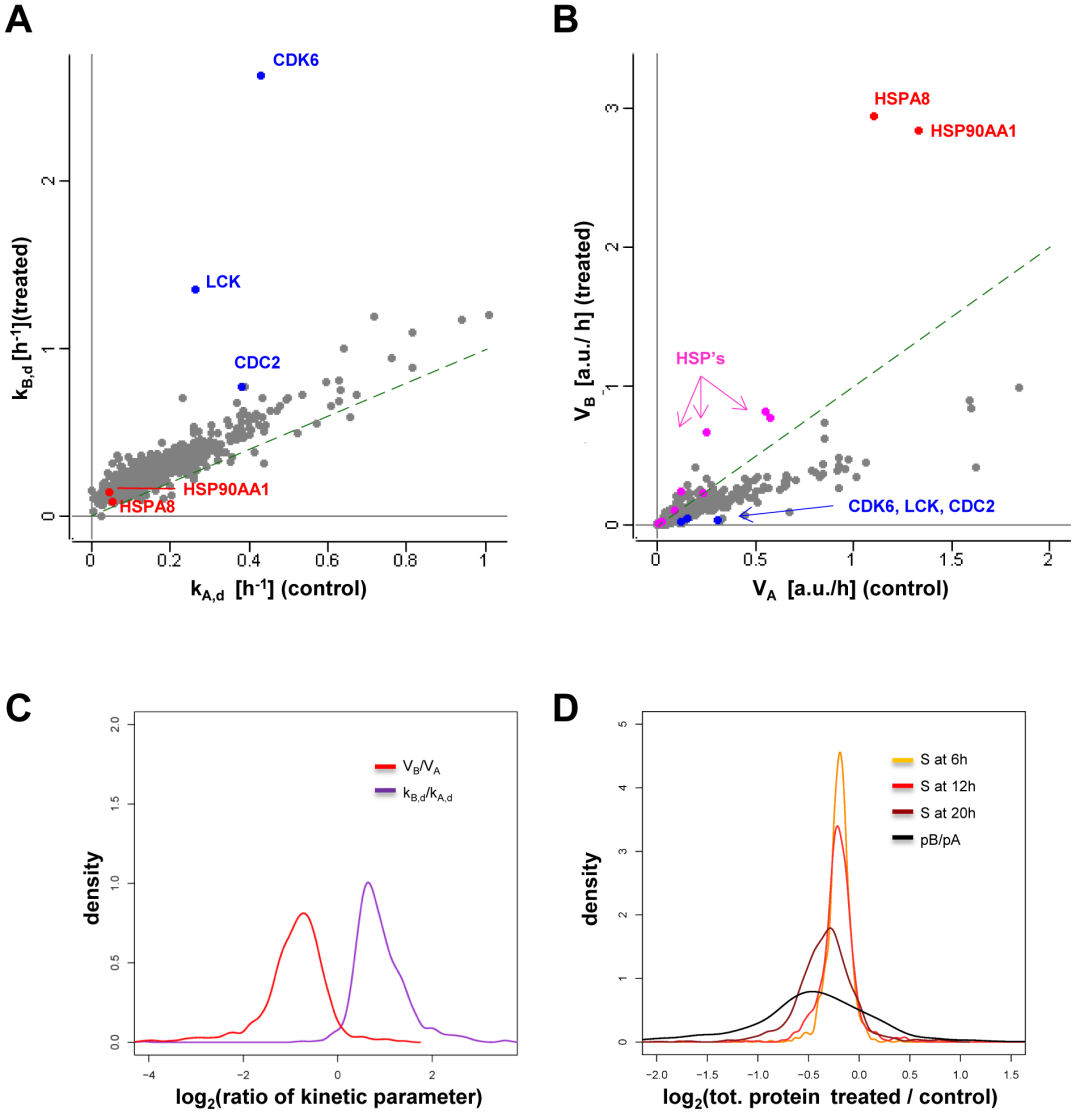
Results from calculations of decay constants, synthesis rates and evolution of total protein levels. Indexes of variables are A (as in k_A_, V_A_) for control (+DMSO) and B (as in k_B_, V_B_) for treated (+GA) cells. **A**) Scatter plot of the values of the degradation constants for the control and treated sample (experiment 2, 911 proteins). The position of reference proteins is indicated. The dashed line indicates a 1:1 relationship **B**) Scatter plot of V_A_ and V_B_ (same dataset as A). Other heat shock proteins are shown in pink. The dashed line indicates a 1:1 relationship **C**) Kernel density estimate of log_2_ of ratios of synthesis rates (V_B_/V_A_) and degradation constants (k_B,d_/k_A,d_) after correction for cell growth. **D**) Comparison of distributions of log_2_ of ratios *S* of net protein levels (treated/control) calculated from pcSILAC data at t = 6, 12, 20h vs. ratios at steady-state (t = infinite) calculated from the model. *S* values were corrected for mixing inequalities.

Only protein groups with complete data, i.e. 6 ratios at 3 time points, were used for calculations of kinetic parameters. Results for experiment 2 (911 proteins) are presented here. Data for experiment 1 (520 proteins) were overall very similar (Fig.S11-S13). Plots of decay rate constants and relative synthesis rates in the two conditions (Figure 4A, B) showed that the reference proteins CDK6, LCK, CDC2, HSP90AA1, HSPA8 (HSC70) displayed the expected patterns, with significant increases in the decay rate constants for the three kinases and increases in relative synthesis rates upon drug treatment for the two considered chaperones. In addition to showing some of the fastest decays in absolute terms, CDK6, LCK, and CDC2 also displayed decreased synthesis rates. Only a few meaningful comparisons can be done with published data, since most determinations of kinetic parameters so far have been purely qualitative or done in different conditions. Yorgin et al. [57] reported that treatment with 1.7μM GA increased approximately 2-fold the decay rate of mature LCK over a period of 24h. We detected an increase of a factor 5 in decay rate for pre-existing LCK.

### Decay rate constants at steady state: distribution and correlation with other studies

The determination of absolute values for decay rate constants in control cells (k_A_,_d_) allowed us to carry out comparisons of protein half-lives for steady-state cells with values reported previously [32], [35], [58]. After matching corresponding proteins by gene name, half-lives (T_1/2_) of protein groups from experiment 2 showed no significant correlation with the three published datasets, with Pearson’s *r* values between 0.014 and 0.065 (Fig. S14). It should be noted that cell types and organisms used in these studies were very different. In spite of this lack of direct correlation the overall distribution of half-lives in our data was similar to that measured by two of the other studies (figure S14), with similar medians (55.8 h in our data vs. 47.3 h for Wu et al. [32] and 47.8 h for Schwanhäusser et al.[58]). Annotation enrichment analysis performed on k_A,d_ values indicated that proteins involved in cell cycle and transcription as well as nuclear, DNA-binding and signaling proteins had higher than average degradation constants, i.e. high turnover (unstable proteins) (Table S5). Conversely, proteins participating in core metabolic pathways such as glycolysis, glucose catabolism, amine and alcohol metabolism had globally low k_d_’s (stable proteins). Heat shock proteins and Hsp90 cofactors, i.e. proteins that coadjuvate Hsp90 in the binding and folding of substrates, too, were very stable, long-lived proteins. Results from annotation enrichment analysis were thus qualitatively in good agreement with previously reported ones [58], and this despite the general lack of correlation at the individual protein level. This suggests that globally protein stability is a conserved feature, at least at the level of protein families.

### Hsp90 inhibition causes a global increase in decay which also affects chaperones and Hsp90 cofactors

We next analyzed the changes induced by drug treatment on decay rate constants k_d_’s. The median of the GA/DMSO ratio of the apparent decay rate constants k_B_app_/k_A_app_ was 0.75, but after correction for cell growth, decay appeared to be globally higher in drug treated cells with a median for k_B,d_/k_A,d_ of 1.74 (1.55 in exp.1) (Figure 4C). Nevertheless, results of annotation enrichment analysis for decay in treated cells (k_B,d_) were overall similar to those for control cells (k_A,d_). This was in line with the overall distribution of k_B,d_ values (Figure 4A) which shows that, relative to the overall population, the drug caused strong changes in decay for only a few proteins. Interestingly, two categories that were found to have increased degradation upon drug treatment were those of Hsp90 cofactors (defined as in www.picard.ch/downloads) and generally heat shock proteins (defined as described [43]), although within these categories the behavior varied significantly from protein to protein.

### Hsp90 inhibition results in a global shutdown of protein synthesis, while stress proteins are strongly induced

Analysis of the ratios of synthesis rate V_B_/V_A_ (GA/DMSO) showed that globally, synthesis rates were clearly decreased in drug-treated cells, with a median value for V_B_/V_A_ of 0.58 (Figure 4B,C) (0.55 in exp.1). Annotation enrichment analysis for the parameter V_B_/V_A_ highlighted the increased synthesis of stress-related polypeptides, i.e. chaperones, folding proteins and Hsp90 cofactors (Table S5). Other terms with increased synthesis were ER proteins, components of the N-linked glycosylation machinery, cytoplasmic vesicle proteins and subunits of the proteasome. Among proteins with decreased synthesis, the terms enriched comprised ribosomal and rRNA processing proteins as well as components of the purine and pyrimidine metabolic pathways.

### The theoretical new steady state is not reached in the course of the experiment

With a shift of the apex towards negative values, the distributions of net protein ratios (Figure 4D) illustrate the general evolution of the system, which sees the decrease of many proteins likely compensated by the increase in concentrations of a few abundant proteins, namely chaperones and stress proteins. From the decay constants k_A,d_, k_B,d_ and the synthesis rates V_B_/V_A_ values we derived the steady-state ratio of net protein concentrations p_B_/p_A_, which corresponds to the expected state of the system at the end of its evolution (with *t*  =  infinite). p_B_/p_A_ showed a good but not extremely high correlation (Pearson *r*  =  0.709) with the calculated net protein ratio at 20h obtained as the *S* ratio. A comparison of the distribution of net protein ratios calculated at the three time points with the expected final steady state concentrations p_B_/p_A_ shows the increasing divergence of the control and treated samples in time, but also suggests that at *t*  = 20h the system has not reached the steady state predicted by the model (Figure 4D). Whether the cells, given more time, would actually reach this theoretical new steady state is unclear. The evolution of ratios for some early induced proteins (e.g. DNAJB1 = Hsp40) (Figure 2C) could suggest that the GA-treated cells at t = 20h have started at least a partial recovery towards the basal state.

### Proteins and protein families show distinctive changes in decay and synthesis rates

**Figure 5:**
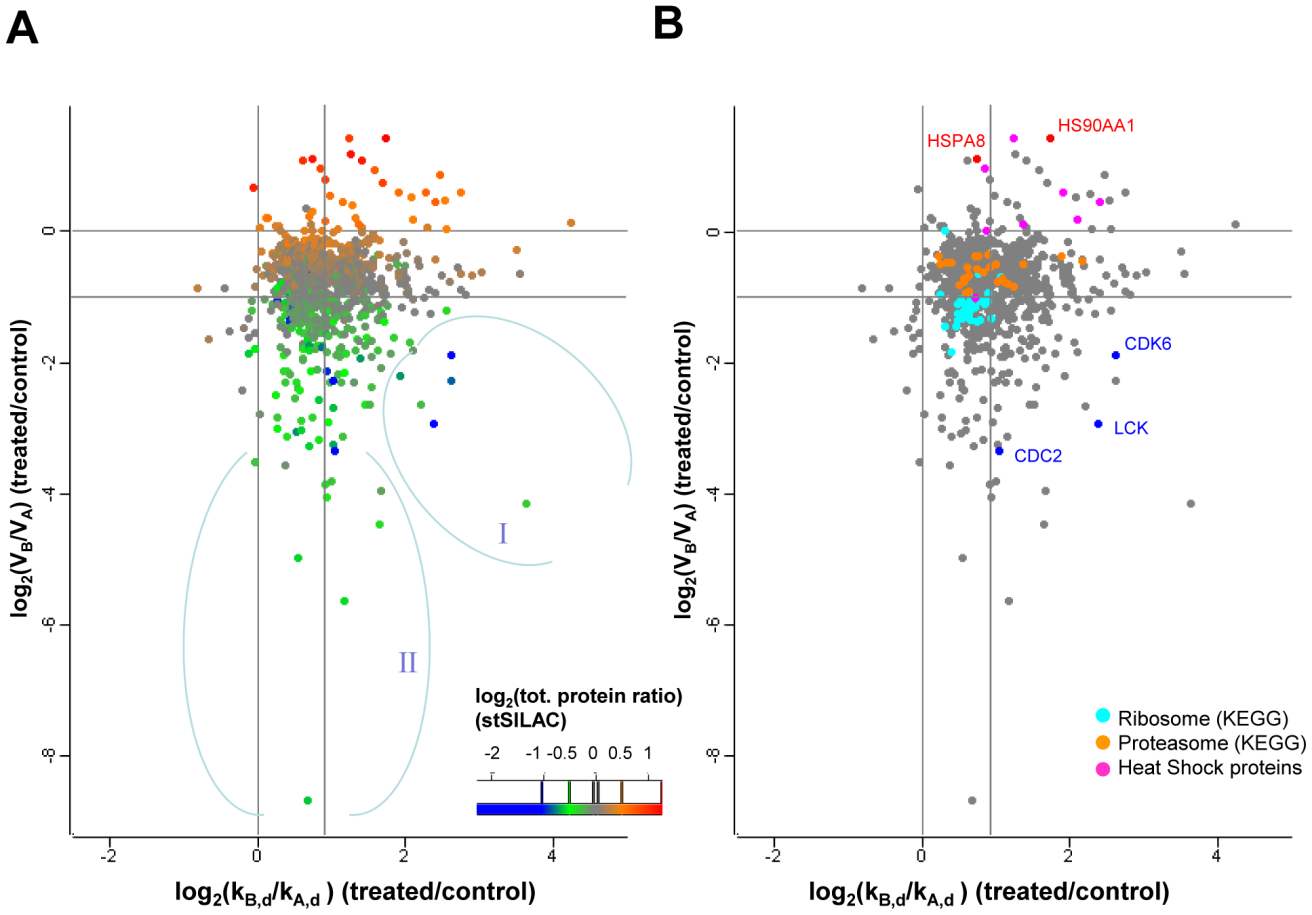
Changes in degradation and synthesis rates induced by Hsp90 inhibition and relationship with changes in net total protein levels and protein families. Indexes of variables are A (as in k_A_, V_A_) for control (+DMSO) and B (as in k_B_, V_B_) for treated (+GA) cells. A) Scatter plot of the values of ratios of intrinsic degradation constants k_B,d_/k_A,d_ vs. the ratios of synthesis rates V_B_/V_A._ The median values of k_B,d_/k_A,d_ and V_B_/V_A_ for the population are indicated with dashed lines. Coloring of points is according to the calculated treated /control total protein ratio at t = 20h (ratio *S*). B) Same as A) but with coloring of ribosomal, proteasome and heat shock proteins. Groups I and II are discussed in the main text. All values are log_2_.

A global representation of the observed changes in kinetic parameters (Figure 5A) shows how changes in decay rate constant (k_,d_) and synthesis rates (V_B_/V_A)_ combined to determine net protein changes at 20h (color gradient). Among proteins that show a net decrease in abundance, two groups seem to appear. The first one (I) includes CDK6 and LCK and is characterized by a strong increase in decay accompanied by a decrease in synthesis. A second group (II) shows a decrease in synthesis with little or no change in decay rates. The model predicts that the levels of group II protein would be expected to further decrease strongly at later time points. Whether this actually happened, would remain to be established. We explored the changes in decay and synthesis parameters for some protein families relevant to cellular proteostasis (Figure 5B). Ribosomal proteins had synthesis rates that decreased more than the general population, although they also appeared to be somewhat stabilized in the presence of GA. The overall result was a net decrease (median of log_2_(S)  =  -0.18 at 20h) and a decreased turnover. Subunits of the proteasome were, in relative terms, more synthesized in GA-treated cells, with no visible overall trend at the level of decay. The result was an average mild increase in their net levels (median of log_2_(S)  =  +0.104 at 20h) compared to untreated cells. The changes in net levels for these two categories of proteins were confirmed by standard SILAC analysis (Fierro-Monti et al., accompanying manuscript) and correlated well with the global decrease in protein synthesis and increase in degradation observed. Figure 5B also shows that heat shock proteins were among the most increasingly synthesized molecules in GA-treated cells, but intriguingly their decay rate ratios k_B_/k_A_ were often above average, indicating specifically increased turnover. Protein kinases constitute the most prominent class of known Hsp90 clients. Only 19 protein kinases were fully quantified in our pcSILAC experiments, most probably due to their low levels of expression and the limited proteome coverage obtained. Out of these, 6 (LCK, CDC2, CDK6, STK39, ZAP70, CSK) clearly showed increased decay or and/or decreased synthesis, while the remaining 13 kinases behaved roughly like the bulk of the proteome. Although the number of kinases quantitated by pcSILAC is low, the trend is in agreement with recent data suggesting that only about one third of the kinome decreases upon Hsp90 inhibition [32] or again that only about one third of all kinases binds with high affinity to Hsp90 [60]. A more detailed analysis of the patterns of changes observed for various families and functional classes of proteins is presented elsewhere (Fierro-Monti et al., accompanying manuscript).

### Changes in synthesis rates play a bigger role than changes in decay in shaping the proteome after perturbation

**Figure 6:**
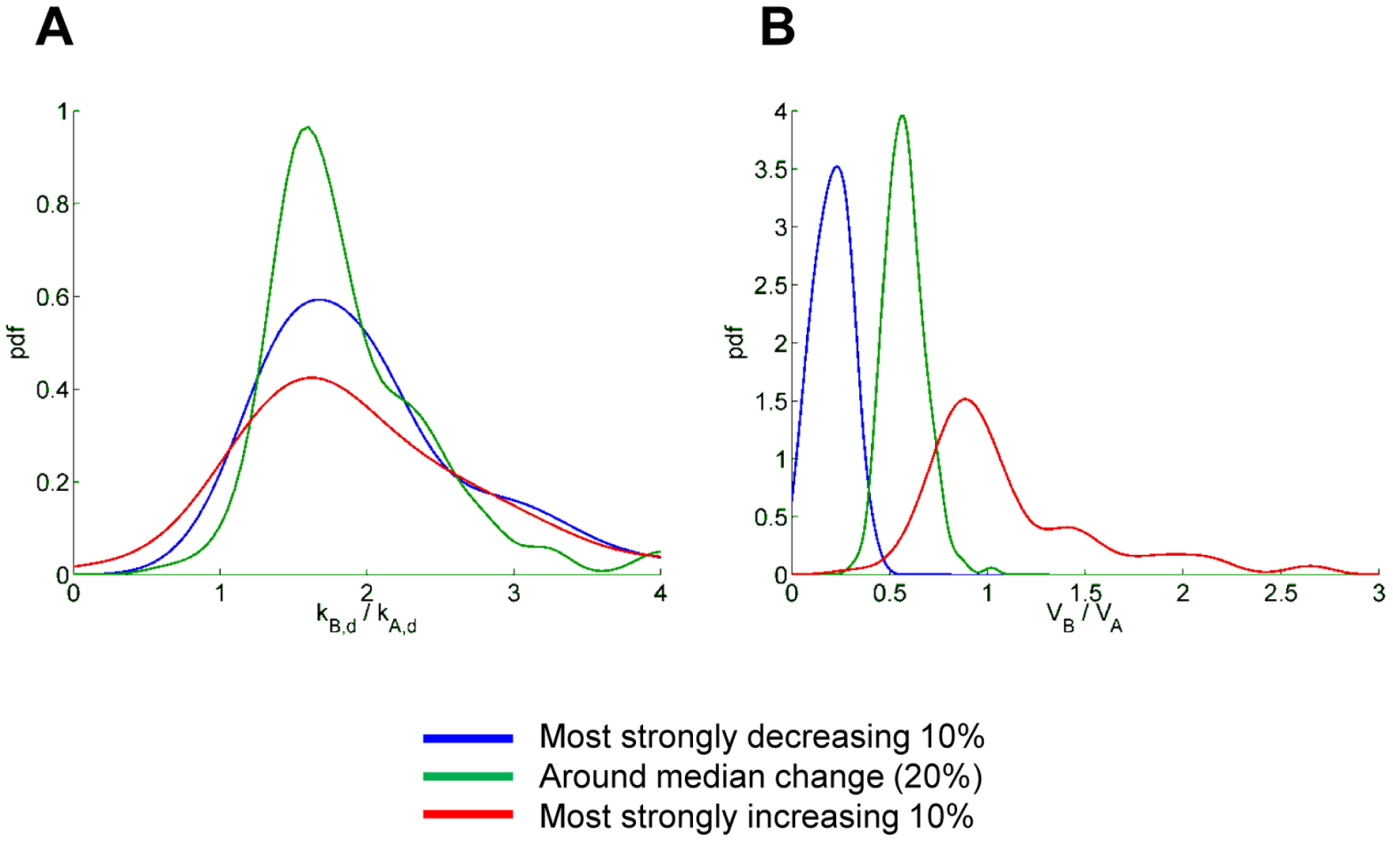
Changes in degradation and synthesis rates in subsets of net decreasing, invariant and increasing proteins. Indexes of variables are A (as in k_A_, V_A_) for control (+DMSO) and B (as in k_A_, V_A_) for treated (+GA) cells. Probability density functions of decay constant ratios k_B,d_/k_A,d_ (A) and synthesis rate ratios V_B_/V_A._ (B) are shown for the 10% most strongly increased proteins, the 10% most strongly decreased proteins and a 20% of proteins which were around the median of the concentration change (invariant proteins). The ratio of protein levels at steady-state (p_B_/p_A_ parameter) was used for the selection.

We then tried to ascertain the overall contribution of protein stability, its changes compared with changes in synthesis rates, to variations in net protein levels. Decay rate constants in control cells (k_A,d_) had a moderate inverse correlation with changes in net protein levels (*r*  =  –0.21 for exp.1, against stSILAC ratios from internal control at 20h) (Fig. S15), suggesting that intrinsically unstable proteins had a certain tendency to be depleted more than stable ones upon Hsp90 inhibition. This finding is in agreement to what was reported previously for protein kinases [32]. However, changes in protein synthesis induced by GA, i.e. V_B_/V_A_, correlated much stronger than decay rates k_A,d_ with changes in net protein levels (*r*  =  0.82 for exp.1, against internal stSILAC values, Fig. S15). Density plots of changes in decay and synthesis k_B_/k_A_ and V_B_/V_A_ (Figure 6A, B) for sets of decreasing, increasing and invariant proteins at t = 20h confirmed that overall net changes in abundance correlated strongly with changes in synthesis (V_B_/V_A_ values) and only weakly with changes in decay (k_B,d_/k_A,d_). To better quantitate the relative contributions of variations in synthesis and decay, we calculated total changes in fluxes (ΔW) of synthesis and decay (see supplementary information S1). Fluxes give a measure of actual, instantaneous “flow” of molecules, taking also protein concentration into account. The global values obtained for the whole population early during drug treatment were ΔW_synthesis_  =  –44.0 [a.u. x h^−1^] and ΔW_degradation_ =  –24.8 [a.u. x h^−1^]. The bigger absolute value of ΔW_synthesis_ further supported the view that, although the changes due to variations in decay are not negligible, those due to changes in synthesis are quantitatively more important. More specifically, it indicates that even for proteins that decrease in net concentration, their decrease is (on average) due more to a decrease in synthesis than to an increase in decay.

## Discussion

Quantitative proteomics techniques measure differences in net protein levels in complex mixtures such as whole cell extracts. Interpretation of datasets derived from such experiments is challenging, not the least because the underlying mechanisms that direct the changes in protein concentration are not immediately apparent from the final net changes. We developed pcSILAC in an attempt to shed light on the variations in synthesis and decay rates that determine final protein levels in a perturbed cellular system. We aimed at comparing two conditions directly in the same experiment and this posed major challenges for the design of the labeling scheme. A minimum of four channels was needed to measure two biological conditions each with the two labels (before/after medium exchange) corresponding to the pulse and chase experiments. Standard SILAC is limited to three channels and is thus insufficient. Multiplexing beyond three is difficult and risky, considering that triplex labeling already results in sometimes very complex, overlapping isotope patterns. We chose to separate the quantitation obtained with the two SILAC amino acids, to gain additional measurement channels. This allowed us to design a labeling scheme in which two biological samples were directly compared to each other in the MS measurements.

Boisvert et al. [35] developed and used a different strategy for measuring synthesis and degradation in a steady-state system, which uses a reference sample corresponding to the initial proteome against which the pulsed/chased signals are compared in a standard triplex SILAC configuration. The approach of Boisvert et al. could be expanded to measure two biological conditions separately in two distinct experiments. The disadvantage is that this would require doubling the already considerable necessary instrument time and performing the comparison in an indirect manner. Another labeling strategy has been reported recently in a study focusing on the effects of inhibitors of the mTOR complexes [61]. In this study however, the determination of decay and synthesis was based on separate experiments and the different kinetic parameters were not integrated in a comprehensive model. Also very recently, Troetschel et al. [62] proposed a different approach based on ^15^N and ^13^C for labeling of bacteria and were able to determine, with two different experiments, degradation and synthesis for a few hundreds of proteins in two conditions. Compared with both the schemes of Boisvert et al and Troetschel et al [62], the main advantage of pcSILAC is that it can measure both conditions together in the same experiment. The trade-off is that the proteome coverage that can be obtained by pcSILAC is, at parity of instrument time, reduced relative to simpler SILAC experiments because of the separation of quantitation in R and K channels and the exclusion of mixed-labeled peptides.

In pcSILAC, the first level of analysis is the direct comparison of the amounts of pre-existing and newly synthesized proteins through the (H/M)_R_ and (H/M)_K_ ratios. This was important to perform as a first level of evaluation and verification on raw data upstream of the complex mathematical data processing. It also allowed a preliminary qualitative analysis of relatively large numbers of proteins at single time points, without the restrictions imposed by the kinetic parameter calculations which require having data for three time points per each protein. pcSILAC data however contain more information than just the (H/M)_R_ and (H/M)_K_ ratios. For example, the possibility to easily calculate pseudo-stSILAC ratios (*S*) from pcSILAC data was crucial for a quality control of the experiment and for establishing correlations between kinetic parameters and changes in net protein levels. Also, thanks to the partial redundancy of the data, robust fittings were obtained when all 6 ratios were used for obtaining k_d_ and V values, likely because possible inaccuracies in one value were compensated by other ratios available.

Compared to stSILAC experiments, pcSILAC can provide several unique levels of information. First, the possibility to distinguish newly synthesized from pre-existing proteins and to compare these two pools between the two samples gives the possibility to detect changes in global protein metabolism. In the model system of Hsp90 inhibition, pcSILAC revealed an important reduction of the global protein synthesis rate, not unexpected in stressed cells. Such reduction in synthesis is associated with (and may be one of the causes of) the observed cell cycle arrest. Simultaneously, the observed increase in overall degradation could stem from defects in the folding machinery. Similar changes in synthesis and degradation are undetectable in standard quantitative proteomics experiments in which normalization by protein amount destroys the possibility to capture variations e.g. in total protein content of the cell or in general protein turnover.

Furthermore, the possibility to derive true decay rate constants k_d_ for both the control and treated cells allowed a real evaluation of intrinsic protein stability in both control and treated cells, while until now the determination of k_d_’s and half-lives was limited to steady-state cells. For the system under study, the determination of synthesis rate ratios V_B_/V_A_, together with the knowledge of decay rate constants (k_d_’s), allowed a comprehensive assessment of the factors that determine net protein changes. Compared with the raw (H/M)_R_, (H/M)_K_ ratios, decay rate constants k_d_ and synthesis rate ratios V_B_/V_A_ have an improved information content since the synthesis and decay terms can be separated and analyzed individually. For example, it was possible to establish that CDK6 is among all the quantitated proteins, the one with the largest absolute decrease in stability upon Hsp90 inhibition, but that its apparent synthesis rate is also diminished, although less than other proteins. Conversely, numerous other polypeptides are only affected at the level of synthesis (group II in Figure 5A), whereas their stability is almost unchanged. Indeed, the results from pcSILAC would suggest that, apart from a general moderate increase in decay, changes in stability following Hsp90 inhibition affect only a limited subset of the proteome, while the largest net changes in protein levels can be ascribed to modulation at the level of synthesis. A very important caveat is, however, that the model is assuming that one single pool of each protein exists in the cell, which decays and is synthesized in a homogeneous fashion. Paradoxically, Hsp90 inhibition may be one case in which this assumption only partially holds true. It has indeed been shown [39], [63] that many Hsp90 “clients” that are depleted in the presence of GA are indeed being synthesized but are immediately targeted for proteasomal degradation likely because their folding cannot be completed. Such a pool of protein would not be subjected to the same decay rate as the bulk of pre-existing (and presumably mostly correctly folded) protein and would appear in pcSILAC as reduced synthesis rather than increased decay. It is therefore likely that the synthesis rate (V) values measured by pcSILAC for a number of direct Hsp90 clients are distorted by co-translational degradation. A further dissection of translation and early post-translational events would require use of protein synthesis- or proteasome inhibitors, which however would result themselves in major perturbations of proteostasis, raising further questions on the relevance of the data obtained. An exciting option for future studies would be the measurements of ubiquitination levels [64], whereby it must be kept in mind that ubiquitination does not always result in proteasomal degradation. Moreover, the assumption that each protein behaves as a homogeneous cellular pool is clearly an oversimplification, especially for eukaryotic cells. More in-depth analyses by pcSILAC should therefore aim at separating and analyzing subcellular compartments as previously done for steady-state cells [65], [66].

Technically, the pcSILAC strategy could be improved at several levels. Measurements of samples at more than three time points could significantly improve the fitting of the kinetic parameters. The number of samples that can be analysed is, however, limited by the mass spectrometer time needed, which can be quite important for complex proteomes. Another critical point of pcSILAC – which is common to any approach for the determination of kinetic constants – is the need to measure accurately cell growth in the two biological conditions. The correction for growth does indeed have a major impact on the absolute values of decay rate constants (k_d_’s) and inaccuracies in cell counting can result in systematic biases in the final data. Ideally, to reflect faithfully the cellular phenotype, sample loading should be normalized by cell number instead of protein amount, and counting of cells should be done in parallel with flow cytometry analysis to detect possible changes in size and shape of cells upon treatment. It would then be possible to integrate e.g. cell volume parameters in the mathematical model to provide more accurate and representative results.

Last but not least, the model described here assumes that protein synthesis and decay rates undergo a change at *t = t_d_* and then remain constant thereafter, converging towards a new steady-state. Although in our data most proteins displayed an evolution consistent with such a simple model, a few proteins had a more complex behavior that could not be fitted properly, with e.g. a quick increase in synthesis followed by a slowdown after 6h (DNAJB1 in Figure 2C and others in figure S16). It is also conceivable that other proteins could increase their synthesis only after a lag time due to gene activation events. It remains to be tested if such cases require a separate modeling or can be integrated, through measurements of more time points, into a single multiphasic model. A proper assessment of the observed proteome changes would also need the monitoring of the specificity of the Hsp90 inhibitor and of its stability both in the medium and intracellularly during the duration of the experiment, all aspects that have been often neglected in similar studies. Finally, the full characterization of the system by pcSILAC was so far possible for less than 1000 proteins, likely the most abundant ones in the cell. A deeper exploration of changing proteomes – which will require improvements in the technology and the workflow - may reveal a larger variety of possible responses to a given perturbation.

## Supporting Information

Figure S1 **Samples and conditions of standard and pcSILAC experiments**. Experimental design for pcSILAC experiments; the yellow arrow indicates treatment with geldanamycin (GA) or DMSO. Correspondingly numbered replicates were mixed (e.g. M1+H1, M2+H2) after total protein quantification. For replicates M1 and H1 the treatment was inverted, i.e. H1 received DMSO while M1 was treated with GA. **A**) Experimental design for pcSILAC experiment 1; coloured triangles indicate medium exchange. The samples used as an internal standard SILAC experiment in pcSILAC experiment 1 were derived from the same culture used for pcSILAC labeling, therefore no medium exchange was done and quantitation was performed only on R (H/M). **B**) Same as A) for pcSILAC experiment 2 (no internal standard SILAC replicate was done in this experiment).
(TIF)

Figure S2 **Predicted and possible isotope peaks for various classes of tryptic peptides in pcSILAC**. **A**) Isotope labeled amino acids used in the study and their monoisotopic mass shifts relative to light amino acids **B**) General Labeling scheme for pcSILAC with description of the isotopomers present in the media before/after medium exchange (represented as a coloured triangle) at t = 0 **C**) Possible isotope peaks of 1 x R- or 1 x K-containing peptides after mixing H+L pcSILAC samples, with expected mass shifts relative to a R0, resp. K0 peptide. **D**) Same as C) but for peptides containing 2 K residues. The occurrence of mixed-label peptides (*) during the phase of medium exchange was considered. **E**) Same as D) but for peptides containing 2x R residues. **F**) Same as D) but for peptides containing **both** 1 K and 1 R.
(TIF)

Figure S3 **Differences in ratios measured for R- vs. K-containing peptides**. « Medium- » and « heavy » extracts from cells at t = 6, 12 and 20h from pcSILAC experiments before mixing were separated by SDS-PAGE. Fractions in the range 30–150 kDa (A,B) or 60-100 kDa (C,D) were digested and analysed by MS. For every protein, ratios were determined separately based on K and R peptides. Data for a set of high-scoring proteins is represented as scatter plots (one point per protein, non-log ratios). All values plotted correspond to (new label)/(old label) ratios. (**A**) DMSO-treated cells (M, L labeled) from pcSILAC experiment 1 (200 proteins) at 12h (**B**) Geldanamycin-treated cells (H,L labelled) from pcSILAC experiment 1 at 12h (200 proteins). (**C**)**-**DMSO-treated control cells (M, L labelled) from pcSILAC experiment 2 (80 proteins) (**D**) same as (C) but for GA-treated cells (H,L labeled)**.**
(TIF)

Figure S4 **Peptides containing both R and K in pcSILAC**. **A**) Isotope pattern observed for peptide **GHYTEGAELVDSVLDVVRK** (TBB2A_HUMAN) at t = 6h in the H+L mix of pcSILAC experiment 1, replicate 2. The position of possible peaks resulting from label mixing (old label + new label) are indicated below the axis, with their expected mass shifts (see figure S1) **B**) Same as A) but for peptide **GVAINMVTEEDKR** (IF4A1_HUMAN) at t = 12h in pcSILAC experiment 1, replicate 1. Overall, the predicted isotope patterns for K+R-peptides were observed. Peaks resulting from label mixing (old label + new label) were sometimes detectable but had very low intensity (<3% of base peak, marked below x-axis) compared to homogeneously (old+old, new+new) labeled species. Spectra obtained for samples before mixing were also inspected with similar results.
(TIF)

Figure S5 **Isotope patterns observed for KK- or RR-containing peptides in pcSILAC samples before mixing (experiment 1, t = 12h).** **A**) Isotope pattern observed for peptide KVESLQEEIAFLK (MH+ = 767.429, 2+, VIME_HUMAN) in the "light" sample of pcSILAC experiment 1, replicate 2 (+DMSO). Location of possible isotope peaks resulting from label mixing (old+new label) are indicated together with the expected mass shift relative to the fully "light" peak. **B**) Peptide IINEPTAAAIAYGLDKK (MH+ = 894.4986,2+, HSP7C_HUMAN), "heavy" sample, replicate 2, (+GA). **C**) Peptide ARFEELNADLFR (740.88112, 2+, HSP7C_HUMAN), "light sample, replicate 2, (+DMSO) **D**) Peptide NLDIERPTYTNLNR (580.304783, 3+, TBA1B_HUMAN), "heavy" sample, replicate 2 (+GA). Intensity of the mixed-labeled peptide peaks remained below 5% of that of the newly synthesized peptide peak.
(TIF)

Figure S6 **Correlation between pcSILAC datasets (t = 20h) of different experiments.** Scatter plots and Pearson’s *r* correlation coefficients for two pcSILAC experiments, each with two biological replicates. After MaxQuant co-analysis, and processing for K,R quantitation, datasets were filtered for a minimum evidence count  = 3. All ratios were normalized and log2. (**A**) (H/M)_R_ (**B**) (H/M)_K_ (**C**) Same as (A),(B) but replicates for each experiment were averaged before plotting and calculating inter-experiment correlations. (**D**) (H/L)_R_ ratios for the same replicates, showing that R-based ratios, too can highly correlated between replicates and experiments. **Note**: the low values of the r coefficient obtained for (H/M)_R_ can be explained with the very small spread of the (H/M)_R_ values, i.e. the fact that most values are very close to 1 and therefore the correlation is strongly influenced by noise, which tends to be constant and random.
(TIF)

Figure S7 **Reproducibility of values of (H/M)_K_,(H/M)_R_ ratios for reference proteins and evolution in time in two pcSILAC experiments.** Normalized ratios are shown. **A**)**-C**) pcSILAC experiment 1, **D**)**-F**) pcSILAC experiment 2. DNAJB1  =  Hsp40; HSP90AA1  =  Hsp90alpha; HSP90AB1 = Hsp90beta; PTGES3 = p23; PRAKAR1A  =  regulatory subunit of Protein kinase A; POLR2A  =  DNA-directed RNA polymerase II subunit RPB1; EEF2 = Elongation factor 2; CDC2 = Cyclin-dependent kinase 1; LCK  =  tyrosine protein kinase LCK; CDK6 = cyclin-dependent kinase 6.
(TIF)

Figure S8 **Global distribution of (H/M)_K_, (H/M)_R_ ratios measured in pcSILAC experiments 1 and 2**. Boxplots of global (H/M)_K_, (H/M)_R_ ratios for replicate 1 of pcSILAC experiment 1, (yellow), replicate 1 of experiment 2 (blue), replicate 2 of experiment 2 (green), replicate 2 of experiment 2 (pink). “R” stands for (H/M)_R_, “K” for (H/M)_K_. **A**)**, C**)**, E**)**, G**) show values after correction for mixing ratio only. **B**)**, D**)**, F**)**, H**) show values after correction for mixing ratio and normalization. Ratios were inverted whenever necessary to facilitate comparison between replicates with inverse treatment.
(TIF)

Figure S9 **(H/M)_K_, (H/M)_R_ ratios from pcSILAC experiment 1 (t = 20h) and their relationship with stSILAC (net protein) values**. The values of (H/M)_K_, (H/M)_R_ (median of two replicates ) at t = 20h are represented as scatter plot, with color coding according to the medians at t = 20h obtained for the same protein from an internal (carried out simultaneously, with the same cells ) stSILAC experiment. A degree of correlation is observed, stronger between (H/M)_K_ and standard SILAC values. However, similar values in std SILAC experiments can correspond to different combinations of (H/M)_K_, (H/M)_R_ values.
(TIF)

Figure S10 **Calculation of a net protein ratio S from pcSILAC data and correlation with net protein ratios determined experimentally through independent experiments. A**) Correlation between theoretical S_1_ and S_2_ values (from pcSILAC data) obtained with two complementary equations (eq.5 and eq.6 in main manuscript) using different ratios. Data were from replicate 2 of experiment 1, not corrected for mixing ratio inequalities. **B**) Correlation of S (calculated as average of S_1_, S_2_, normalized) with experimental standard SILAC (net protein) ratios from a parallel experiment performed simultaneously (« internal » control) or **C**) an independent standard SILAC experiment performed 6 weeks earlier. Log_2_ have been applied on median-centered values of ratios. Data are from pcSILAC experiment 1.
(TIF)

Figure S11 **Results from calculations of of kinetic parameters for pcSILAC experiment 1 (same plots as in main** **Figures 4**–**5** **, other experiment)**. **A**) Scatter plot of decay rate constants for the control and treated sample (experiment 1, 520 proteins). The position of reference proteins is indicated. **B**) Scatter plot of Vs_control and Vs_treated in the same dataset. Other heat shock proteins are shown in pink **C**) Scatter plot of the values of ratios of intrinsic degradation constants k_B,d_/k_A,d_ vs. the ratios of synthesis rates V_B_/V_A_. The median values of k_B,d_/k_A,d_ and V_B_/V_A_ for the population are indicated with dashed lines. Coloring of points indicates ribosomal, proteasome and heat shock proteins.
(TIF)

Figure S12 **Comparing the fitting and the resulting k_d,app_ and V_B_/V_A_ ratios from two pcSILAC experiments**. Values of kinetic parameters for 462 proteins fully quantified in both pcSILAC experiments are shown, together with the value of the residual norm of the error (sum parameter ResNorm for both experiments). ResNorm gives a measure of the quality of the fit between experimental data and the model. Proteins with lower summed ResNorm, indicating a good fit, are usually closer to the diagonal than the ones with higher values of ResNorm.
(TIF)

Figure S13 **Comparing the fitting and the resulting steady state values of protein concentrations in control and treated samples from two pcSILAC experiments**. **A**) Values of predicted steady-state concentrations for 462 proteins fully quantified in both pcSILAC experiments are shown, color-coded by the value of the residual norm of the error (resNorm parameter, sum for both experiments). ResNorm gives a measure of the quality of the fit between experimental data and the model. Proteins with lower summed resNorm, indicating a good fit, are usually closer to the diagonal than the ones with higher values of resNorm. **B**) is an enlargment of the plot in A).
(TIF)

Figure S14 **Comparison of half-lives obtained by pcSILAC with published datasets**. Half-lives were retrieved from the supplementary data of three published works and are compared with data from pcSILAC experiment 2 (untreated cells). Protein groups were matched by gene name. The data were acquired on the following cell lines; Wu et al: CAL27, oral adenosquamous carcinoma; Schwanhäusser et al: mouse NIH 3T3 fibroblasts; Boisvert et al: HeLa cells (whole cell half-life dataset). **A**) Boxplot of the four datasets (outliers not shown) Note: the dataset of Boisvert et al contained a large number of stable proteins for which half-lives could not be calculated accurately and were given a value of 999. These were not used for this plot **B**) Kernel density estimate for three out of the four datasets **C**) Scatterplot and Pearson’s correlation coefficient of values from pcSILAC against those reported by Wu et al. 1. Boisvert, F.-M., Ahmad, Y., Gierlinski, M., Charrière, F., Lamont, D., Scott, M., Barton, G., et al. (2012). Molecular & cellular proteomics: MCP, 11(3). 2. Wu, Z., Moghaddas Gholami, A., & Kuster, B. (2012). Molecular & cellular proteomics: MCP, 11(6). 3. Schwanhäusser, B., Busse, D., Li, N., Dittmar, G., Schuchhardt, J., Wolf, J., Chen, W., et al. (2011). Nature, 473(7347), 337–42.
(TIF)

Figure S15 **Correlation between synthesis rates, decay rate constants and changes in net protein levels in the two pcSILAC experiments**. Pearson’s *r* values are shown. Plots represent the datasets highlighted in yellow in the tables. **A**) Correlation of k_A_, k_B_ and log_2(_V_B_/V_A_) with the experimentally determined stSILAC ratio at 20h in experiment 1. **B**) Correlation of k_A_, k_B_ and log_2_(V_B_/V_A_) with computationally derived ratio of net protein levels in experiment 2 (20h).
(TIF)

Figure S16 **Example of proteins with poor fitting to the model**. Ratios (H/M)_K_, measuring newly synthesized protein levels from experiment 2 are shown. Values are after correction for mixing inequality and log2 transformation. Some proteins with apparent multiphasic behavior are shown in red, together with their value of the ResNorm parameter (Blue), which is a measure of the quality of the fitting. Most proteins highlighted here have values of ResNorm well above the median (0.18) of ResNorm for the whole population. It is to be noted however that, since the fitting is performed on 6 series of ratios, deviations due to noise in one series can be compensated by other values. DNAJB1 and GRP78 were quantitated with large numbers of peptides and thus their values should be reliable and reflect a complex temporal dynamic of changes.
(TIF)

Table S1 **Global statistics incl. numbers of quantified peptides for pcSILAC experiments 1 and 2, each with replicates 1 and 2 (.XLS).**
(XLS)

Table S2 **data for pcSILAC experiment 1 after MaxQuant processing (before model application and rates calculations).** Includes all ratios H/M,H/L,M/L for both K- and R-peptides at all time points and replicates (.XLS).
(XLSX)

Table S3 **Data for pcSILAC experiment 2 after MaxQuant processing (before model application and rates calculations).** Includes all ratios H/M,H/L,M/L for both K- and R-peptides at all time points and replicates (.XLS).
(XLSX)

Table S4 **Kinetic parameters calculated for each replicate of experiment 1 and experiment 2, including statistics (.XLS).**
(XLSX)

Table S5 **Gene Ontology annotation enrichment analysis performed on decay rates k_d_ and synthesis rates V for GA-treated and DMSO cells (.XLS).**
(XLSX)

Supplementary Information S1 **Main supporting information including i) detailed discussion of isotope patterns of various types of peptides ii) Description of the mathematical model and computational framework for pcSILAC including derivation of all kinetic parameters and fluxes iii) all supplementary figures S1-S16 in one file to facilitate viewing after download (PDF).**
(PDF)

File S1 Scripts (perl) for processing of MaxQuant output (first part of the pcSILAC data analysis pipeline) (txt document).
(TXT)
