# Supplementary figures and images for: A Novel Pulse-Chase SILAC Strategy Measures Changes in Protein Decay and Synthesis Rates Induced by Perturbation of Proteostasis with an Hsp90 Inhibitor
Source: PLoS One. 2013 Nov 27;8(11):e80423. doi: 10.1371/journal.pone.0080423 (PMC3842330; doi:10.1371/journal.pone.0080423)

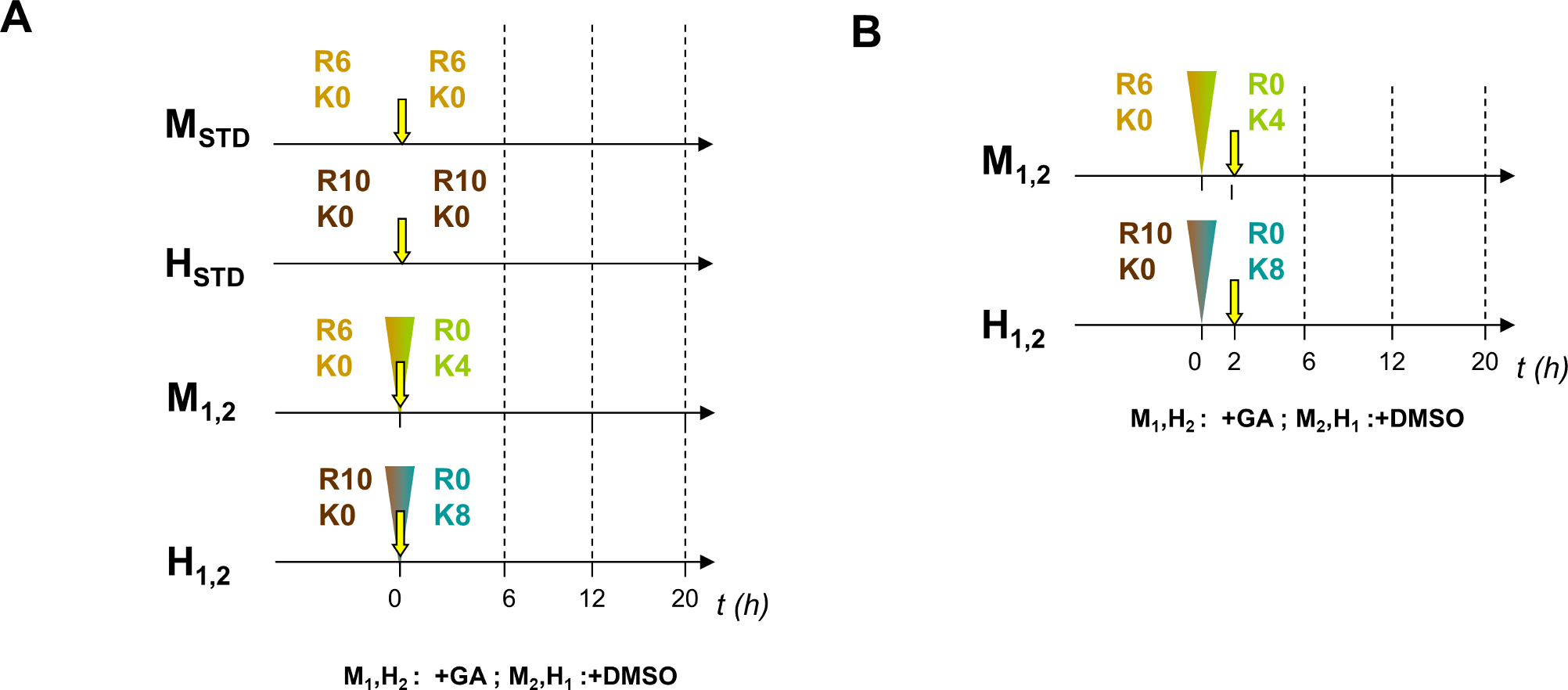

Supplement: Figure S1 — Samples and conditions of standard and pcSILAC experiments. Experimental design for pcSILAC experiments; the yellow arrow indicates treatment with geldanamycin (GA) or DMSO. Correspondingly numbered replicates were mixed (e.g. M1+H1, M2+H2) after total protein quantification. For replicates M1 and H1 the treatment was inverted, i.e. H1 received DMSO while M1 was treated with GA. A) Experimental design for pcSILAC experiment 1; coloured triangles indicate medium exchange. The samples used as an internal standard SILAC experiment in pcSILAC experiment 1 were derived from the same culture used for pcSILAC labeling, therefore no medium exchange was done and quantitation was performed only on R (H/M). B) Same as A) for pcSILAC experiment 2 (no internal standard SILAC replicate was done in this experiment). (TIF) [file pone.0080423.s001.tif]

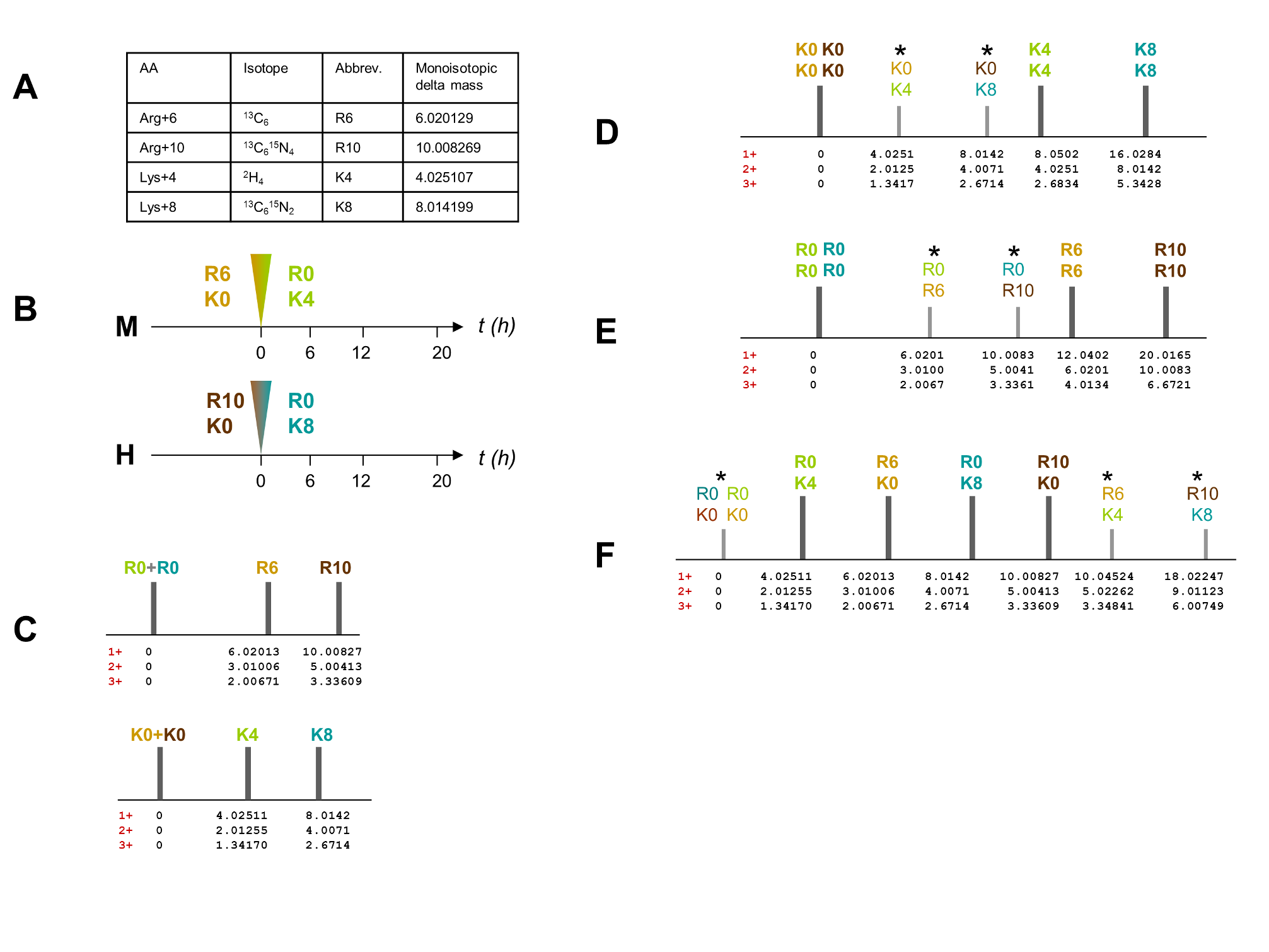

Supplement: Figure S2 — Predicted and possible isotope peaks for various classes of tryptic peptides in pcSILAC. A) Isotope labeled amino acids used in the study and their monoisotopic mass shifts relative to light amino acids B) General Labeling scheme for pcSILAC with description of the isotopomers present in the media before/after medium exchange (represented as a coloured triangle) at t = 0 C) Possible isotope peaks of 1 x R- or 1 x K-containing peptides after mixing H+L pcSILAC samples, with expected mass shifts relative to a R0, resp. K0 peptide. D) Same as C) but for peptides containing 2 K residues. The occurrence of mixed-label peptides (*) during the phase of medium exchange was considered. E) Same as D) but for peptides containing 2x R residues. F) Same as D) but for peptides containing both 1 K and 1 R. (TIF) [file pone.0080423.s002.tif]

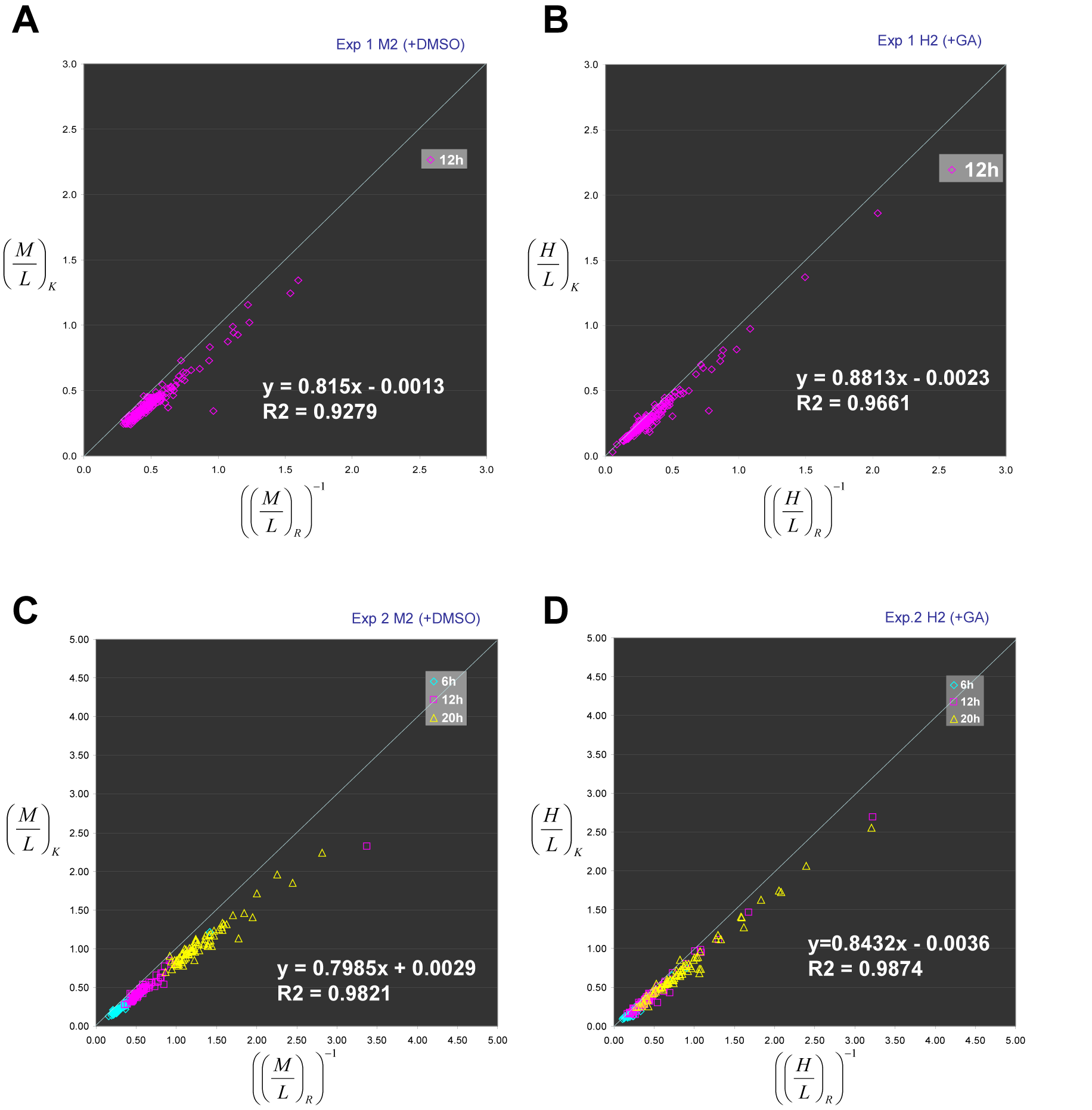

Supplement: Figure S3 — Differences in ratios measured for R- vs. K-containing peptides. « Medium- » and « heavy » extracts from cells at t = 6, 12 and 20h from pcSILAC experiments before mixing were separated by SDS-PAGE. Fractions in the range 30–150 kDa (A,B) or 60-100 kDa (C,D) were digested and analysed by MS. For every protein, ratios were determined separately based on K and R peptides. Data for a set of high-scoring proteins is represented as scatter plots (one point per protein, non-log ratios). All values plotted correspond to (new label)/(old label) ratios. (A) DMSO-treated cells (M, L labeled) from pcSILAC experiment 1 (200 proteins) at 12h (B) Geldanamycin-treated cells (H,L labelled) from pcSILAC experiment 1 at 12h (200 proteins). (C)-DMSO-treated control cells (M, L labelled) from pcSILAC experiment 2 (80 proteins) (D) same as (C) but for GA-treated cells (H,L labeled). (TIF) [file pone.0080423.s003.tif]

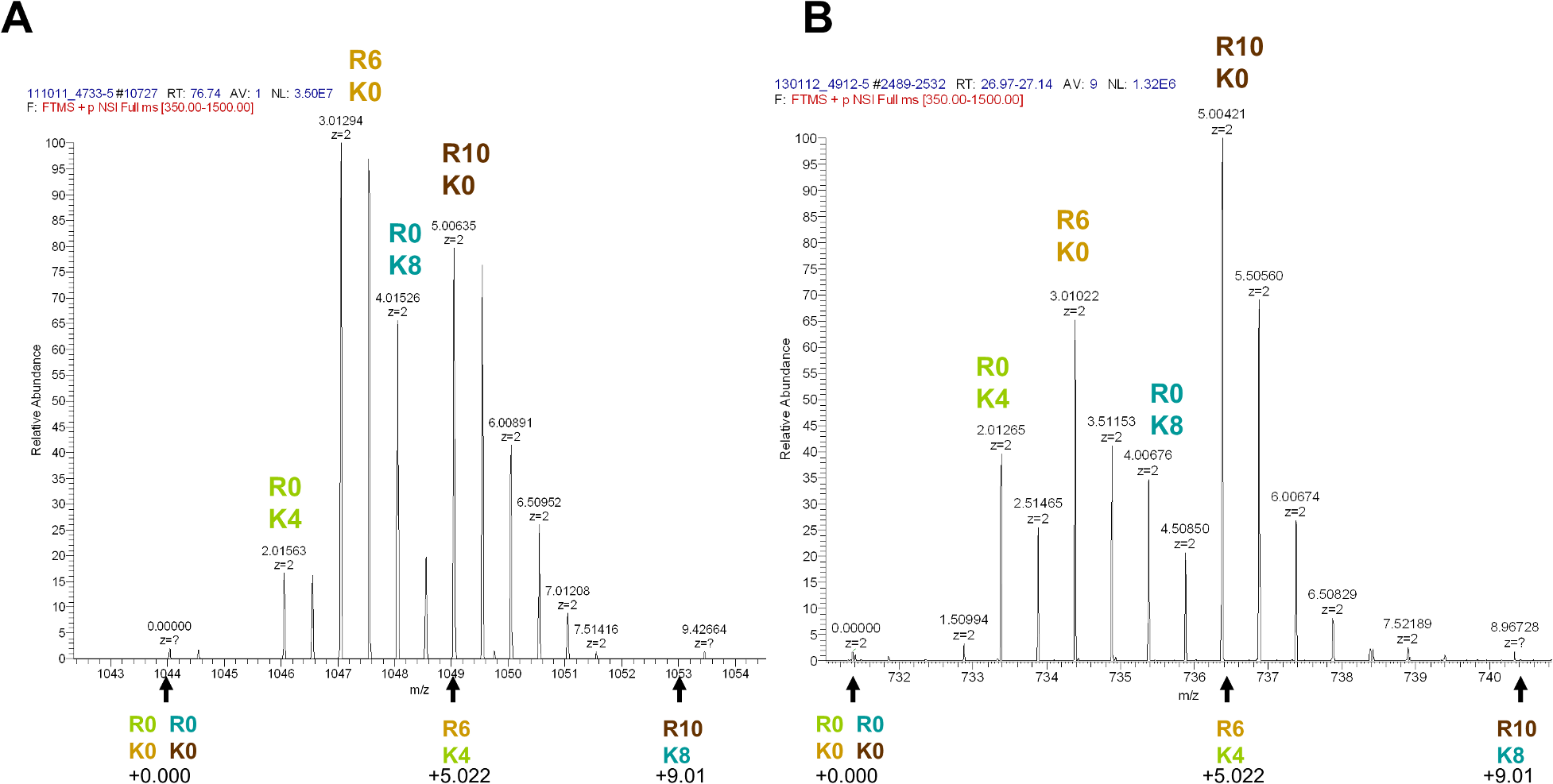

Supplement: Figure S4 — Peptides containing both R and K in pcSILAC. A) Isotope pattern observed for peptide GHYTEGAELVDSVLDVVRK (TBB2A_HUMAN) at t = 6h in the H+L mix of pcSILAC experiment 1, replicate 2. The position of possible peaks resulting from label mixing (old label + new label) are indicated below the axis, with their expected mass shifts (see figure S1) B) Same as A) but for peptide GVAINMVTEEDKR (IF4A1_HUMAN) at t = 12h in pcSILAC experiment 1, replicate 1. Overall, the predicted isotope patterns for K+R-peptides were observed. Peaks resulting from label mixing (old label + new label) were sometimes detectable but had very low intensity (<3% of base peak, marked below x-axis) compared to homogeneously (old+old, new+new) labeled species. Spectra obtained for samples before mixing were also inspected with similar results. (TIF) [file pone.0080423.s004.tif]

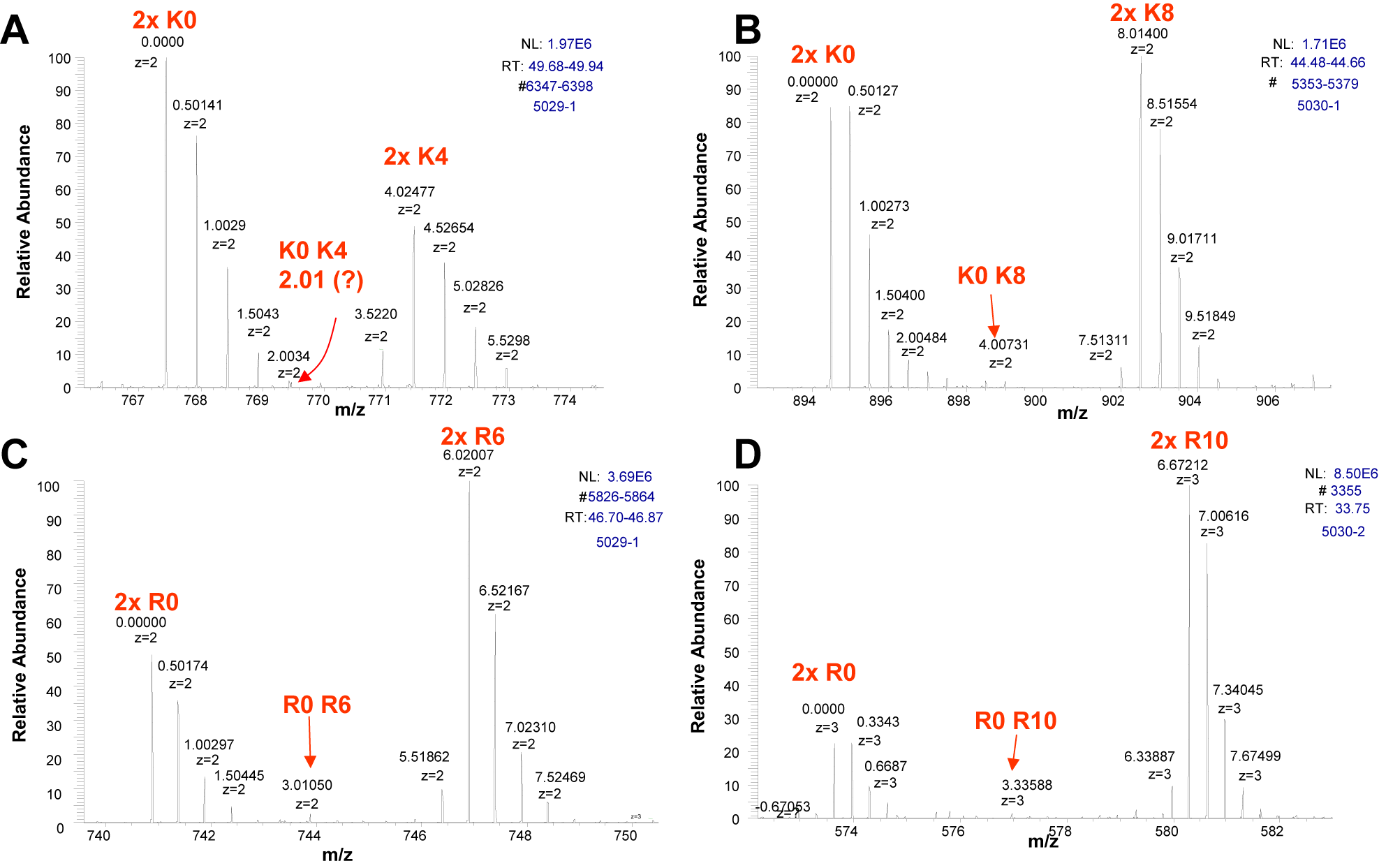

Supplement: Figure S5 — Isotope patterns observed for KK- or RR-containing peptides in pcSILAC samples before mixing (experiment 1, t = 12h). A) Isotope pattern observed for peptide KVESLQEEIAFLK (MH+ = 767.429, 2+, VIME_HUMAN) in the "light" sample of pcSILAC experiment 1, replicate 2 (+DMSO). Location of possible isotope peaks resulting from label mixing (old+new label) are indicated together with the expected mass shift relative to the fully "light" peak. B) Peptide IINEPTAAAIAYGLDKK (MH+ = 894.4986,2+, HSP7C_HUMAN), "heavy" sample, replicate 2, (+GA). C) Peptide ARFEELNADLFR (740.88112, 2+, HSP7C_HUMAN), "light sample, replicate 2, (+DMSO) D) Peptide NLDIERPTYTNLNR (580.304783, 3+, TBA1B_HUMAN), "heavy" sample, replicate 2 (+GA). Intensity of the mixed-labeled peptide peaks remained below 5% of that of the newly synthesized peptide peak. (TIF) [file pone.0080423.s005.tif]

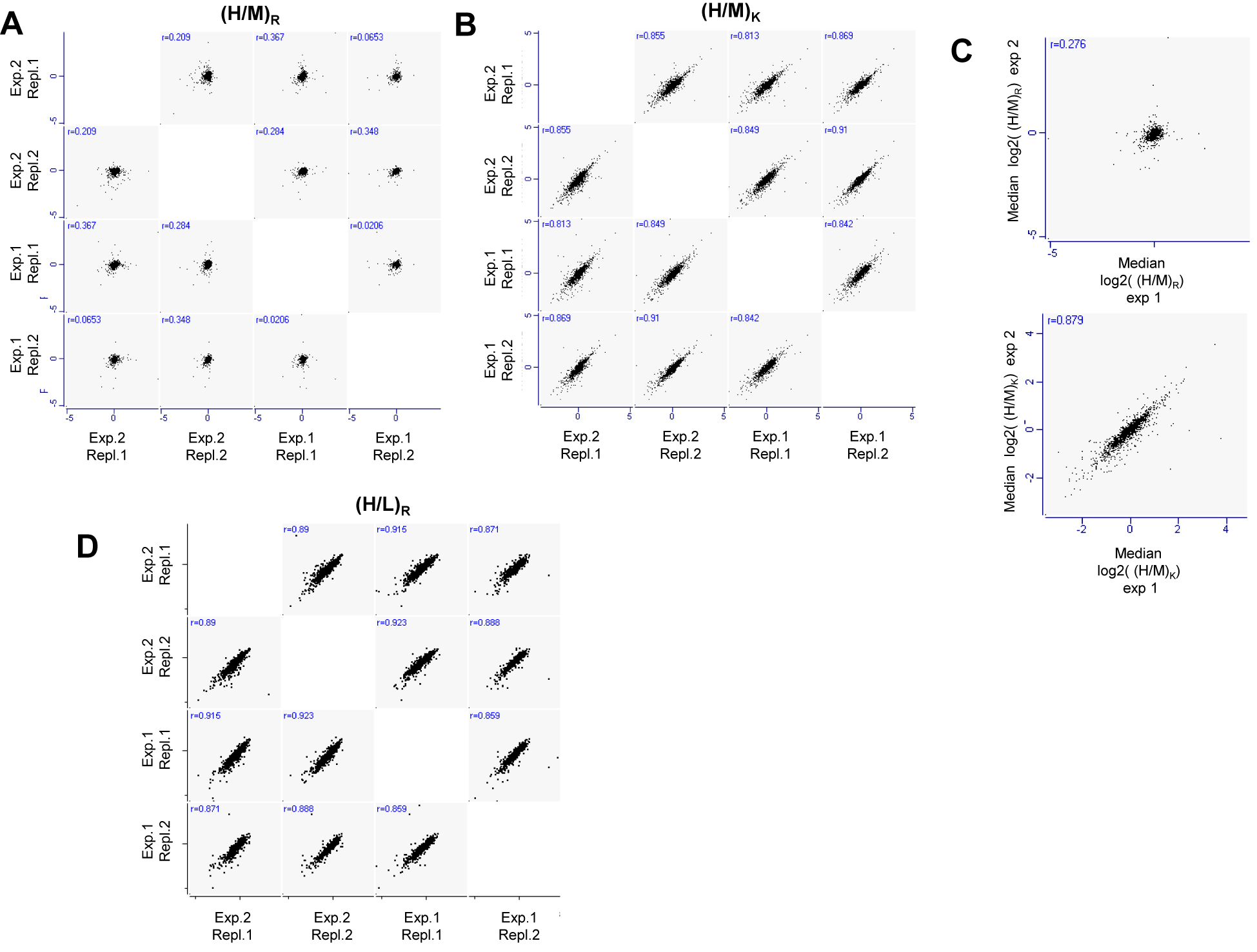

Supplement: Figure S6 — Correlation between pcSILAC datasets (t = 20h) of different experiments. Scatter plots and Pearson’s r correlation coefficients for two pcSILAC experiments, each with two biological replicates. After MaxQuant co-analysis, and processing for K,R quantitation, datasets were filtered for a minimum evidence count = 3. All ratios were normalized and log2. (A) (H/M)R (B) (H/M)K (C) Same as (A),(B) but replicates for each experiment were averaged before plotting and calculating inter-experiment correlations. (D) (H/L)R ratios for the same replicates, showing that R-based ratios, too can highly correlated between replicates and experiments. Note: the low values of the r coefficient obtained for (H/M)R can be explained with the very small spread of the (H/M)R values, i.e. the fact that most values are very close to 1 and therefore the correlation is strongly influenced by noise, which tends to be constant and random. (TIF) [file pone.0080423.s006.tif]

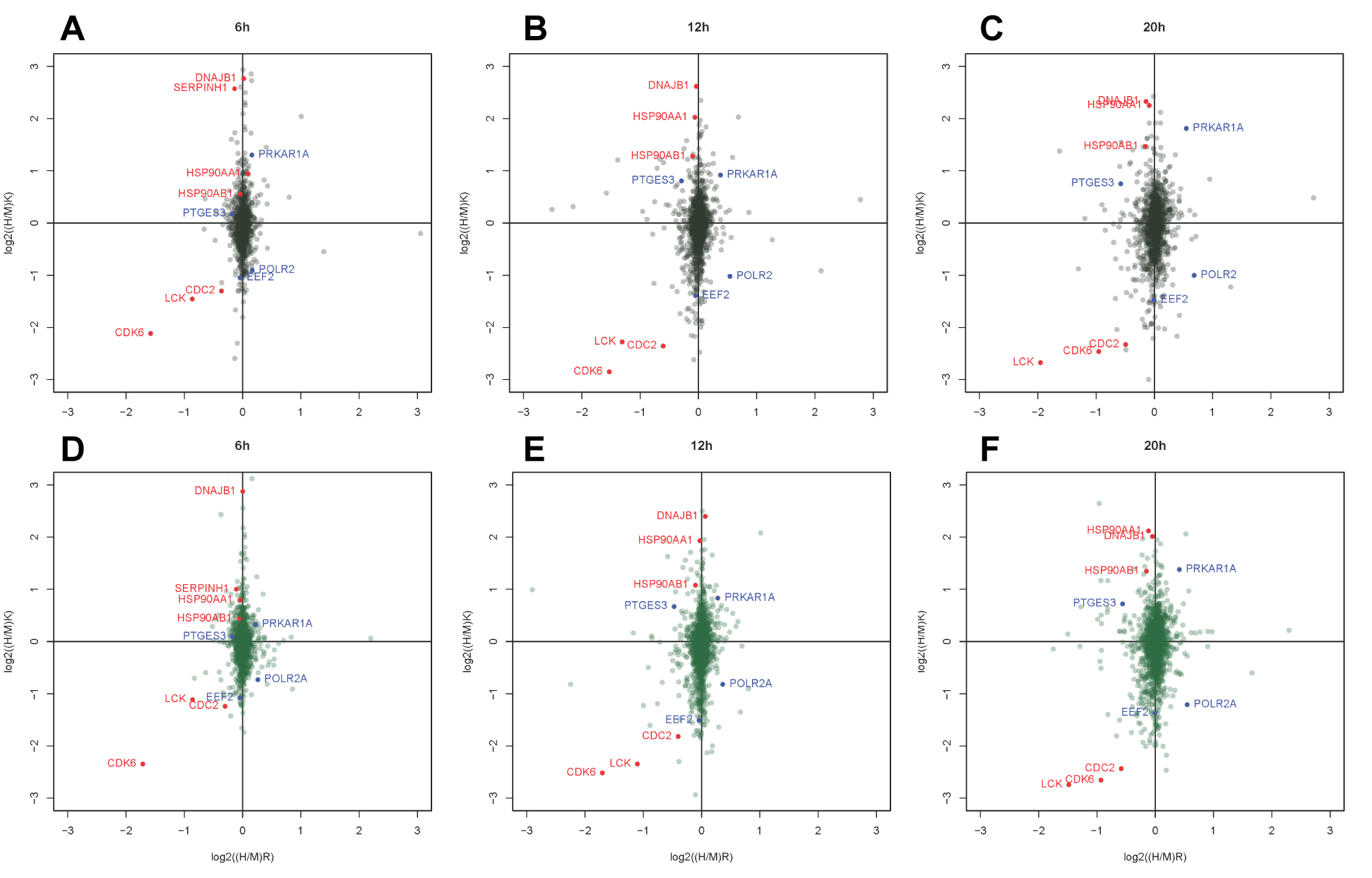

Supplement: Figure S7 — Reproducibility of values of (H/M)K,(H/M)R ratios for reference proteins and evolution in time in two pcSILAC experiments. Normalized ratios are shown. A)-C) pcSILAC experiment 1, D)-F) pcSILAC experiment 2. DNAJB1 = Hsp40; HSP90AA1 = Hsp90alpha; HSP90AB1 = Hsp90beta; PTGES3 = p23; PRAKAR1A = regulatory subunit of Protein kinase A; POLR2A = DNA-directed RNA polymerase II subunit RPB1; EEF2 = Elongation factor 2; CDC2 = Cyclin-dependent kinase 1; LCK = tyrosine protein kinase LCK; CDK6 = cyclin-dependent kinase 6. (TIF) [file pone.0080423.s007.tif]

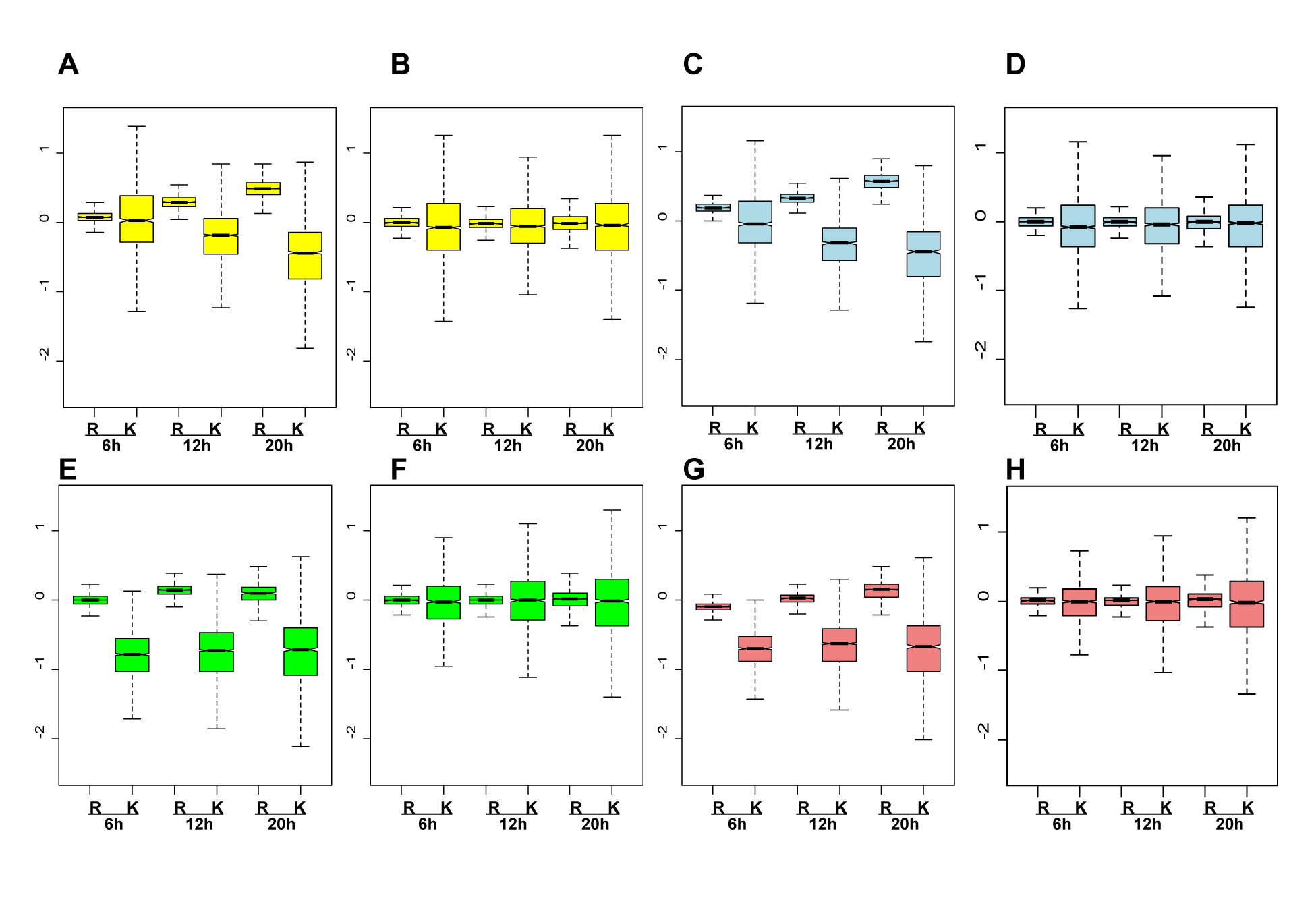

Supplement: Figure S8 — Global distribution of (H/M)K, (H/M)R ratios measured in pcSILAC experiments 1 and 2. Boxplots of global (H/M)K, (H/M)R ratios for replicate 1 of pcSILAC experiment 1, (yellow), replicate 1 of experiment 2 (blue), replicate 2 of experiment 2 (green), replicate 2 of experiment 2 (pink). “R” stands for (H/M)R, “K” for (H/M)K. A), C), E), G) show values after correction for mixing ratio only. B), D), F), H) show values after correction for mixing ratio and normalization. Ratios were inverted whenever necessary to facilitate comparison between replicates with inverse treatment. (TIF) [file pone.0080423.s008.tif]

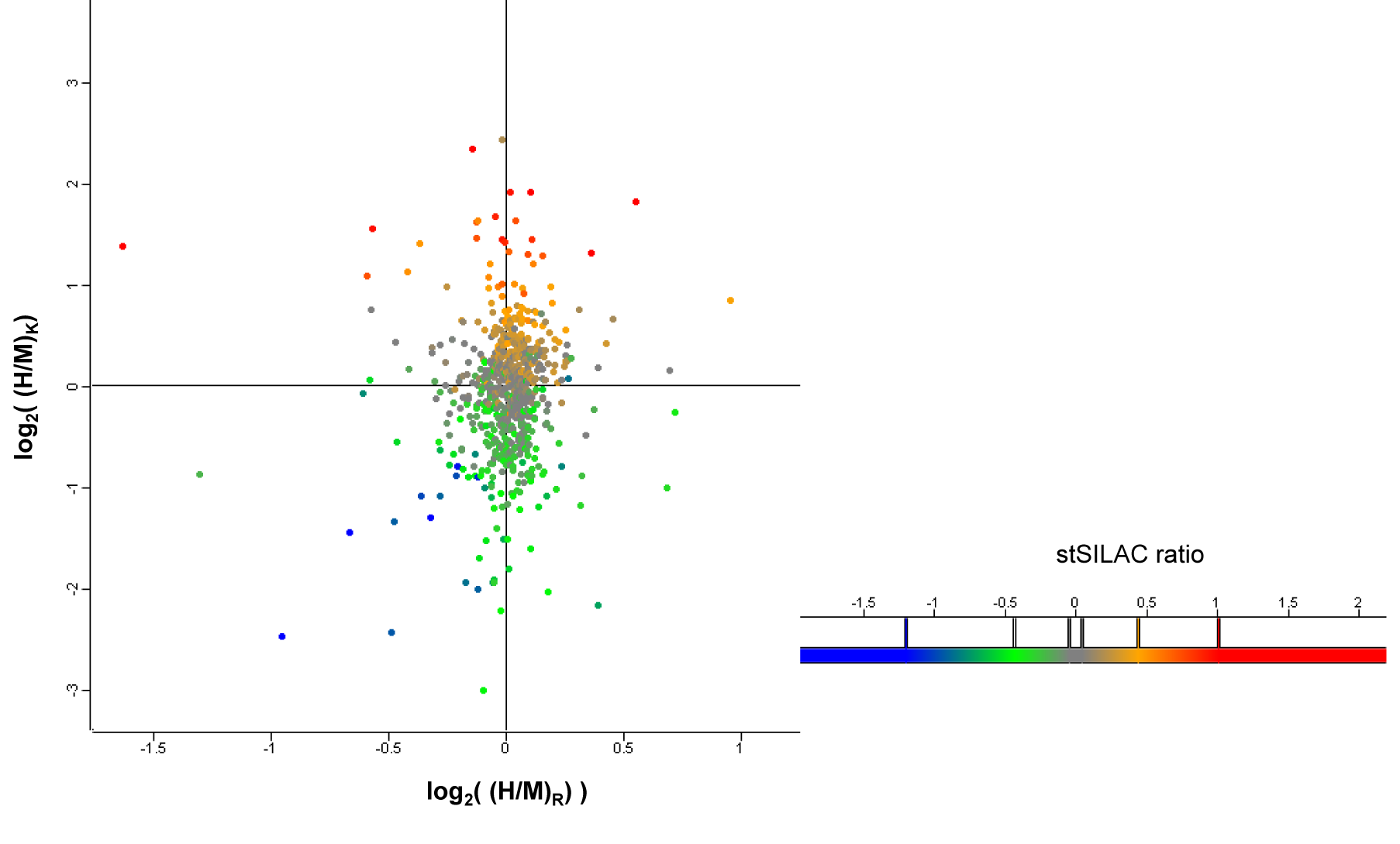

Supplement: Figure S9 — (H/M)K, (H/M)R ratios from pcSILAC experiment 1 (t = 20h) and their relationship with stSILAC (net protein) values. The values of (H/M)K, (H/M)R (median of two replicates ) at t = 20h are represented as scatter plot, with color coding according to the medians at t = 20h obtained for the same protein from an internal (carried out simultaneously, with the same cells ) stSILAC experiment. A degree of correlation is observed, stronger between (H/M)K and standard SILAC values. However, similar values in std SILAC experiments can correspond to different combinations of (H/M)K, (H/M)R values. (TIF) [file pone.0080423.s009.tif]

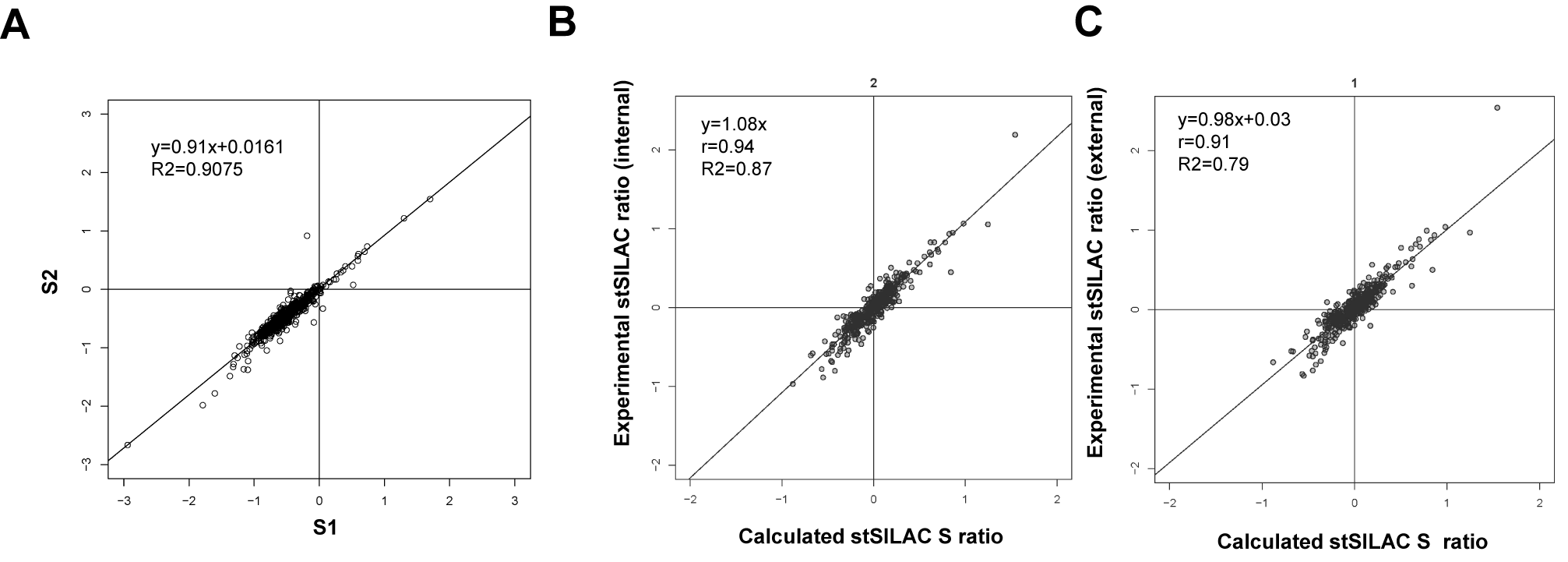

Supplement: Figure S10 — Calculation of a net protein ratio S from pcSILAC data and correlation with net protein ratios determined experimentally through independent experiments. A) Correlation between theoretical S1 and S2 values (from pcSILAC data) obtained with two complementary equations (eq.5 and eq.6 in main manuscript) using different ratios. Data were from replicate 2 of experiment 1, not corrected for mixing ratio inequalities. B) Correlation of S (calculated as average of S1, S2, normalized) with experimental standard SILAC (net protein) ratios from a parallel experiment performed simultaneously (« internal » control) or C) an independent standard SILAC experiment performed 6 weeks earlier. Log2 have been applied on median-centered values of ratios. Data are from pcSILAC experiment 1. (TIF) [file pone.0080423.s010.tif]

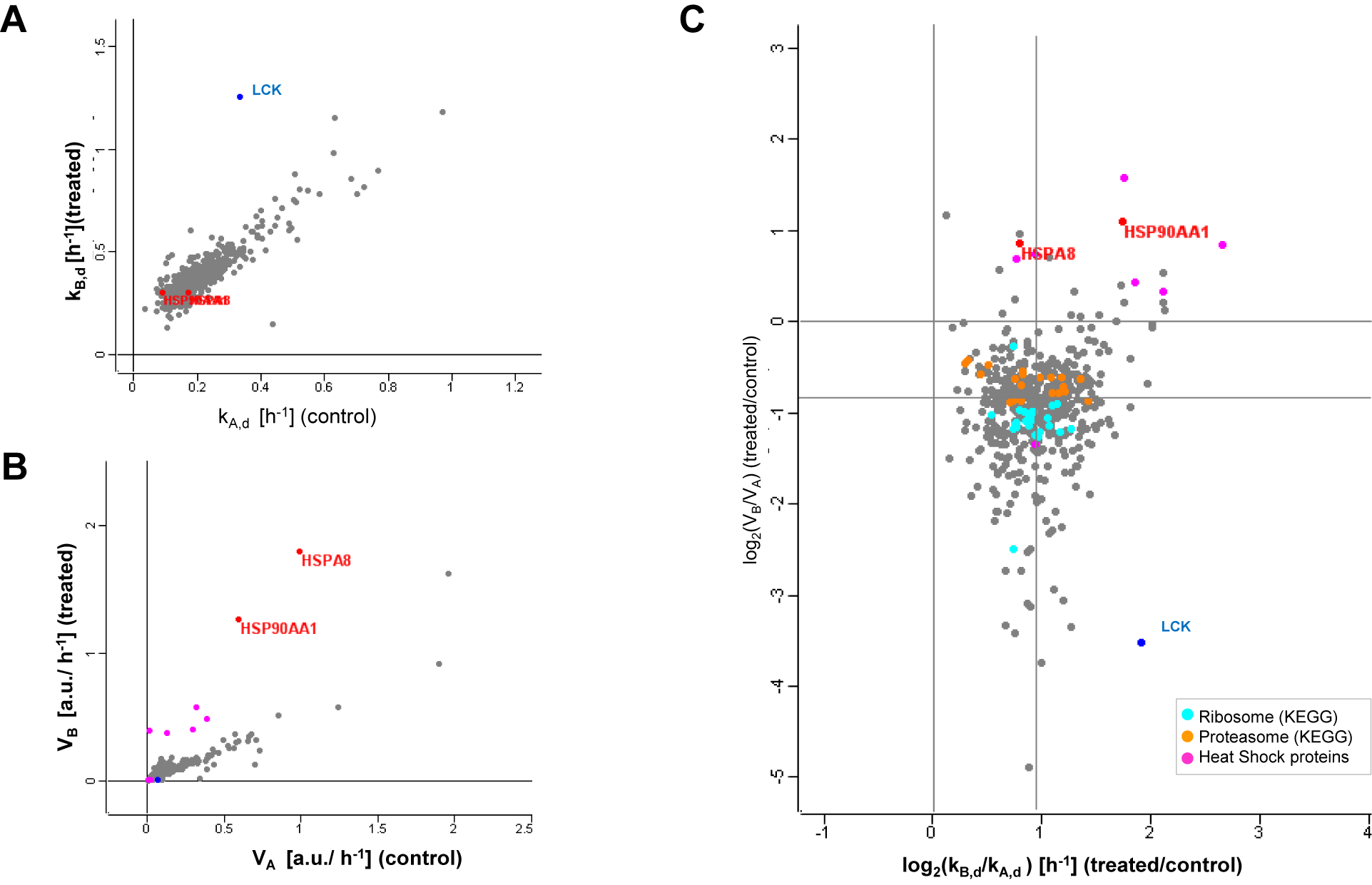

Supplement: Figure S11 — Results from calculations of of kinetic parameters for pcSILAC experiment 1 (same plots as in main Figures 4 – 5 , other experiment). A) Scatter plot of decay rate constants for the control and treated sample (experiment 1, 520 proteins). The position of reference proteins is indicated. B) Scatter plot of Vs_control and Vs_treated in the same dataset. Other heat shock proteins are shown in pink C) Scatter plot of the values of ratios of intrinsic degradation constants kB,d/kA,d vs. the ratios of synthesis rates VB/VA. The median values of kB,d/kA,d and VB/VA for the population are indicated with dashed lines. Coloring of points indicates ribosomal, proteasome and heat shock proteins. (TIF) [file pone.0080423.s011.tif]

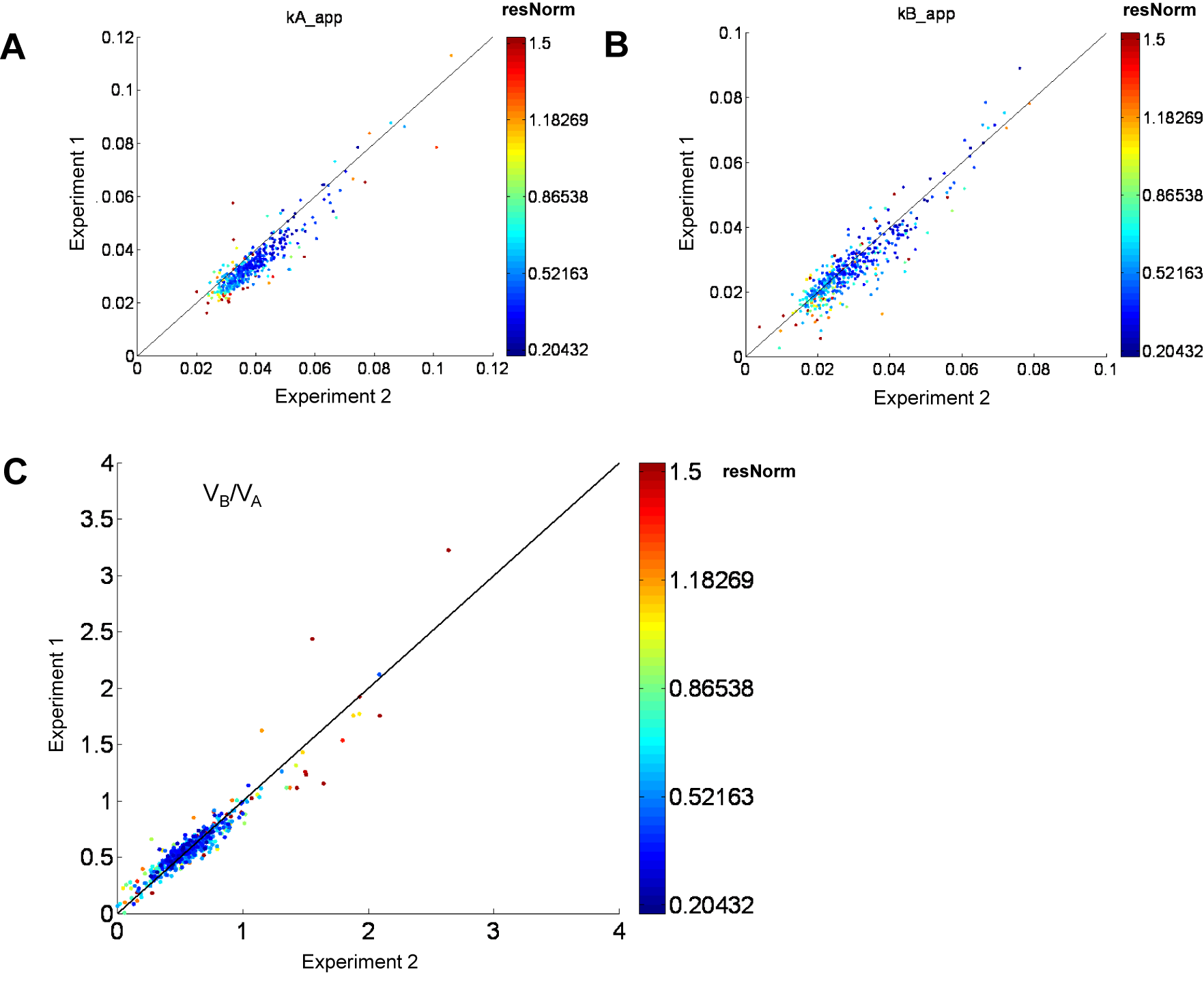

Supplement: Figure S12 — Comparing the fitting and the resulting kd,app and VB/VA ratios from two pcSILAC experiments. Values of kinetic parameters for 462 proteins fully quantified in both pcSILAC experiments are shown, together with the value of the residual norm of the error (sum parameter ResNorm for both experiments). ResNorm gives a measure of the quality of the fit between experimental data and the model. Proteins with lower summed ResNorm, indicating a good fit, are usually closer to the diagonal than the ones with higher values of ResNorm. (TIF) [file pone.0080423.s012.tif]

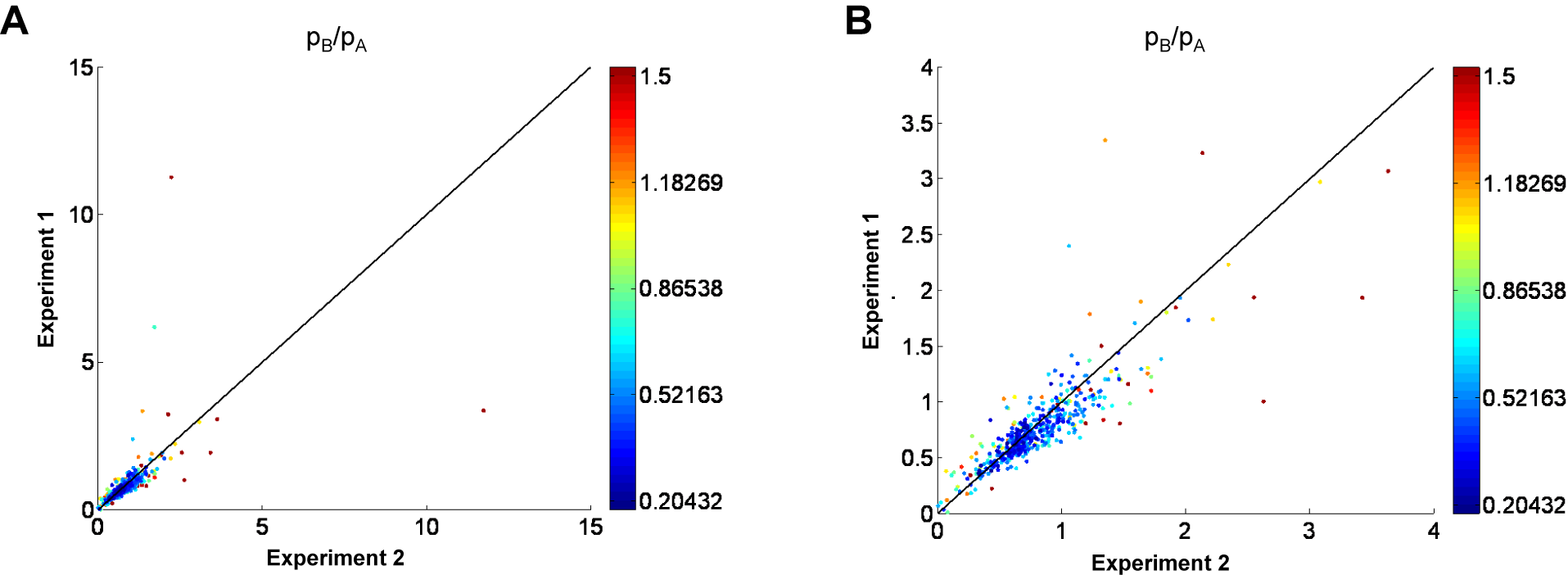

Supplement: Figure S13 — Comparing the fitting and the resulting steady state values of protein concentrations in control and treated samples from two pcSILAC experiments. A) Values of predicted steady-state concentrations for 462 proteins fully quantified in both pcSILAC experiments are shown, color-coded by the value of the residual norm of the error (resNorm parameter, sum for both experiments). ResNorm gives a measure of the quality of the fit between experimental data and the model. Proteins with lower summed resNorm, indicating a good fit, are usually closer to the diagonal than the ones with higher values of resNorm. B) is an enlargment of the plot in A). (TIF) [file pone.0080423.s013.tif]

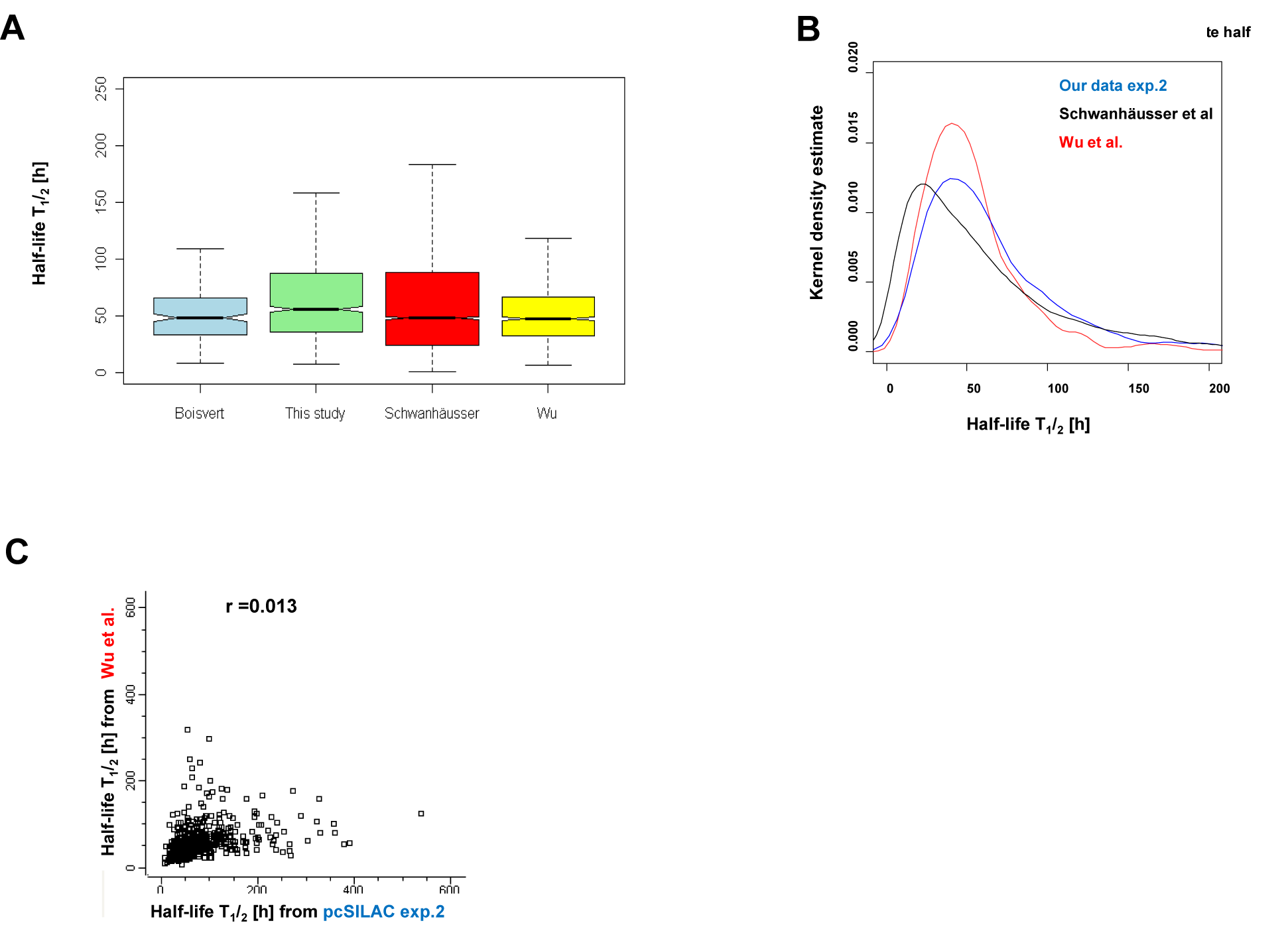

Supplement: Figure S14 — Comparison of half-lives obtained by pcSILAC with published datasets. Half-lives were retrieved from the supplementary data of three published works and are compared with data from pcSILAC experiment 2 (untreated cells). Protein groups were matched by gene name. The data were acquired on the following cell lines; Wu et al: CAL27, oral adenosquamous carcinoma; Schwanhäusser et al: mouse NIH 3T3 fibroblasts; Boisvert et al: HeLa cells (whole cell half-life dataset). A) Boxplot of the four datasets (outliers not shown) Note: the dataset of Boisvert et al contained a large number of stable proteins for which half-lives could not be calculated accurately and were given a value of 999. These were not used for this plot B) Kernel density estimate for three out of the four datasets C) Scatterplot and Pearson’s correlation coefficient of values from pcSILAC against those reported by Wu et al. 1. Boisvert, F.-M., Ahmad, Y., Gierlinski, M., Charrière, F., Lamont, D., Scott, M., Barton, G., et al. (2012). Molecular & cellular proteomics: MCP, 11(3). 2. Wu, Z., Moghaddas Gholami, A., & Kuster, B. (2012). Molecular & cellular proteomics: MCP, 11(6). 3. Schwanhäusser, B., Busse, D., Li, N., Dittmar, G., Schuchhardt, J., Wolf, J., Chen, W., et al. (2011). Nature, 473(7347), 337–42. (TIF) [file pone.0080423.s014.tif]

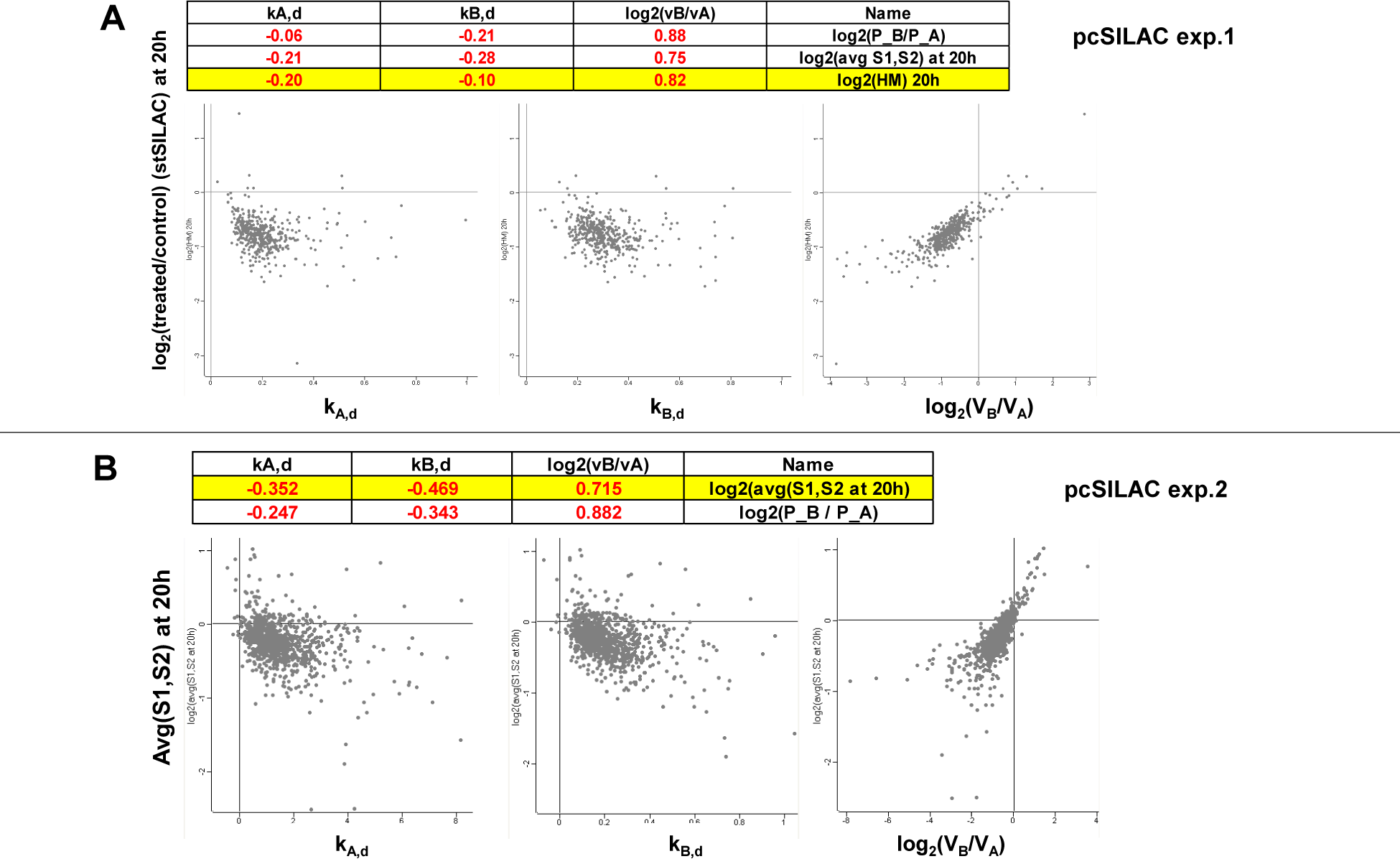

Supplement: Figure S15 — Correlation between synthesis rates, decay rate constants and changes in net protein levels in the two pcSILAC experiments. Pearson’s r values are shown. Plots represent the datasets highlighted in yellow in the tables. A) Correlation of kA, kB and log2(VB/VA) with the experimentally determined stSILAC ratio at 20h in experiment 1. B) Correlation of kA, kB and log2(VB/VA) with computationally derived ratio of net protein levels in experiment 2 (20h). (TIF) [file pone.0080423.s015.tif]

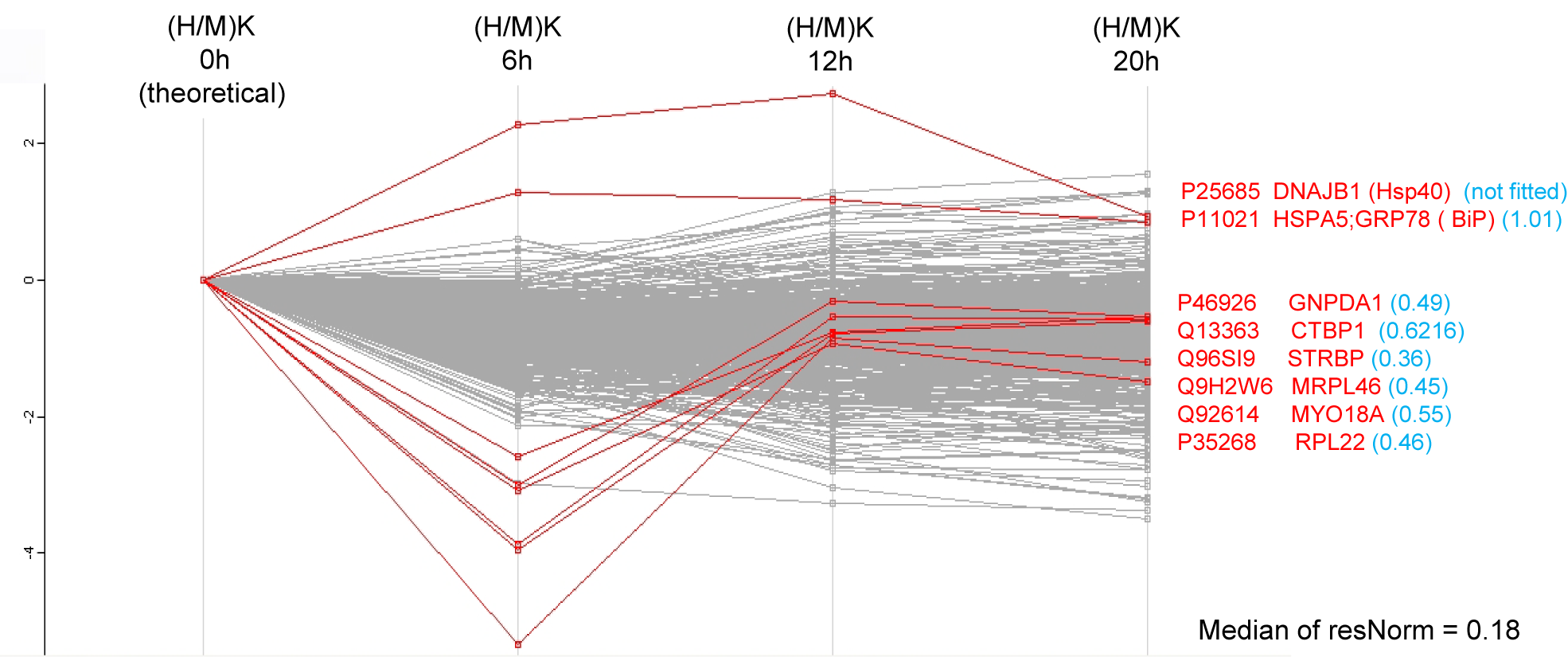

Supplement: Figure S16 — Example of proteins with poor fitting to the model. Ratios (H/M)K, measuring newly synthesized protein levels from experiment 2 are shown. Values are after correction for mixing inequality and log2 transformation. Some proteins with apparent multiphasic behavior are shown in red, together with their value of the ResNorm parameter (Blue), which is a measure of the quality of the fitting. Most proteins highlighted here have values of ResNorm well above the median (0.18) of ResNorm for the whole population. It is to be noted however that, since the fitting is performed on 6 series of ratios, deviations due to noise in one series can be compensated by other values. DNAJB1 and GRP78 were quantitated with large numbers of peptides and thus their values should be reliable and reflect a complex temporal dynamic of changes. (TIF) [file pone.0080423.s016.tif]
