# Supplementary material for: A Novel Pulse-Chase SILAC Strategy Measures Changes in Protein Decay and Synthesis Rates Induced by Perturbation of Proteostasis with an Hsp90 Inhibitor
Source: PLoS One. 2013 Nov 27;8(11):e80423. doi: 10.1371/journal.pone.0080423 (PMC3842330; doi:10.1371/journal.pone.0080423)
Supplement: Supplementary Information S1 — Main supporting information including i) detailed discussion of isotope patterns of various types of peptides ii) Description of the mathematical model and computational framework for pcSILAC including derivation of all kinetic parameters and fluxes iii) all supplementary figures S1-S16 in one file to facilitate viewing after download (PDF). (PDF) [file pone.0080423.s022.pdf]

A novel pulse-chase SILAC strategy measures changes in protein decay and synthesis rates induced by perturbation of proteostasis with an Hsp90 inhibitor

Ivo Fierro-Monti‡, Julien Racle#[Ø], Celine Hernandez‡¶, Patrice Waridel‡, Vassily Hatzimanikatis#[Ø] and Manfredo Quadroni‡*

‡ Center for Integrative Genomics, Faculty of biology and Medicine, University of Lausanne, Switzerland ;

Laboratory of Computational Systems Biotechnology, École Polytechnique Fédérale de Lausanne (EPFL), Lausanne, Switzerland

[Ø] SIB Swiss Institute of Bioinformatics, Lausanne

¶ SIB Swiss Institute of Bioinformatics, Vital-IT group, Lausanne

Supplementary information

Abbreviations : ODE, ordinary differential equations

Contents

Isotope patterns of doubly labeled peptides 3

Incorporation ratios of K- vs. R-containing peptides 4

Reproducibility of pcSILAC measurements 5

Mathematical formulation of variables linked to pcSILAC experiments 5

Calculation of pseudo-absolute protein concentrations in the control sample (p_A) using iBAQ scores 7

Model and calculation of kinetic parameters 8

Equations of evolution of protein concentrations 10

Normalization, calculation of mixing ratios and data correction 11

Cleavage of proteins into peptides 13

Experimentally measured quantities and their integration into the model 14

Fitting the data to the model 14

Derivation of V_A, V_B, p_B 16

Calculation of fluxes 17

Additional notes 18

Isotope patterns of doubly labeled peptides

pcSILAC labeling produces isotope patterns more complex than standard SILAC in the case of peptides containing more than one labeled amino acid, i.e. 2 x K-, R+K- or 2 x R-containing peptides resulting from missed trypsin cleavages (including RP, KP sequences).

"**Pure" double-labeled (2 x K, 2 x R) peptides** were used for pcSILAC quantification after we manually inspected their isotope patterns at t=6h,12h in both samples before and after mixing. Indeed, such peptides could be showing partial labeling peaks, i.e. peptides carrying one heavy and one light Lys, deriving from label mixing ("old" label with "new" label) occurring especially in the first hours after medium exchange (Supplementary Figures S2-3 and S5-6). Analysis of double-labeled (2 x K and 2 x R) peptides has been previously used to quantify the amount of label mixing [1] [2]. We found that, in general, partial labeling peaks were of negligible intensity (<5% of base peak, Supplementary Figures S5-S6), indicating that the replacement of the intracellular label, when it occurred, was nearly complete and thus label mixing did not influence the quantitation obtained at the time points considered.

Mixed double-labeled (1xK, 1xR) peptides on the other hand can present significantly more complex isotope patterns when all possibilities for incorporation, including label mixing, are considered (Supplementary Figure S3). We verified MS signals for 20 distinct peptides from various proteins at 6h and 20h and observed that their isotope patterns were indeed complex and did fit the predictions (Supplementary Figure S5). Like for pure labeled peptides, signals derived from label mixing (old+new label) were of low or very low intensity. Interestingly, such mixed KR- or RK-containing peptides could theoretically be used for quantitation purposes since they contain specific information e.g. on total protein amounts in the two samples (each peak corresponds to the four labeling species R6K0, R10K0, R0K4, R0K8). However, their complex isotope patterns are overlapping and difficult to disentangle. To our knowledge, at the moment there is no software tools that can exploit the information contained in these signals for quantitation.

Incorporation ratios of K- vs. R-containing peptides

Since R and K are used independently, we tested if there were differences in the ratios measured on peptides containing one or the other amino acid. Such differences could arise, for example, due to different incorporation kinetics. For this purpose we analyzed pcSILAC cell extracts at the three time points from control and GA-treated cells before mixing, to measure new/old ratios which express label incorporation ratios. Non-normalized (H/L) or (M/L) ratios obtained with R and K showed highly correlated incorporation kinetics (Supplementary Figure S4) in both conditions with linear correlations coefficients R2 above 0.95 and intercepts virtually at 0. However, the slope in both treated and control samples was reproducibly between 0.8 and 0.9 at all time points, meaning that for the same protein the ratios measured on K-containing peptides were systematically lower than those measured on R peptides. This cannot be due to a different size in the initial pool of amino acid which needs to be exchanged, since this would have given rise to a delay in time (a non-zero intercept on the plot) rather than a different slope. A possible interpretation would be that of a persistent (until t=20h) defect in incorporation of new label for K compared to R, for example through the recycling of K from degradation of preexisting proteins, which would constantly release "old" label. Such a phenomenon has already been postulated by Boisvert et al. [3], who also reported values close to 20%, without analyzing separately incorporation of R and K. However, amino acid recycling was not compatible with our observation that the degree of label mixing assessed on the spectra of multiply-labeled peptides was minimal in our samples. Furthermore, examination of some standard SILAC datasets acquired on the same cells, after an identical sample processing with separation of the values for K,R, revealed that ratios calculated before normalization showed a similar bias, with K-based ratios 10-12% lower than R-based ratios. Such differences typically disappear in normal SILAC data analysis because the MaxQuant software normalizes peptide ratios separately for R- and K-containing peptides. The observation of a similar bias also in standard SILAC samples which are 100% labeled tends to exclude amino acid pools and incorporation mechanisms as a possible cause. The reason(s) for these differences between R and K ratios are presently unknown to us. We can speculate that phenomena related to the isotopic labeled amino acids used may play a role. The satellite peak at -1 a.m.u visible for K4- and K8–labeled peptides (Supplementary Figure S6) could somewhat cause an underestimation of the medium or heavy K isotopes. Alternatively, an isotope effect in trypsin digestion similar to what recently

described [4] could be responsible for this difference. Different rates of cleavage for the light, medium and heavy labels of R- and K-peptides were therefore integrated in the kinetic model based on pcSILAC data (see below). Systematic biases linked to the amino acid are occasionally detected in standard SILAC but are systematically removed through normalization, which for this reason is performed separately by MaxQuant ([5] and J.Cox, personal communication).

Reproducibility of pcSILAC measurements

Reproducibility between pcSILAC experiments was assessed at 20h using first the (H/M) ratios. Pearson's correlation r values between medians of replicates of experiments 1 and 2 were 0.88 for $(H/M)_K$ and 0.27 for $(H/M)_R$ (supplementary Fig. S7C). The lower r value for R could be explained by the much smaller spread of the data for $(H/M)_R$, i.e. by the fact that most values of $(H/M)_R$ are very close to 1 and therefore the correlation is strongly influenced by technical noise, which tends to be constant. Indeed, examination of the values of ratio $(H/L)_R$ showed that for other R-based ratios correlation between experiments and replicates was good (r >0.85, Supplementary Fig. S7). These observations also suggest that using all six ratios as an input for further calculations could be an advantage as it may permit to correct for measurement errors and increase robustness of fitting.

Mathematical formulation of variables linked to pcSILAC experiments

Variables and indexes were defined as follows (see main text for description):

For <u>control</u> (untreated) cells:

$U_i(t)$ is the amount of protein *i* synthesized before t=0 (label : R6, K0)

$V_i(t)$ is the amount of protein *i* synthesized after t=0 (label : R0, K4)

For treated (+GA) cells:

$X_i(t)$ is the amount of protein *i* synthesized before t=0 (label : R10, K0)

$Y_i(t)$ is the amount of protein *i* synthesized after t=0 (label : R0, K8)

In all indexes:

A refers to the control (untreated) cells

B refers to GA-treated cells

For simplicity, the indexes and time dependence will be omitted in most equations. The following relationships can be derived:

$$\begin{cases} \left(\frac{H}{M}\right)_R = \frac{X}{U} & \left(\frac{H}{M}\right)_K = \frac{Y}{V} \quad (1)\text{-}(2) \\ \left(\frac{H}{L}\right)_R = \frac{X}{Y+V} & \left(\frac{H}{L}\right)_K = \frac{Y}{X+U} \quad (3)\text{-}(4) \\ \left(\frac{M}{L}\right)_R = \frac{U}{Y+V} & \left(\frac{M}{L}\right)_K = \frac{V}{X+U} \quad (5)\text{-}(6) \end{cases}$$

where the ratios $\left(H/M\right)_R, \left(H/L\right)_R, \ldots$ are the experimentally measured values from mass spectra for each protein (i.e. in the experiment differently labeled amino acids are incorporated at different times and in different cultures, and it is then possible to measure the relative abundance of the differently labeled species for the same protein).

Calculation of incorporation ratio: by inserting (1) into (6), resp. (2) into (5) it is possible to obtain the incorporation ratio $\left(\frac{"new"}{"old"}\right)$ for the untreated (medium) sample in two possible ways using different variables:

$$\frac{V}{U} = \left(\frac{M}{L}\right)_K \left(1 + \left(\frac{H}{M}\right)_R\right) \quad (7) \; ; \quad \frac{V}{U} = \left(\frac{1}{\left(\frac{M}{L}\right)_R \left(1 + \left(\frac{H}{M}\right)_K\right)}\right) \quad (8)$$

Similarly, by inserting (2) into (3) or (1) into (4) the same can be obtained for the treated (heavy) sample :

$$\frac{Y}{X}=\left(\frac{H}{L}\right)_K\left(1+\frac{1}{\left(\frac{H}{M}\right)_R}\right) \quad (9)\ ; \quad \text{or} \quad \frac{Y}{X}=\left(\frac{1}{\left(\frac{H}{L}\right)_R\left(1+\frac{1}{\left(\frac{H}{M}\right)_K}\right)}\right) \quad (10)$$

Now, continuing to work with these equations it is possible to obtain formulas to calculate the total ratio $S=\frac{X+Y}{U+V}$ in the form of two very symmetrical expressions:

$$S_1=\frac{\left(\frac{H}{L}\right)_R+\left(\frac{\left(\frac{H}{M}\right)_K}{1+\left(\frac{H}{M}\right)_K}\right)}{\left(\frac{M}{L}\right)_R+\left(\frac{1}{1+\left(\frac{H}{M}\right)_K}\right)} \quad (11)\ ; \quad \text{or} \quad S_2=\frac{\left(\frac{H}{L}\right)_K+\left(\frac{\left(\frac{H}{M}\right)_R}{1+\left(\frac{H}{M}\right)_R}\right)}{\left(\frac{M}{L}\right)_K+\left(\frac{1}{1+\left(\frac{H}{M}\right)_R}\right)} \quad (12)$$

S_1 and S_2 should correspond to values measured for example with a standard SILAC experiment (before normalization).

Calculation of pseudo-absolute protein concentrations in the control sample (p_A) using iBAQ scores

The use of the integrated peptide ion intensities generated by the MaxQuant software to estimate protein concentrations has been described previously (iBAQ) [6]. The standard output table *proteinGroups.txt* generated by MaxQuant contains the signal intensities for the three channels L, M and H for every protein, regardless of the sequence of the peptide (K,R peptides are counted together). Given the

characteristics of pcSILAC data, the M and H channels are, respectively, related to the total amounts of protein in the control (U+V) and treated (X+Y) samples :

$$\begin{cases} I_M \approx U + V \\ I_H \approx X + Y \end{cases} \tag{13}$$

Where I_M and I_H are the intensities of the signal in the corresponding channels. For example, the iBAQ score for a protein *i* in the sample A (control) is thus

$$iBAQ\_score_{i,A} = \frac{I_M}{n_{\det,i}} \tag{14}$$

Where n_{det} is the number of detectable peptides for the protein *i* [6]. Since no data for absolute concentrations of internal standards were available in our datasets, we expressed the concentration of proteins in arbitrary units relative to the median of all iBAQ scores at one time point. We verified that the values obtained in this way for the sample A (control) were very similar at the 3 time points (data not shown) after which we obtained a general value for the concentrations $p_{i,A}$ as:

$$p_{i,A} = \frac{mean_{timepo\,\text{int}\,s}(iBAQ\_score_{i,A})}{median(\sum_i mean_{timepo\,\text{int}\,s}(iBAQ\_score_{i,A}))} \tag{15}$$

It is important to remember that the concentrations in sample *A* (untreated cells) are assumed to be constant since the system is considered to be at steady-state.

Model and calculation of kinetic parameters

The meaning of the individual variables linked to protein concentrations and their evolution can be summarized as follows :

Culture	t < 0	0 < t < t$_d$	t$_d$ < t
A (untreated)	$\xrightarrow{V_A} u \xrightarrow{k_{A,app}} \varnothing$ $v_0 = 0$	$u \xrightarrow{k_{A,app}} \varnothing$ $\xrightarrow{V_A} v \xrightarrow{k_{A,app}} \varnothing$	$u \xrightarrow{k_{A,app}} \varnothing$ $\xrightarrow{V_A} v \xrightarrow{k_{A,app}} \varnothing$

**B (treated at time t = t$_d$)**	$\xrightarrow{V_A} x \xrightarrow{k_{A,app}} \varnothing$ $y_0 = 0$	$x \xrightarrow{k_{A,app}} \varnothing$ $\xrightarrow{V_A} y \xrightarrow{k_{A,app}} \varnothing$	$x \xrightarrow{k_{B,app}} \varnothing$ $\xrightarrow{V_B} y \xrightarrow{k_{B,app}} \varnothing$

The time of exchange of label was defined as t=0, while t_d is the time of start of drug treatment. First order decay and synthesis kinetics were postulated. The four variables u,v,x,y describe the concentrations of proteins synthesized before (u,x), resp. after label exchange (v,y). V_i (with i=A,B) is the rate of synthesis, while $k_{i,app}$ (with i=A,B) is the apparent decay rate constant, i.e. the constant determined from the data before correction by the cell growth rate.

Here we present in more detail the derivation of some equations, the explicit solutions of the ODE's and further parameters of the model.

Here we will show how the equation (1) from the main text is derived from the mass balance equations. The total mass balance describes the evolution of the abundance of a given component, here for a protein:

$$\frac{dP_A}{dt} = V_A^c \cdot V_A - k_{A,d} \cdot P_A \qquad (16)$$

where P_A is the abundance of the protein in one cell (with index *A* to refer to the control cell cultures; we emphasize that P_A is an abundance and not a concentration, so the units of P_A could be, for example, *moles*), V_A^c is the volume of the cell, V_A is the synthesis rate for this protein species (in $\left[mol/\left(L \cdot s\right)\right]$) and $k_{A,d}$ is the protein decay rate constant.

But then, instead of looking at the abundance, we consider the concentration of the protein (p_A), which is directly related to the abundance by

$$P_A = p_A \cdot V_A^c \qquad (17)$$

Taking the time-derivative of (17) and applying the chain rule gives:

$$\frac{dP_A}{dt} = \frac{d\left(p_A \cdot V_A^c\right)}{dt} = V_A^c \cdot \frac{dp_A}{dt} + p_A \cdot \frac{dV_A^c}{dt} = V_A^c \cdot \frac{dp_A}{dt} + p_A \cdot V_A^c \cdot \frac{1}{V_A^c}\frac{dV_A^c}{dt}$$
$$= V_A^c \cdot \frac{dp_A}{dt} + p_A \cdot V_A^c \cdot \mu_A \qquad (18)$$

where μ_A is the growth rate (or dilution term).

Combining equations (16) and (18), keeping only the concentration-derivative on the left and dividing by the volume of the cells gives the wished equation:

$$\frac{dp_A}{dt} = \frac{1}{V_A^c} \cdot \left(V_A^c \cdot V_A - k_{A,d} \cdot p_A \cdot V_A^c - p_A \cdot V_A^c \cdot \mu_A \right)$$
$$= V_A - \left(k_{A,d} + \mu_A \right) \cdot p_A = V_A - k_{A,app} \cdot p_A \quad (19)$$

with

$$k_{A,app} = k_{A,d} + \mu_A \quad (20)$$

The calculation of the growth rate μ_A is described below in the "Additional notes".

For all subsequent steps we will thus use the apparent decay rate, $k_{A,app}$.

Equations of evolution of protein concentrations

Then we can solve the ODE equation (19) and similar ones that represent the evolutions of the proteins with different markers. Considering the evolution of the various variables *u,v,x,y* as described above and assuming steady state at $t < 0$ we have

$$u(t=0) = u_0 = x_0 = \frac{V_A}{k_{A,app}} \quad (21)$$

And for example, for $t > 0$

$$\begin{cases} \frac{du}{dt} = -k_{A,app} \cdot u \\ \frac{dv}{dt} = V_A - k_{A,app} \cdot v \end{cases} \quad (22)$$

Solving the equations for *u* and *v* gives

$$\begin{cases} u(t) = u_0 \cdot e^{-k_{A,app} \cdot t} \\ v(t) = u_0 \left(1 - e^{-k_{A,app} \cdot t} \right) \end{cases} \quad (23)$$

Now, *x* and *y* follow the same evolution than *u, v,* until t_d, so at $t = t_d$:

$$\begin{cases} x(t_d) = x_d = \dfrac{V_A}{k_{A,app}} \cdot e^{-k_{A,app} \cdot t_d} \\ y(t_d) = y_d = \dfrac{V_A}{k_{A,app}} \cdot \left(1 - e^{-k_{A,app} \cdot t_d}\right) \end{cases} \quad (24)$$

And solving the evolution after t_d gives:

$$\begin{cases} x(t) = \dfrac{V_A}{k_{A,app}} \cdot e^{-k_{A,app} \cdot t_d - k_{B,app} \cdot (t - t_d)} \\ y(t) = \dfrac{V_B}{k_{B,app}} - \dfrac{V_A}{k_{A,app}} \cdot e^{-k_{A,app} \cdot t_d - k_{B,app} \cdot (t - t_d)} + \left(\dfrac{V_A}{k_{A,app}} - \dfrac{V_B}{k_{B,app}} \right) \cdot e^{-k_{B,app} \cdot (t - t_d)} \end{cases} \quad (t > t_d) (25)$$

So, equations (23) and (25) describe the evolution of the proteins in both cultures until cell lysis and protein extraction.

Normalization, calculation of mixing ratios and data correction

In standard proteome analyses by SILAC, the peptide ratios obtained are typically normalized on the basis of the median of the global distribution of all ratios, assuming that the overall composition of the two samples is comparable and that when the median of ratios is not 1.0 the difference is caused by imperfect (not 1:1) mixing of the two extracts. This is however an approximation, because the median of the individual protein ratios does not necessarily coincide with the actual total protein mixing ratio, which is based on an experimental measurement of the total protein concentrations of a sample. For example, the total protein mixing ratio can be influenced by the changes in a few very abundant proteins, while the same changes will not significantly affect the median of protein ratios. Nevertheless, in most standard SILAC experiments global normalization is an appropriate way of compensating for an imperfect mixing ratio.

In pcSILAC measurements, the values for $(H/M)_R$ and $(H/M)_K$ for the control and GA-treated sample before normalization showed important global differences (Supplementary Figure S9), indicating a skewed composition of the treated sample

which contained overall less newly synthesized protein and more pre-existing protein than the control. Normalization eliminated such differences in the data, yielding distributions of $(H/M)_R$ and $(H/M)_K$ ratios centered around 1 (Figure 2, Supplementary Figure S9). However, for calculations of kinetic parameters (see below) it was necessary to use non normalized values of all the ratios measured by pcSILAC, namely (H/M) (H/L) (M/L) for both K and R. Such values had to be accurately corrected for inequalities in sample mixing ratios, in some other way than global normalization which can heavily bias the absolute values of all SILAC ratios.

As described above, the total values of intensities I_M and I_H are related to the amounts of a protein in the control (A) and inhibitor treated sample (B) (through the iBAQ score). Therefore, the mixing ratio λ can be calculated as:

$$\lambda = \frac{B}{A} = \frac{\sum_i iBAQ\_score_{i,B}}{\sum_i iBAQ\_score_{i,A}} \tag{26}$$

The mixing ratios so determined were used for example to correct the values of all non-normalized ratios used for the plots in Supplementary Fig. S9. More importantly they were integrated in the calculations of kinetic parameters. We thus introduced the mixing ratio λ and we defined the amounts of the proteins in the mixed sample as *U, V, X* and *Y* (i.e. in contrast to *u,v,x,y,* which represent the cellular concentrations). Without loss of generality, we can define the reference amount as the one from the control cells so that we then have in the mixed sample:

$$\begin{cases} U = u \\ V = v \\ X = \lambda \cdot x \\ Y = \lambda \cdot y \end{cases} \tag{27}$$

Cleavage of proteins into peptides

The next step in the model construction was accounting for the systematic discrepancy between ratios measured with K- vs. R-containing peptides. We assumed that the difference in quantitation originated from a different efficiency in the cleavage of proteins during the enzymatic digestion step, with the heavy and medium isotopes being cleaved less efficiently than the light ones.

We described the evolution of the protein amount during the cleavage into peptides with the following scheme:

$$\begin{array}{ll} U_R^a \xrightarrow{k_M} U_R & X_R^a \xrightarrow{k_H} X_R \\ U_K^a \xrightarrow{k_L} U_K & X_K^a \xrightarrow{k_L} X_K \\ V_R^a \xrightarrow{k_L} V_R & Y_R^a \xrightarrow{k_L} Y_R \\ V_K^a \xrightarrow{k_M} V_K & Y_K^a \xrightarrow{k_H} Y_K \end{array} \qquad (28)$$

where U_R^a represents the amount of protein U that are still available for cleavage, U_R represents the peptides containing the amino acid R (this is what is observed in mass spectra), and k_L, k_M and k_H correspond to the rates of cleavage into peptides for the proteins containing Light, Medium or Heavy amino acids markers respectively.

For the initial conditions for this "cleavage evolution" we have the values present when the cells were harvested and lysed, for example:

$$X_R^a(s=0) = X_K^a(s=0) = X(t_1) = \lambda \cdot x(t_1) \qquad (29)$$

where s is used here for the "time" parameter for the system of protein cleavage (this time is independent from the time t of evolution of the proteins in the cells), and t_1 is the time when the cells where harvested and lysed.

Solving for the evolution of the system from the scheme (28), for example for U_R gives:

$$U_R(t=t_1, s) = U(t_1)\cdot\left(1-e^{-k_M \cdot s}\right) = U(t_1)\cdot Q_M \qquad (30)$$

In this equation, we have replaced the term with the rate of cleavage by Q_M, whereby by definition Q_M is constrained to lie between 0 and 1. This replacement can be justified by considering that the cleavage time s is independent of the measurements and was the same for all samples at all time points (the same was true for the amount of enzyme used and other cleavage conditions).

Similarly for the other proteins we get these relationships:

$$\begin{cases} U_R(t_1) = Q_M \cdot u(t_1) & X_R(t_1) = Q_H \cdot \lambda \cdot x(t_1) \\ U_K(t_1) = Q_L \cdot u(t_1) & X_K(t_1) = Q_L \cdot \lambda \cdot x(t_1) \\ V_R(t_1) = Q_L \cdot v(t_1) & Y_R(t_1) = Q_L \cdot \lambda \cdot y(t_1) \\ V_K(t_1) = Q_M \cdot v(t_1) & Y_K(t_1) = Q_H \cdot \lambda \cdot y(t_1) \end{cases} \tag{31}$$

Experimentally measured quantities and their integration into the model

Mass spectrometry data yield the ratio of the abundances of the peptides containing different labels, which are then used to infer the identified proteins and their quantitative ratios. For each protein there were 6 ratios measured:

$$\begin{cases} \left(\frac{H}{M}\right)_R = \frac{X_R}{U_R} & \left(\frac{H}{M}\right)_K = \frac{Y_K}{V_K} \\ \left(\frac{H}{L}\right)_R = \frac{X_R}{Y_R + V_R} & \left(\frac{H}{L}\right)_K = \frac{Y_K}{X_K + U_K} \\ \left(\frac{M}{L}\right)_R = \frac{U_R}{Y_R + V_R} & \left(\frac{M}{L}\right)_K = \frac{V_K}{X_K + U_K} \end{cases} \tag{32}$$

Note how in these equations we have used the indices *K* and *R* to indicate that what is really measured in the mass spectra are the peptides and not directly the proteins. The ratios were measured for 3 time-points in the experiment.

Fitting the data to the model

Fitting of the data, included several steps. Equations (32) were measured at 3 times, so for each protein there were 18 experimental data points that could be used to fit our parameters. Only proteins having valid ratios measured at all time

points were used for the calculations. The unknown parameters in the model were k_A, k_B, V_A, V_B for each protein, as well as the Q_L, Q_M and Q_H that were global coefficients assumed to be the same for all proteins. It is important to note that in the equations, the V_A or V_B never appear isolated but only as ratios V_B/V_A so it will only be possible to derive the ratio of the synthesis rates in the two conditions.

The flowchart describing the estimation of parameters is shown in Main Figure 3. In detail:

First step of fitting

In the first step of fitting, we fit simultaneously the data for 100 protein species, using exactly the experimental values, in order to get estimates for the Q_L, Q_M and Q_H. For this fitting, the equations for the peptides abundances, eq. (31), (23) and (25), were inserted into the expression of the measured quantities, eq. (32). MATLAB's least square fitting functionalities (*lsqcurvefit*) were used to obtain the parameter values.

Second step of fitting: obtaining k_A, k_B, V_B/V_A

In this second step, we use the values previously obtained for Q_L, Q_M and Q_H to ensure independence of the equations for the various protein species. This allowed doing the fit for each protein species to get the k_A, k_B and V_B/V_A of each protein separately.

In order to get more confidence in the data and simulate the natural fluctuations and noise present in mass spectrometry data, the fitting was repeated 100 times, whereby each time random noise (normally distributed, standard deviation 10% of the value) was added to the experimental values, similarly to what is done for bootstrap-methods. The fitting was performed with MATLAB (*lsqcurvefit*) using the same equations as in step 1, but performing a fit separately for the equations of each protein. After repeating 100 times the fitting, the average value of the fitted parameter as well as the standard deviation over these 100 fits were obtained. This procedure allowed seeing if the parameter was highly constrained by the experimental data or if the presence of noise in the data had a strong impact on the parameter values.

The simulation process allowed monitoring the quality of the fitting by verifying the convergence of the values and the residual norm of the error. The latter indicates the residual norm of the difference between the observed values and estimated from the fits. A value near of 0 indicates a good fit while a high value is for a poor fitting. A few proteins had high values for this parameter, which correlated with a complex behavior in time, and thus could not be fitted with the model adopted.
It is important to note that the corrections for the mixing ratios and for the cleavage rates for K,R peptides were very important to obtain good fitting of the pcSILAC ratios to the model as well as to avoid computational artifacts such as negative decay rates, which arose when these corrections were not done.

Derivation of V_A, V_B, p_B

From the values obtained up to this point, it is already possible to calculate the expected ratio of concentrations of total proteins p_B/p_A, considering that for the drug treated cells at their steady state:

$$y(t \to \infty) = \frac{V_B}{k_{B,app}} \quad (33)$$

And therefore, if p_A, p_B are the protein concentrations at steady state in the control or drug cultures, we have:

$$\frac{p_B}{p_A} = \frac{y(t \to \infty)}{v(t \to \infty)} = \frac{V_B}{V_A} \cdot \frac{k_{A,app}}{k_{B,app}} \quad (34)$$

Since V_B/V_A as well as $k_{A,app}$ and $k_{B,app}$ are known, p_B/p_A is fully determined. Furthermore, it is also possible to exploit the values of p_A determined using iBAQ scores to obtain more values:

$$V_A = p_A \, k_{A,app} \quad (35)$$

and then by substitution:

$$V_B = V_A \left(\frac{V_B}{V_A} \right)_{fitted} \quad (36)$$

And finally

$$p_B = \frac{V_B}{k_{B,app}} \quad (37)$$

It is important to note that p_B is the calculated protein concentration in the treated cells at steady state, i.e. with t=infinite. In conclusion, pcSILAC data allow the determination of all kinetic parameters, although in the present case the values of synthesis rates and protein concentrations remain in arbitrary units because of the lack of an internal standard allowing the calibration of intensities to obtain absolute concentrations.

Calculation of fluxes

To assess quantitatively the instantaneous changes in the treated sample we calculated fluxes due to synthesis, degradation and the dilution effect. Let's recall that the mass balance equation is:

$$\frac{dp_j}{dt} = V_j - k_{j,d} \cdot p_j - \mu_j . p_j \quad \text{where } j=A,B. \tag{38}$$

All the 3 terms on the right side can be considered as fluxes W:

$$\begin{cases} W_s = V_j \\ W_{d,j} = -k_{j,d} \cdot p_j \\ W_{\mu,j} = -\mu_j . p_j \end{cases} \tag{39}$$

and therefore

$$\frac{dp_j}{dt} = W_{s\,j} + W_{d,j} + W_{\mu,j} \tag{40}$$

For the control (A) the sum of the fluxes is zero by definition:

$$0 = W_{S,A} + W_{d,A} + W_{\mu,A}$$

On the other hand, the drug treated system will need some time to approach equilibrium. At each time point it is thus possible to estimate the contribution of the terms of degradation and synthesis to the total change.

$$\frac{dp_B}{dt}(t) = \frac{dp_B}{dt}(t) - \frac{dp_A}{dt}(t) = \left(W_{S,B}(t) - W_{S,A}(t)\right) + \left(W_{\mathrm{d},B}(t) - W_{\mathrm{d},A}(t)\right) + \left(W_{\mu,B}(t) - W_{\mu,A}(t)\right)$$

$$\frac{dp_B}{dt}(t) = \Delta W_S(t) + \Delta W_{\mathrm{d}}(t) + \Delta W_\mu(t) \tag{41}$$

Positive values of the ΔW for a given term indicate a positive (increase) influence on the concentration of the protein in sample B and conversely for negative values. The fluxes change as a function of time and the protein concentration p_B is used as an input. For this reason the values of the fluxes are most meaningful at $t=t_d$ and early time points (t=0,t=6h) because later the concentrations p_A and p_B may start to diverge significantly. This causes for example that

$$\Delta W_{\mathrm{d}}(t) = k_{A,d}.p_A(t) - k_{B,d}.p_B(t) \quad (42)$$

But since at later time points p_B has been influenced by other terms such as dilution and synthesis, the contributions of the individual fluxes cannot be clearly separated anymore.

Additional notes

Correction for growth rate

We have to assume that cells are growing exponentially and that the growth rate does not change during the experiment. In this case, the cell growth can be expressed by: $\frac{dN}{dt} = \mu N$ and therefore

$$N(t) = N(t_0)e^{\mu(t-t_0)} \quad (43)$$

where $N(t)$ is the biomass measured in million cells per ml and t and t_0 are the times at which the cells were counted.

Thus the correction term can be calculated as

$$\mu = \frac{\ln\left(\frac{N(t)}{N(t_0)}\right)}{t-t_0} \quad (44)$$

Calculation of half-lives

Assuming a first order exponential decay, the half-life of a species can be calculated very simply (for example for u) as follows:

$u(t) = u_0 e^{-k_{A,d}t}$ therefore $u(t_{1/2}) = \frac{u_0}{2} = u_0 e^{-k_{A,d}t_{1/2}}$

Thus the half-life is:

$$t_{1/2} = \frac{\ln(2)}{k_{A,d}} \quad (45)$$

References

1. Beynon RJ (2005) The dynamics of the proteome: strategies for measuring protein turnover on a proteome-wide scale. Briefings in functional genomics & proteomics 3: 382–390.

2. Schwanhäusser B, Gossen M, Dittmar G, Selbach M (2009) Global analysis of cellular protein translation by pulsed SILAC. Proteomics 9: 205–209. doi:10.1002/pmic.200800275.

3. Boisvert F-M, Ahmad Y, Gierlinski M, Charrière F, Lamont D, et al. (2012) A quantitative spatial proteomics analysis of proteome turnover in human cells. Molecular & cellular proteomics : MCP 11: M111.011429. doi:10.1074/mcp.M111.011429.

4. Konopka A, Boehm ME, Rohmer M, Baeumlisberger D, Karas M, et al. (2012) Improving the precision of quantitative bottom-up proteomics based on stable isotope-labeled proteins. Analytical and bioanalytical chemistry. doi:10.1007/s00216-012-6007-6.

5. Cox J, Mann M (2008) MaxQuant enables high peptide identification rates, individualized p.p.b.-range mass accuracies and proteome-wide protein quantification. Nature biotechnology 26: 1367–1372. doi:10.1038/nbt.1511.

6. Schwanhäusser B, Busse D, Li N, Dittmar G, Schuchhardt J, et al. (2011) Global quantification of mammalian gene expression control. Nature 473: 337–342. doi:10.1038/nature10098.

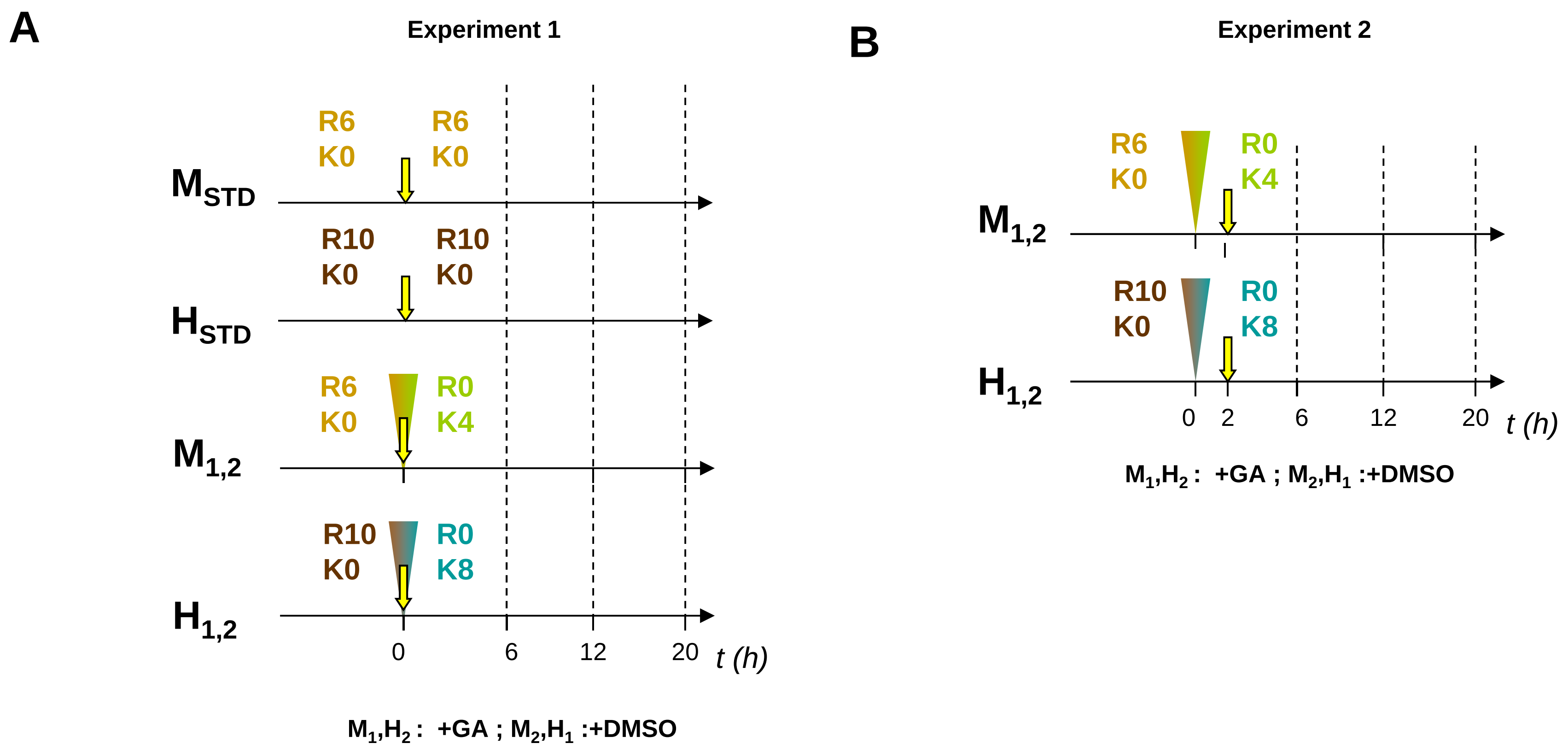

Supplementary Figure S2 : Samples and conditions of standard and pcSILAC experiments

Experimental design for pcSILAC experiments; the yellow arrow indicates treatment with geldanamycin (GA) or DMSO. Correspondingly numbered replicates were mixed (e.g. M1+H1, M2+H2) after total protein quantification. For replicates M1 and H1 the treatment was inverted, i.e. H1 received DMSO while M1 was treated with GA. **A)** experimental design for pcSILAC experiment 1 ; coloured triangles indicate medium exchange. The samples used as an internal standard SILAC experiment in pcSILAC experiment 1 were derived from the same culture used for pcSILAC labeling, therefore no medium exchange was done and quantitation was performed only on R (H/M ratio). **B**) same as A) for pcSILAC experiment 2 (no internal standard SILAC replicate was done in this experiment).

A

AA	Isotope	Abbrev.	Monoisotopic delta mass
Arg+6	$^{13}C_6$	R6	6.020129
Arg+10	$^{13}C_6{}^{15}N_4$	R10	10.008269
Lys+4	$^{2}H_4$	K4	4.025107
Lys+8	$^{13}C_6{}^{15}N_2$	K8	8.014199

B

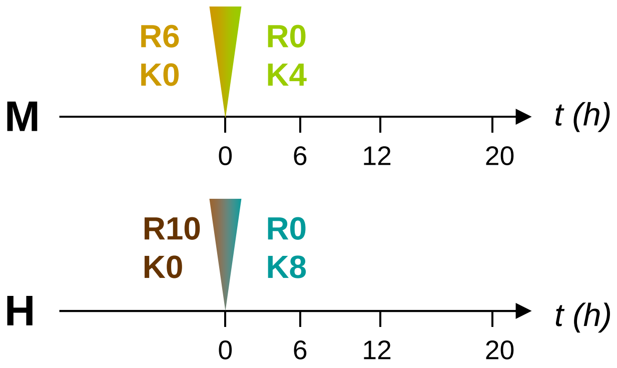

C

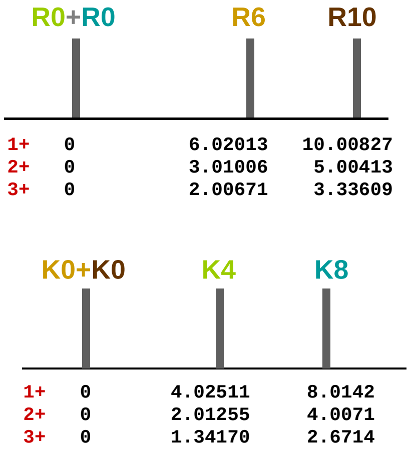

D

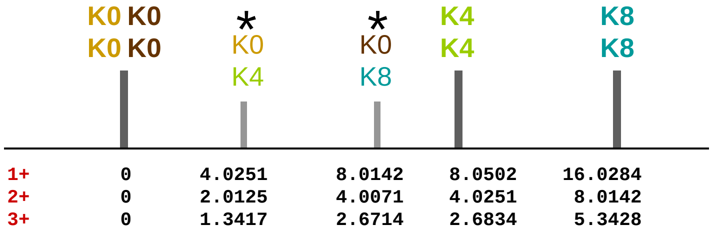

E

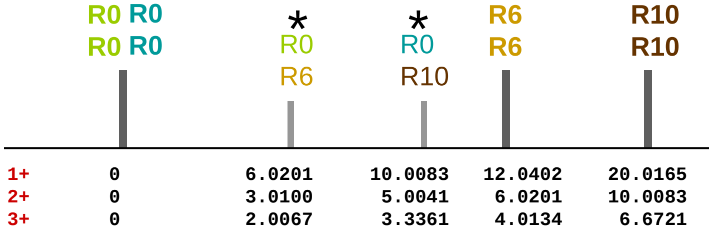

F

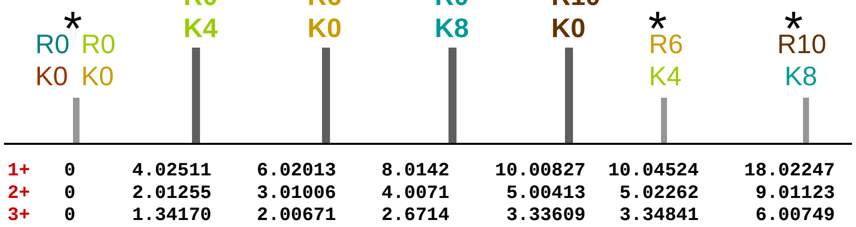

Supplementary Figure S3 : predicted and possible isotope peaks for various classes of tryptic peptides in pcSILAC

A) Isotope labelled amino acids used in the study and their monoisotopic mass shifts relative to light amino acids **B)** General Labelling scheme for pcSILAC with description of the isotopomers present in the media before/after medium exchange (represented as a coloured triangle) at t=0 **C)** possible isotope peaks of 1 x R- or 1 x K-containing peptides after mixing H+L pcSILAC samples, with expected mass shifts relative to a R0, resp. K0 peptide. **D)** Same as C) but for peptides containing 2 K residues. The occurrence of mixed-label peptides (*) during the phase of medium exchange was considered. **E)** Same as D) but for peptides containing 2x R residues. **F)** Same as D) but for peptides containing <u>**both**</u> 1 K and 1 R

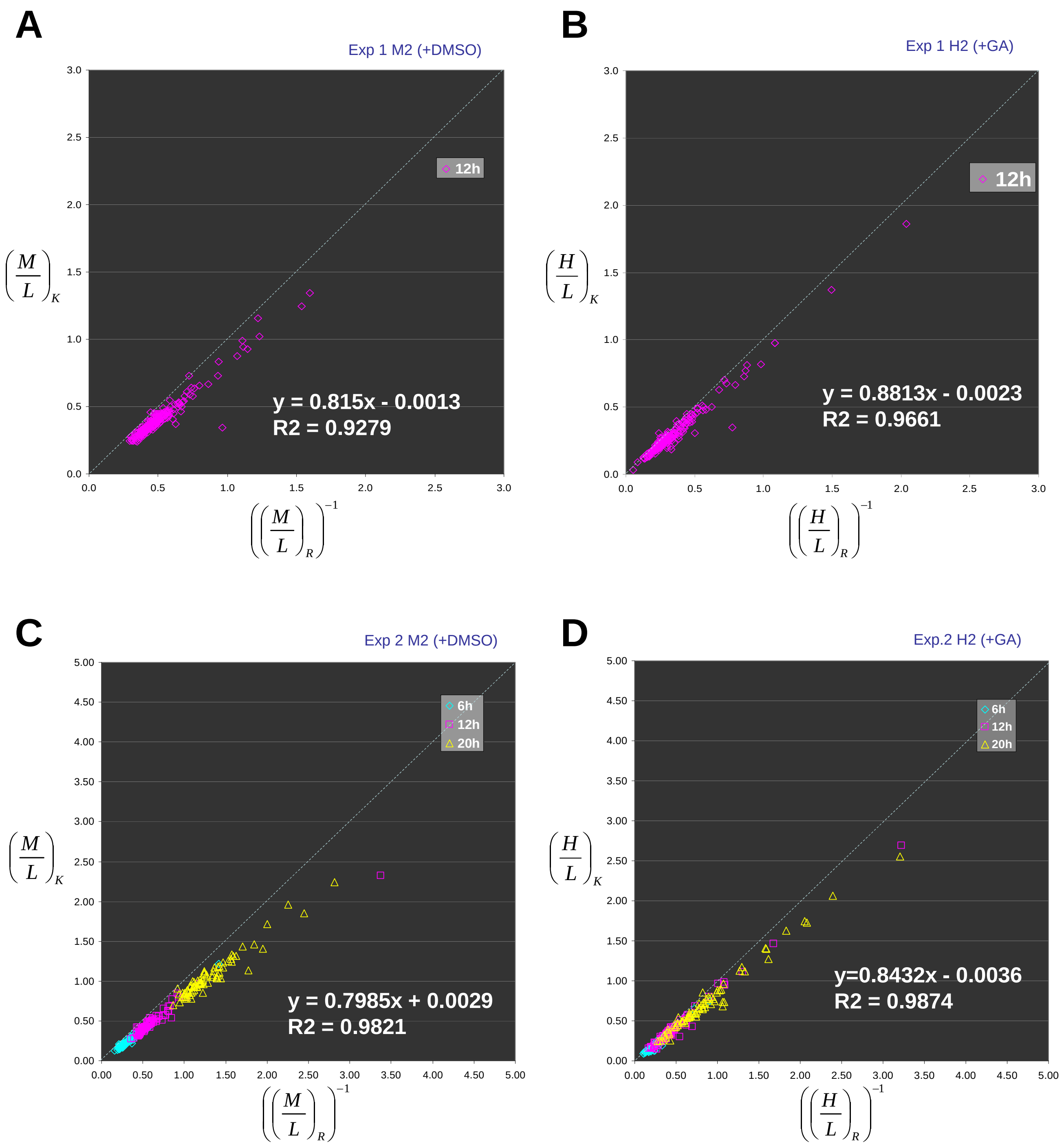

Supplementary figure S4. Differences in ratios measured for R- vs. K-containing peptides

« Medium- » and « heavy » extracts from cells at t=6,12 and 20h from pcSILAC experiments before mixing were separated by SDS-PAGE. Fractions in the range 30-150 kDa (A,B) or 60-100 kDa (C,D) were digested and analysed by MS. For every protein, ratios were determined separately based on K and R peptides. Data for a set of high-scoring proteins is represented as scatter plots (one point per protein, non log ratios). All values plotted correspond to (new label) / (old label) ratios.

(A) DMSO-treated cells (M,L labelled) from pcSILAC experiment 1 (200 proteins) at 12h **(B)** Geldanamycin-treated cells (H,L labelled) from pcSILAC experiment 1 at 12h (200 proteins). **(C)-**DMSO-treated control cells (M,L labelled) from pcSILAC experiment 2 (80 proteins) **(D)** same as (C) but for GA-treated cells (H,L labelled) **.**

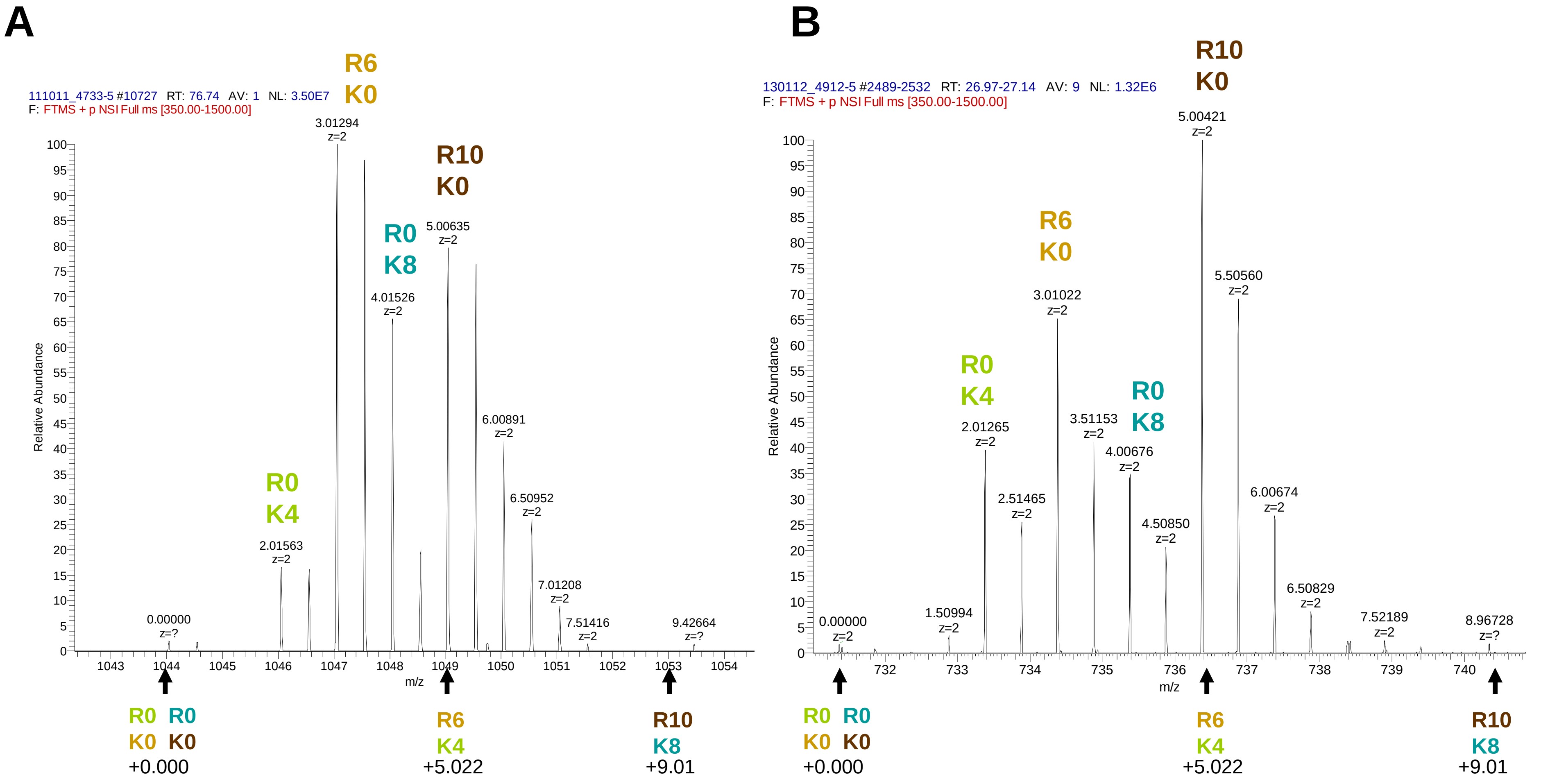

Supplementary Fig. S5 : peptides containing both R and K in pcSILAC

A) Isotope pattern observed for peptide **GHYTEGAELVDSVLDVVRK** (TBB2A_HUMAN) at t=6h in the H+L mix of pcSILAC experiment 1, replicate 2. The position of possible peaks resulting from label mixing (old label + new label) are indicated below the axis, with their expected mass shifts (see Supplementary figure 2) **B)** Same as A) but for peptide **GVAINMVTEEDKR** (IF4A1_HUMAN) at t=12h in pcSILAC experiment 1, replicate 1. Overall, the predicted isotope patterns for K+R-peptides were observed. Peaks resulting from label mixing (old label + new label) were sometimes detectable but had very low intensity (<3% of base peak, marked below x-axis) compared to homogeneously (old+old, new+new) labelled species. Spectra obtained for samples before mixing were also inspected with similar results.

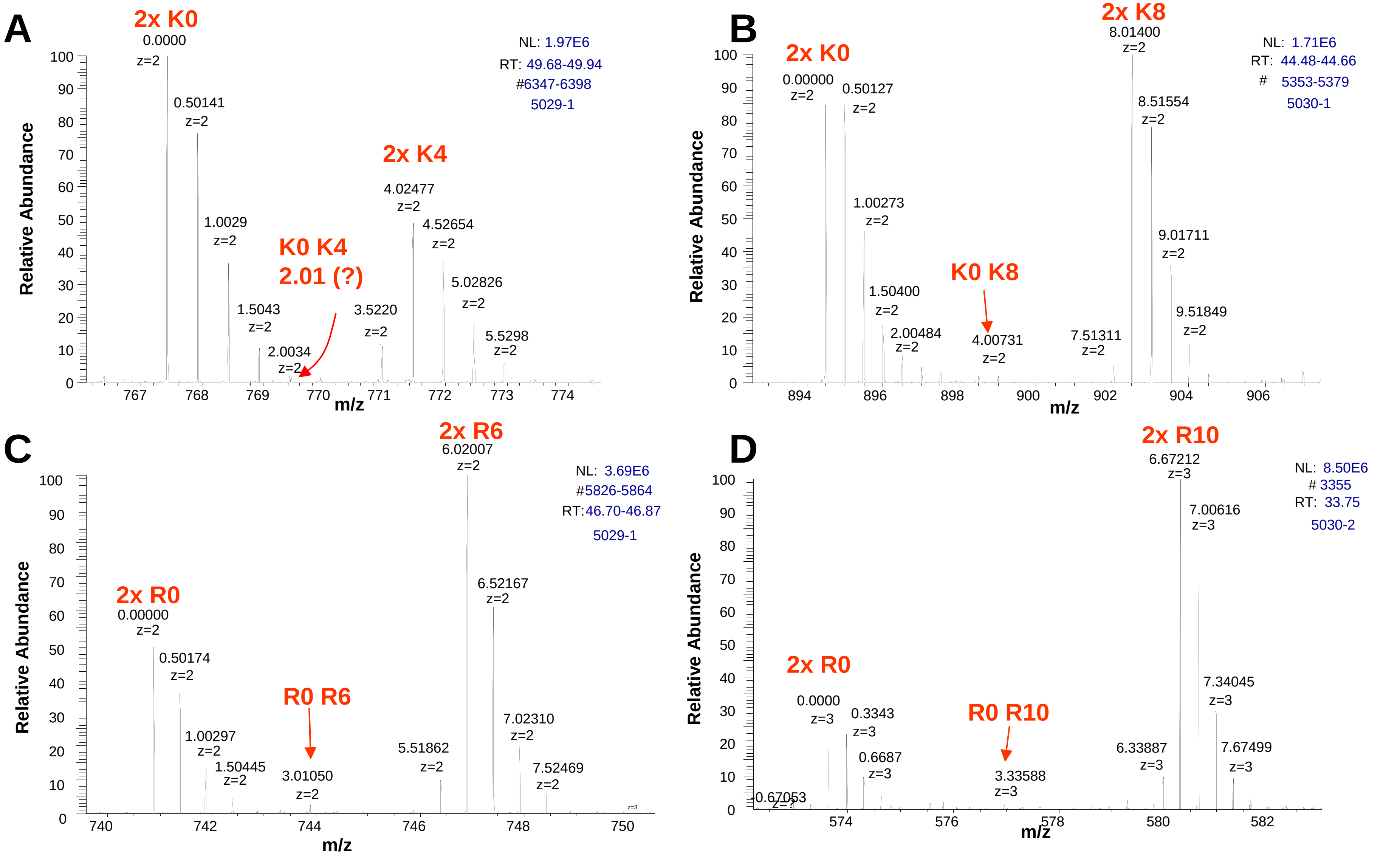

Supplementary figure S6: isotope patterns observed for KK- or RR-containing peptides in pcSILAC samples before mixing (experiment 1, t=12h). **A)** Isotope pattern observed for peptide KVESLQEEIAFLK (MH+=767.429, 2+, VIME_HUMAN) in the "light" sample of pcSILAC experiment 1, replicate 2 (+DMSO). Location of possible isotope peaks resulting from label mixing (old+new label) are indicated together with the expected mass shift relative to the fully "light" peak. **B)** peptide IINEPTAAAIAYGLDKK (MH+=894.4986,2+, HSP7C_HUMAN) , "heavy" sample, replicate 2, (+GA). **C)** Peptide ARFEELNADLFR (740.88112, 2+, HSP7C_HUMAN), "light sample, replicate 2, (+DMSO) **D)** NLDIERPTYTNLNR (580.304783,3+, TBA1B_HUMAN), "heavy" sample, replicate 2 (+GA). Intensity of the mixed-labelled peptide peaks remained below 5% of that of the newly synthesized peptide peak.

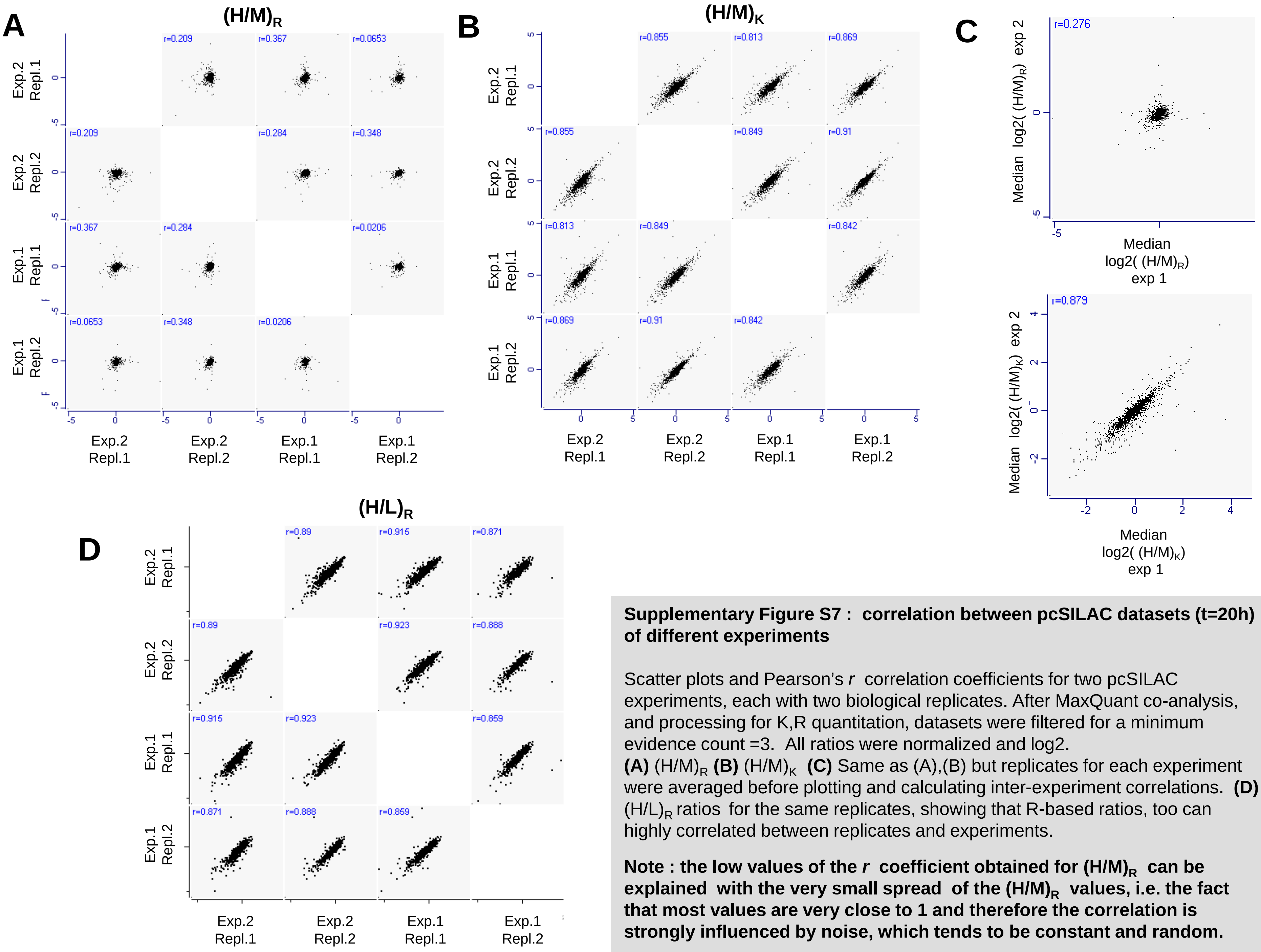

Supplementary Figure S7 : correlation between pcSILAC datasets (t=20h) of different experiments

Scatter plots and Pearson's *r* correlation coefficients for two pcSILAC experiments, each with two biological replicates. After MaxQuant co-analysis, and processing for K,R quantitation, datasets were filtered for a minimum evidence count =3. All ratios were normalized and log2. **(A)** $(H/M)_R$ **(B)** $(H/M)_K$ **(C)** Same as (A),(B) but replicates for each experiment were averaged before plotting and calculating inter-experiment correlations. **(D)** $(H/L)_R$ ratios for the same replicates, showing that R-based ratios, too can highly correlated between replicates and experiments.

Note : the low values of the *r* coefficient obtained for $(H/M)_R$ can be explained with the very small spread of the $(H/M)_R$ values, i.e. the fact that most values are very close to 1 and therefore the correlation is strongly influenced by noise, which tends to be constant and random.

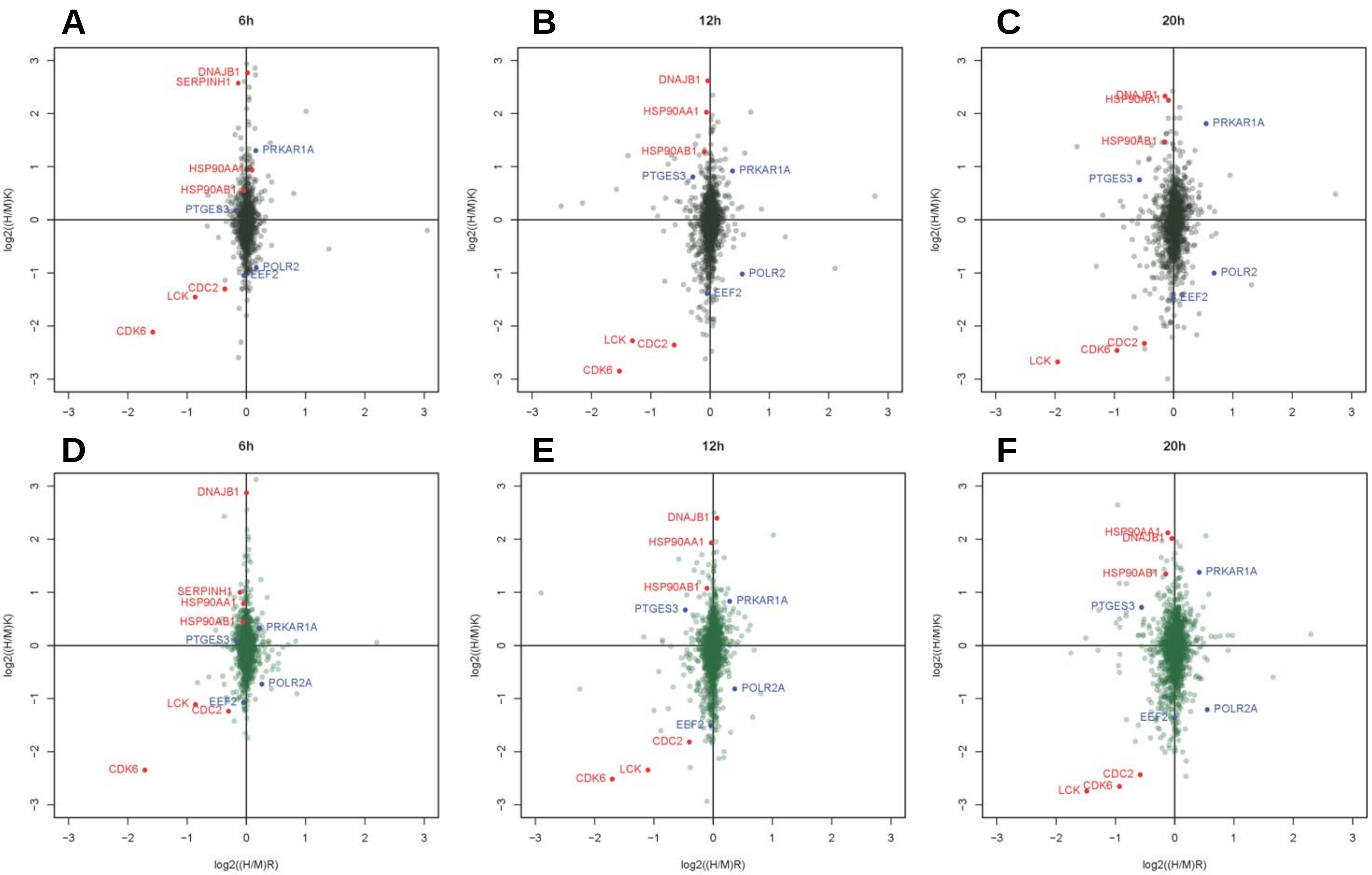

Supplementary Figure S8 Reproducibility of values of $(H/M)_K$,$(H/M)_R$ ratios for reference proteins and evolution in time in two pcSILAC experiments. Normalized ratios are shown. **A)-C)** pcSILAC experiment 1, **D)-F)** pcSILAC experiment 2. DNAJB1 = Hsp40 ; HSP90AA1 = Hsp90alpha ; HSP90AB1=Hsp90beta ; PTGES3=p23 ; PRAKAR1A = regulatory subunit of Protein kinase A ; POLR2A = DNA-directed RNA polymerase II subunit RPB1 ; EEF2=Elongation factor 2 ; CDC2=Cyclin-dependent kinase 1 ; LCK = tyrosine protein kinase Lck ; CDK6=cyclin-dependent kinase 6

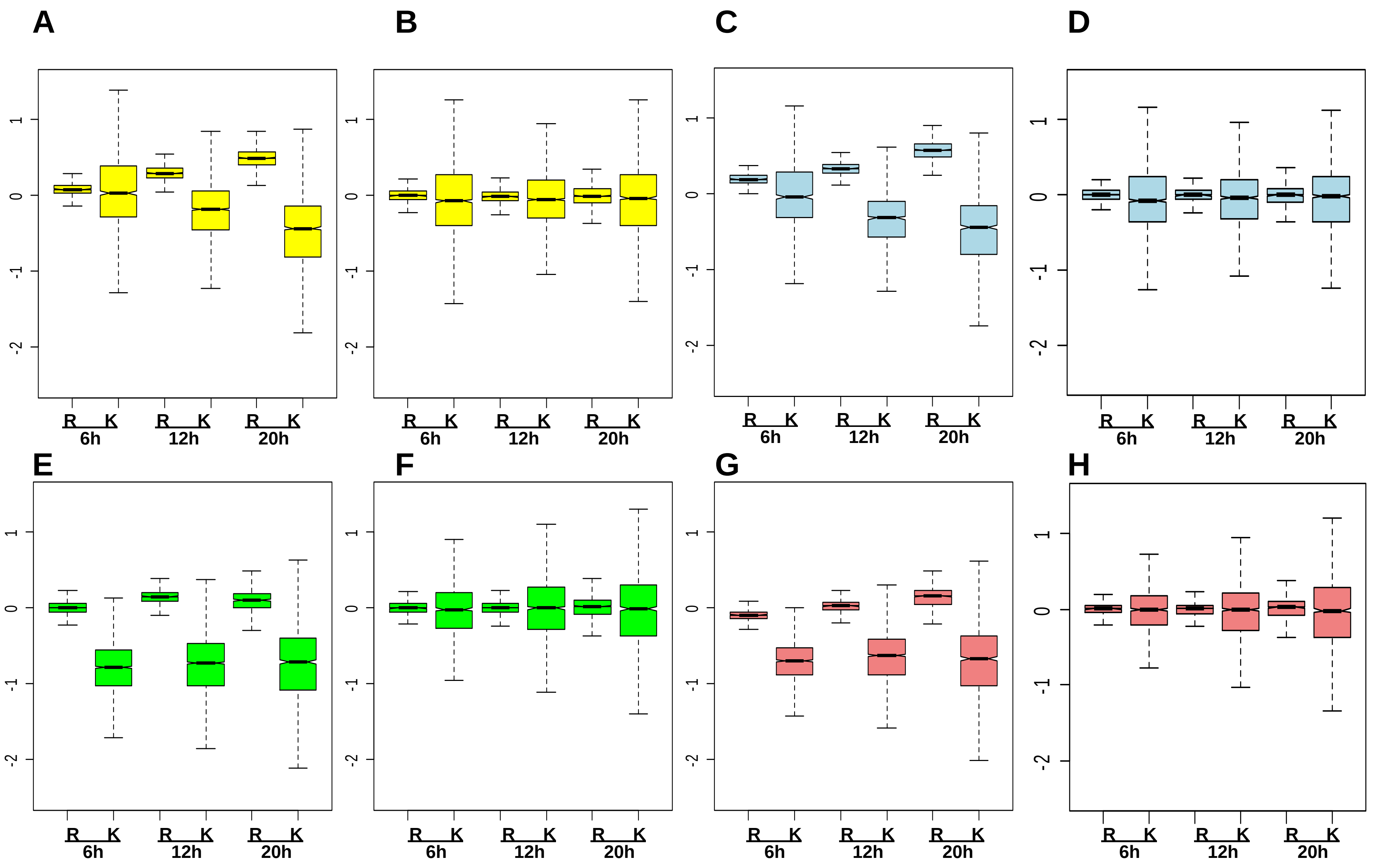

Supplementary figure S9 : global distribution of $(H/M)_K$, $(H/M)_R$ ratios measured in pcSILAC experiments 1 and 2

Boxplots of global $(H/M)_K$, $(H/M)_R$ ratios for replicate 1 of pcSILAC experiment 1, (yellow), replicate 1 of experiment 2 (blue), replicate 2 of experiment 2 (green), replicate 2 of experiment 2 (pink). “R” stands for $(H/M)_R$, “K” for $(H/M)_K$. **A), C), E), G)** show values after correction for mixing ratio only. **B), D), F), H)** show values after correction for mixing ratio and normalization. Ratios were inverted whenever necessary to facilitate comparison between replicates with inverse treatment.

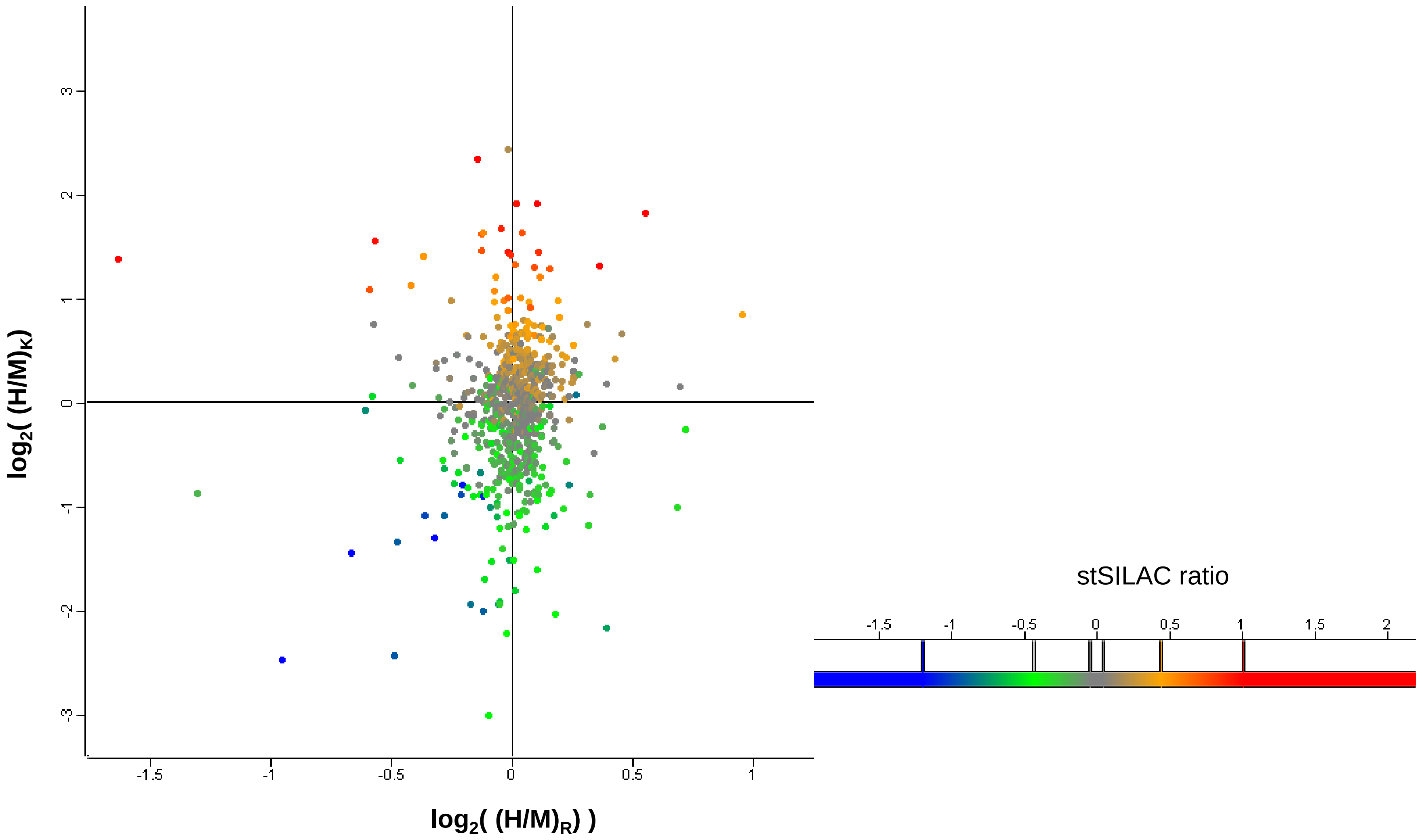

Supplementary figure S10. $(H/M)_K$, $(H/M)_R$ ratios from pcSILAC experiment 1 (t=20h) and their relationship with stSILAC (net protein) values
The values of $(H/M)_K$, $(H/M)_R$ (median of two replicates) at t=20h are represented as scatter plot, with color coding according to the medians at t=20h obtained for the same protein from an internal (carried out simultaneously, with the same cells) stSILAC experiment. A degree of correlation is observed, stronger between $(H/M)_K$ and standard SILAC values. However, similar values in std SILAC experiments can correspond to different combinations of $(H/M)_K$, $(H/M)_R$ values.

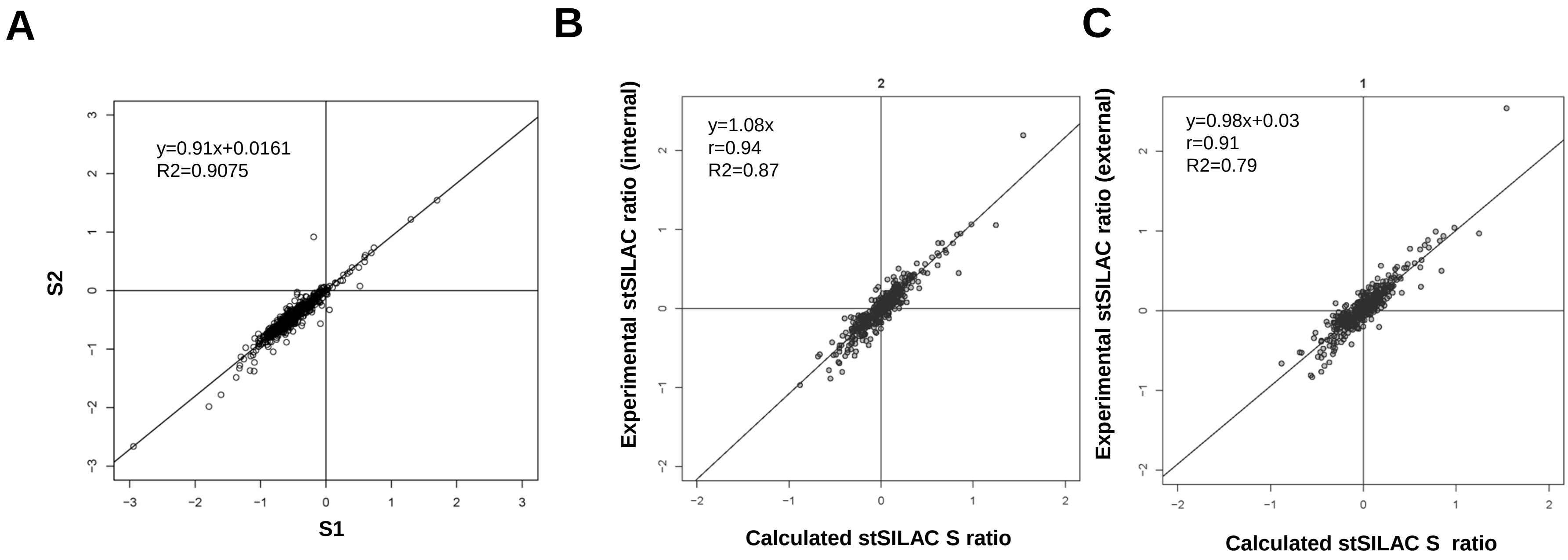

Supplementary Figure S11 : Calculation of a net protein ratio S from pcSILAC data and correlation with net protein ratios determined experimentally through independent experiments.

A) correlation between theoretical S_1 and S_2 values (from pcSILAC data) obtained with two complementary equations (eq.5 and eq.6 in main manuscript) using different ratios. Data were from replicate 2 of experiment 1, not corrected for mixing ratio inequalities
B) Correlation of S (calculated as average of S_1, S_2, normalized) with experimental standard SILAC (net protein) ratios from a parallel experiment performed simultaneously (« internal » control) or **C)** an independent standard SILAC experiment performed 6 weeks earlier. Log_2 have been applied on median-centered values of ratios. Data are from pcSILAC experiment 1.

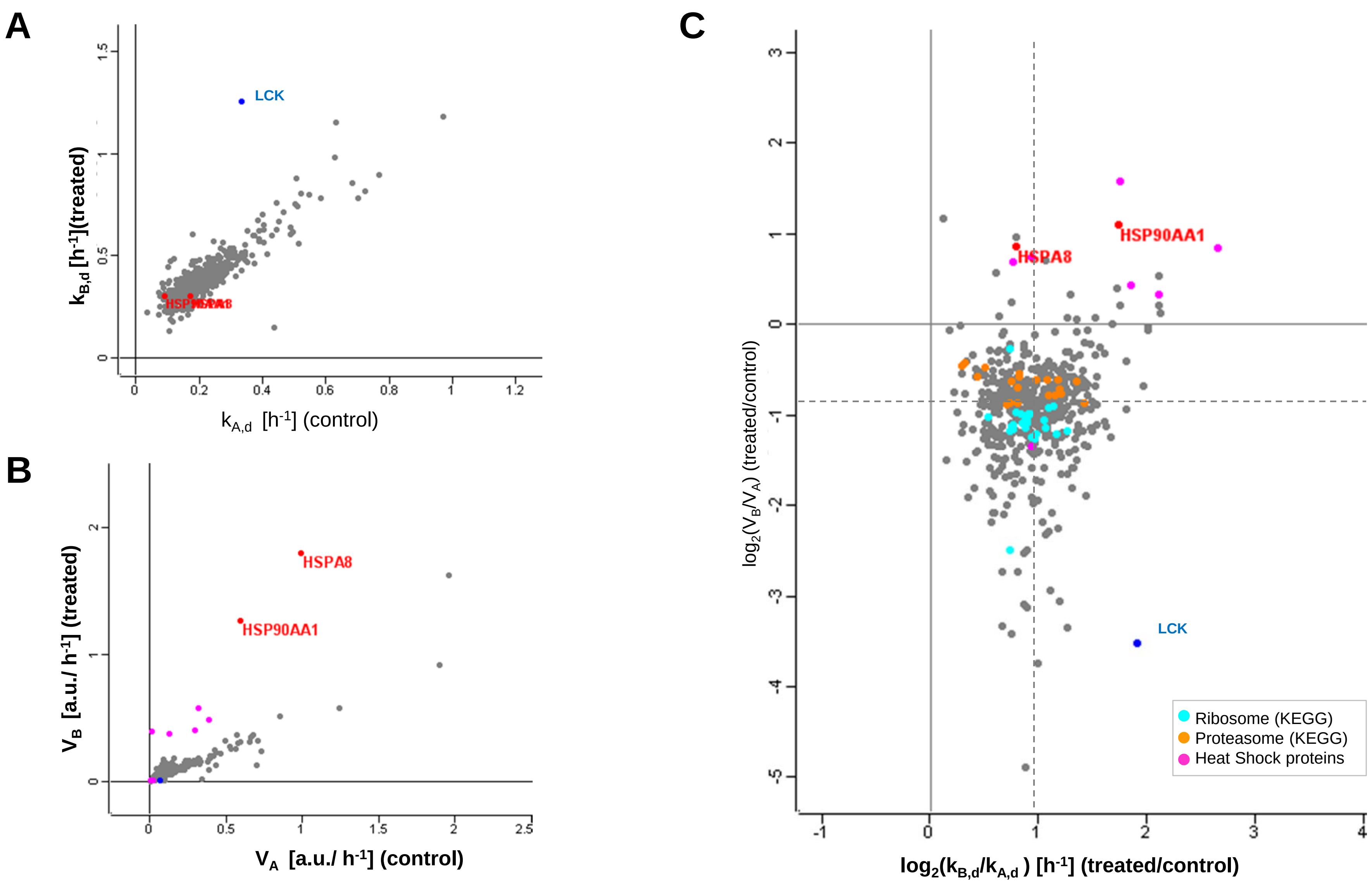

Supplementary Figure S12. Results from calculations of of kinetic parameters for pcSILAC experiment 1 (same plots as in main Figures 4-5, other experiment)
A) Scatter plot of decay constants for the control and treated sample (experiment 1, 520 proteins). The position of reference proteins is indicated. **B)** scatter plot of Vs_control and Vs_treated in the same dataset. Other heat shock proteins are shown in pink **C)** Scatter plot of the values of ratios of intrinsic degradation constants $k_{B,d}/k_{A,d}$ vs. the ratios of synthesis rates V_B/V_A. The median values of $k_{B,d}/k_{A,d}$ and V_B/V_A for the population are indicated with dashed lines. Coloring of points indicates ribosomal, proteasome and heat shock proteins.

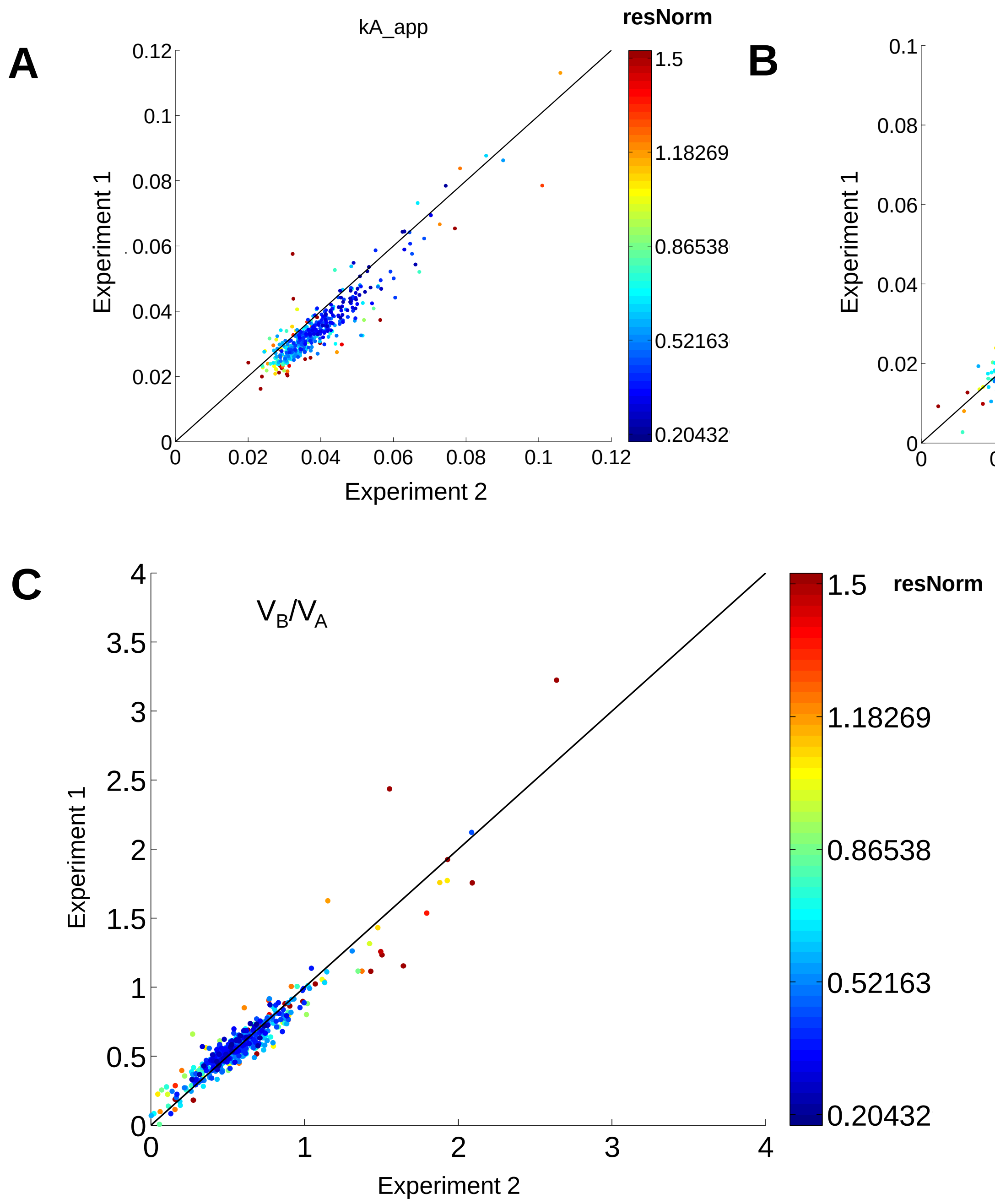

Supplementary figure S13. Comparing the fitting and the resulting $k_{d,app}$ and V_B/V_A ratios from two pcSILAC experiments

Values of kinetic parameters for 462 proteins fully quantified in both pcSILAC experiments are shown, together with the value of the residual norm of the error (sum parameter ResNorm for both experiments). ResNorm gives a measure of the quality of the fit between experimental data and the model. Proteins with lower summed resNorm, indicating a good fit, are usually closer to the diagonal than the ones with higher values of resNorm.

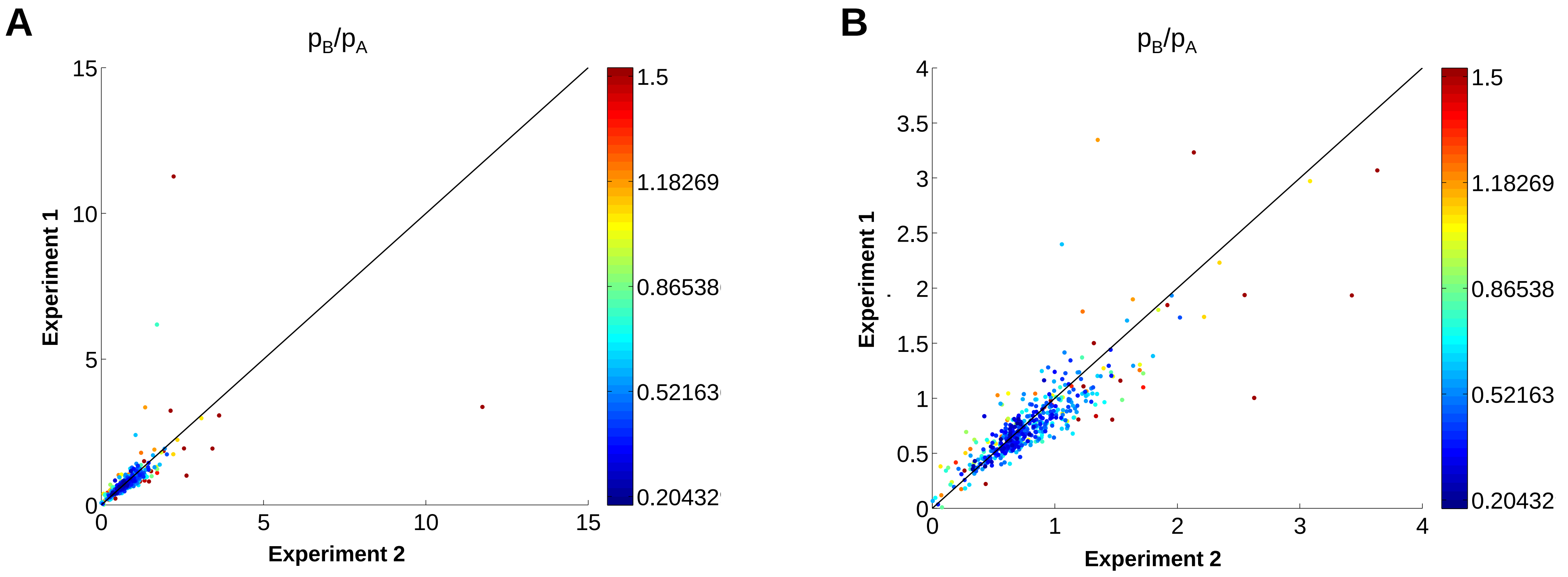

Supplementary figure S14. Comparing the fitting and the resulting steady state values of protein concentrations in control and treated samples from two pcSILAC experiments

A) Values of predicted steady-state concentrations for 462 proteins fully quantified in both pcSILAC experiments are shown, color-coded by the value of the residual norm of the error (resNorm parameter, sum for both experiments). ResNorm gives a measure of the quality of the fit between experimental data and the model. Proteins with lower summed resNorm , indicating a good fit, are usually closer to the diagonal than the ones with higher values of resNorm. **B)** is an enlargment of the plot in A)

A

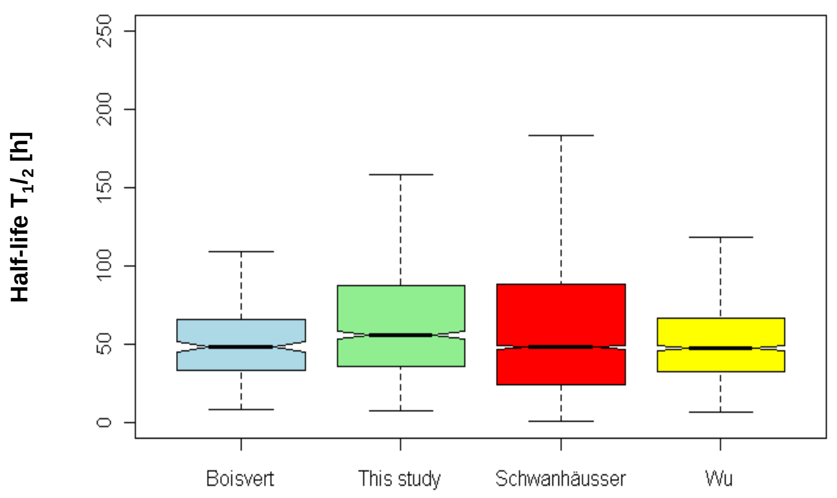

B

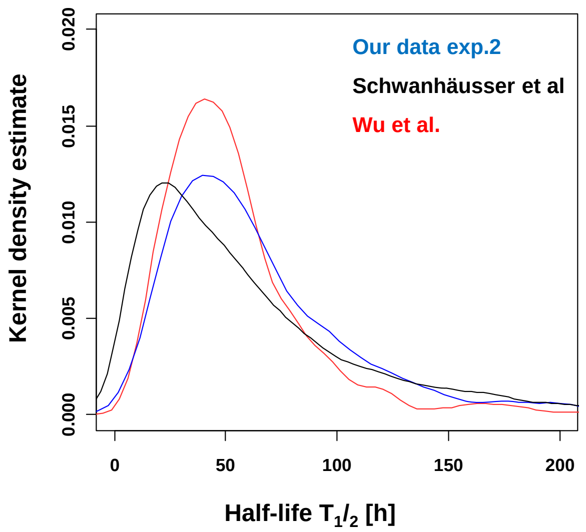

C

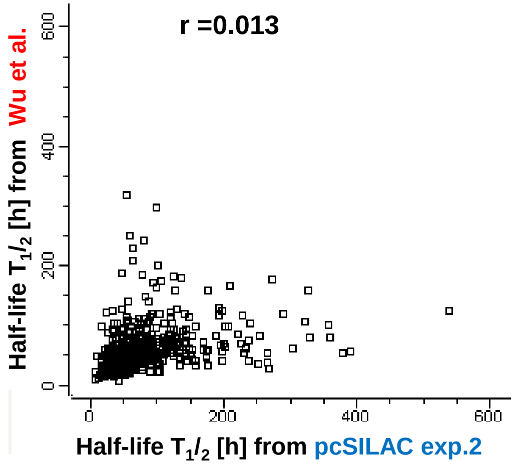

Supplementary Figure S15 . Comparison of half lives obtained by pcSILAC with published datasets

Half lives were retrieved from the supplementary data of three published works and are compared with data from pcSILAC experiment 2 (untreated cells). Protein groups were matched by gene name. The data were acquired on the following cell lines ; Wu et al : CAL27 , oral adenosquamous carcinoma ; Schwanhäusser et al : mouse NIH 3T3 fibroblasts ; Boisvert et al: HeLa cells (whole cell half life dataset).

A) boxplot of the four datasets (outliers not shown) Note : the dataset of Boisvert et al contained a large number of stable proteins for which half-lives could not be calculated accurately and were given a value of 999. These were not used for this plot **B)** Kernel density estimate for three out of the four datasets **C)** scatterplot and Pearson's correlation coefficient of values from pcSILAC against those reported by Wu et al .

References

1. Boisvert, F.-M., Ahmad, Y., Gierlinski, M., Charrière, F., Lamont, D., Scott, M., Barton, G., et al. (2012). Molecular & cellular proteomics : MCP, 11(3)
2. Wu, Z., Moghaddas Gholami, A., & Kuster, B. (2012). Molecular & cellular proteomics : MCP, 11(6),
3. Schwanhäusser, B., Busse, D., Li, N., Dittmar, G., Schuchhardt, J., Wolf, J., Chen, W., et al. (2011). Nature, 473(7347), 337–42.

A

pcSILAC exp.1

kA,d	kB,d	log2(vB/vA)	Name
-0.06	-0.21	0.88	log2(P_B/P_A)
-0.21	-0.28	0.75	log2(avg S1,S2) at 20h
-0.20	-0.10	0.82	log2(HM) 20h

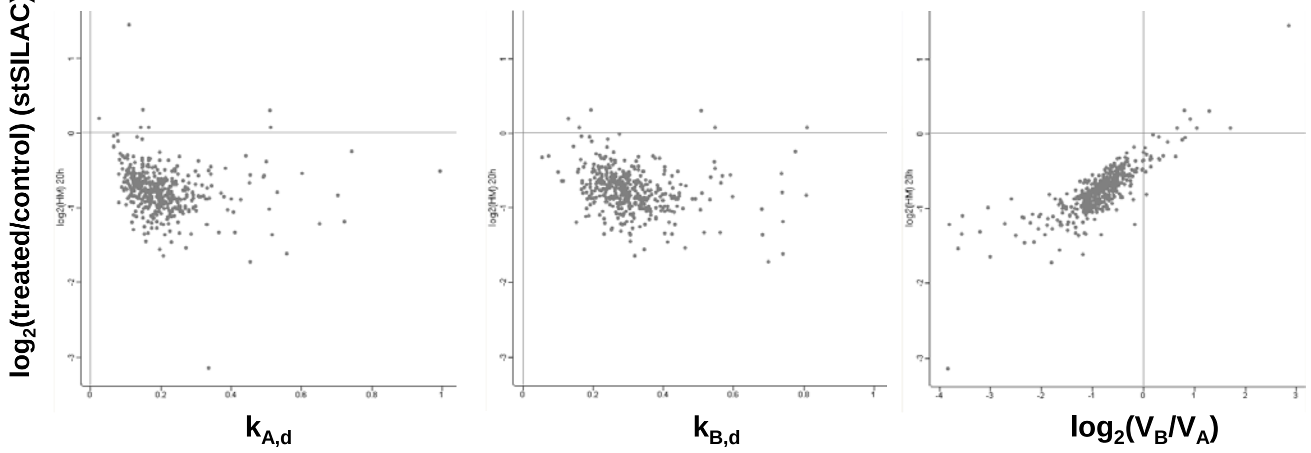

B

pcSILAC exp.2

kA,d	kB,d	log2(vB/vA)	Name
-0.352	-0.469	0.715	log2(avg(S1,S2 at 20h)
-0.247	-0.343	0.882	log2(P_B / P_A)

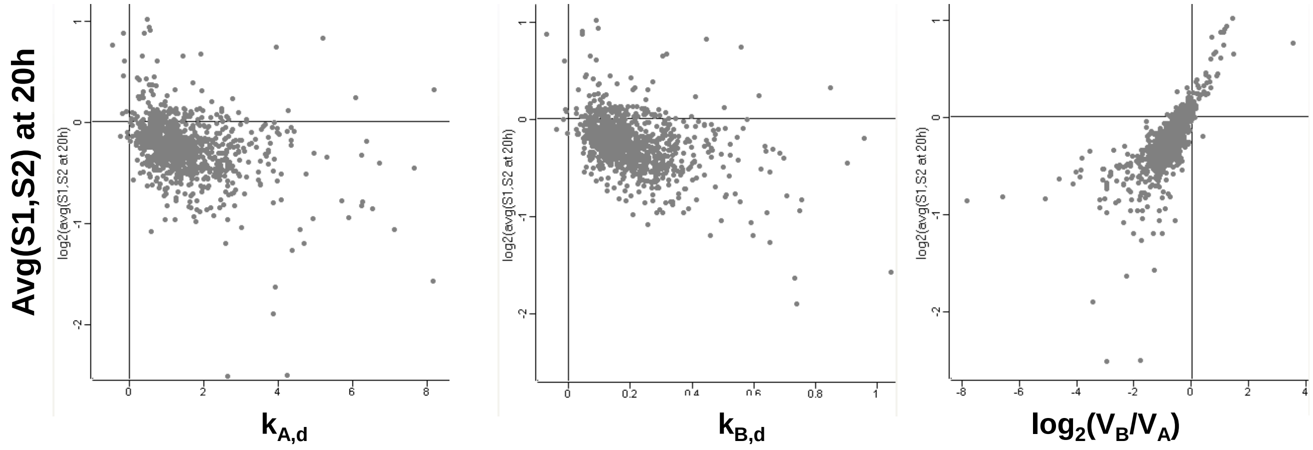

Supplementary Figure S16 Correlation between synthesis rates, decay constants and changes in net protein levels in the two pcSILAC experiments

Pearson's *r* values are shown. Plots represent the datasets highlighted in yellow in the tables.

A) Correlation of k_A, k_B and $\log_2(V_B/V_A)$ with the experimentally determined stSILAC ratio at 20h in experiment 1. **B)** Correlation of k_A, k_B and $\log_2(V_B/V_A)$ with computationally derived ratio of net protein levels in experiment 2 (20h).

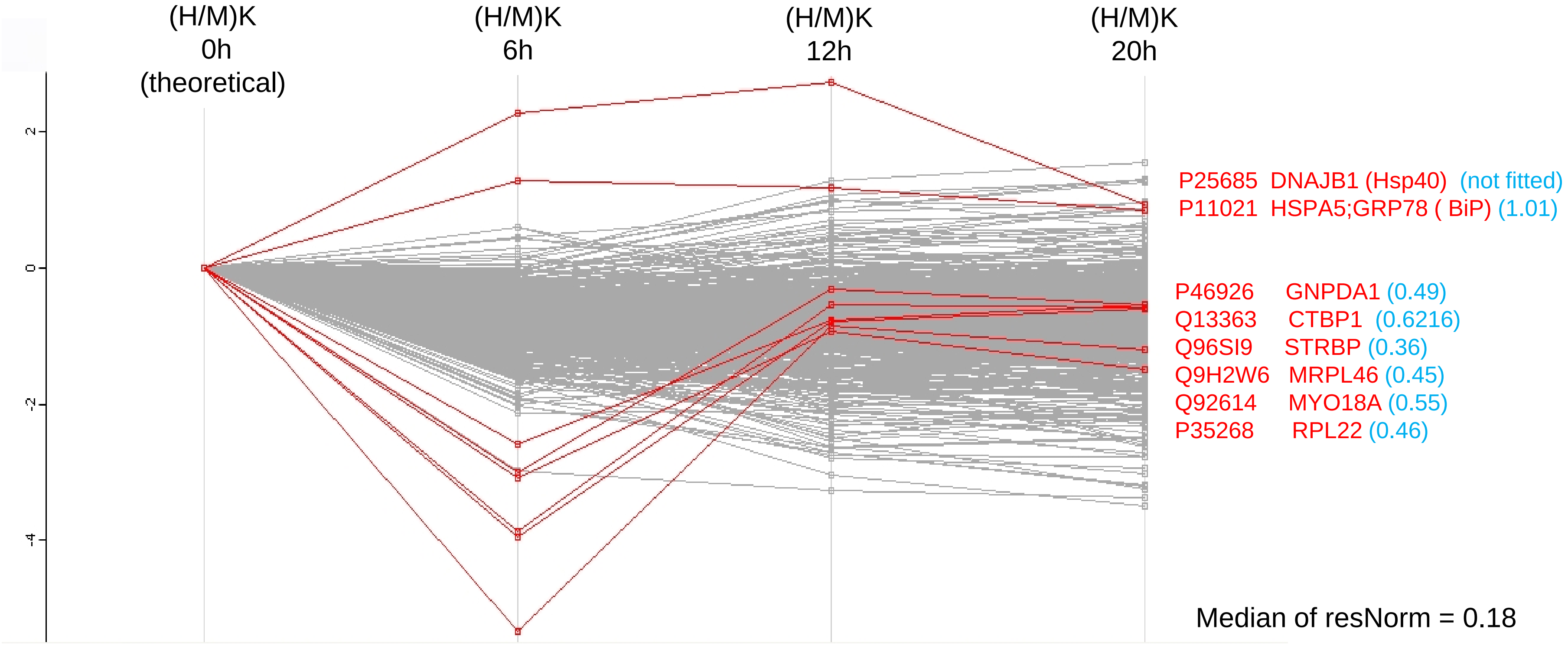

Supplementary Figure S17 Example of proteins with poor fitting to the model

Ratios (H/M)K , measuring newly synthesized protein levels from experiment 2 are shown. Values are after correction for mixing inequality and log2 transformation. Some proteins with apparent multiphasic behavior are shown in red, together with their value of the ResNorm parameter (Blue), which is a measure of the quality of the fitting. Most proteins highlighted here have values of ResNorm well above the median (0.18) of ResNorm for the whole population. It is to be noted however that, since the fitting is performed on 6 series of ratios, deviations due to noise in one series can be compensated by other values. DNAJB1 and GRP78 were quantitated with large numbers of peptides and thus their values should be reliable and reflect a complex temporal dynamic of changes.
